# Supplementary material for: Engineering a scalable and orthogonal platform for synthetic communication in mammalian cells
Source: Nat Commun. 2023 Nov 2;14:7001. doi: 10.1038/s41467-023-42810-5 (PMC10622552; doi:10.1038/s41467-023-42810-5)
Supplement: Supplementary file 1 — Supplementary Information [file 41467_2023_42810_MOESM1_ESM.pdf]

Supplementary Tables

Supplementary Table S1: DNA and protein sequences of unique monomeric CC modules. Overview of nucleotide and amino acid sequences of monomeric CCs (A, A', B, B', Γ, and Γ').

ID	DNA Sequence (5' to 3')	Amino acid sequence (N-term to C-term)
**A**	GAGATCCAGCAACTGGAAGAGGAAATCGCCCAGCTGGAGCAGAAAAACGCCGCTCTGAAGGAAAAGAACCAGGCCCTGAAGTAC	EIQQLEEEIAQLEQKNAALKEKNQALKY
**A'**	AAAATTGCGCAGTTGAAACAAAAAATTCAGGCGCTCAAGCAAGAAAATCAACAGTTGGAGGAGGAGAATGCAGCCCTGGAGTAT	KIAQLKQKIQALKQENQQLEEENAALEY
**B**	GAGAACGCCGCTCTGGAAGAGAAGATCGCCCAGCTGAAACAGAAGAACGCCGCCCTGAAGGAAGAGATCCAGGCCCTGGAATAC	ENAALEEKIAQLKQKNAALKEEIQALEY
**B'**	AAGAACGCCGCTCTGAAGGAAGAGATCCAGGCGTTGGAAGAGGAGAACCAGGCCCTGGAAGAGAAGATCGCCCAGCTGAAATAC	KNAALKEEIQALEEENQALEEKIAQLKY
**Γ**	GAGATCCAAGCCCTGGAAGAGAAGAACGCCCAGCTGAAACAGGAGATCGCCGCTCTGGAAGAAAAGAACCAGGCCCTGAAGTAC	EIQALEEKNAQLKQEIAALEEKNQALKY
**Γ'**	AAGATCGCCCAGCTGAAGGAAGAGAACCAGCAACTGGAACAGAAGATCCAGGCCCTGAAAGAGGAAAACGCCGCTCTGGAGTAC	KIAQLKEENQQLEQKIQALKEENAALEY

Supplementary Table S2: DNA and protein sequences for linker α_x. Three different protein linkers (α_x) used to span the CC and EpoR for the construction of the CC-GEMS receptor.

ID (α_x)	DNA Sequence (5' to 3')	Amino acid sequence (N-term to C-term)
α_1	GGATCAGGGTCTGGAAGTGGGAGT	GSGS
α_2	GGATCAGGGTCTGGAAGTGGGAGTGGATCAGGGTCTGGAAGTGGGAGT	GSGSGSGS
α_3	AGTGGATCCTCGGGATCATCAGGATCCTCTGGGAGCTCAGGATCCTCGGGGAGCTCTGGATCCAGCGGTTCAAGCGGATCC	SGSSGSSGSSGSSGSSGSSGSSGSSGS

Supplementary Table S3: Plasmid backbones used in the present study. Overview of plasmid backbones used and corresponding source. Multiple unique plasmids were designed by recombination of unique CC modules and linkers.

ID	Description	Source
pLS13	Mammalian reporter plasmid for STAT3-induced SEAP expression (O_{Stat3}-$P_{hCMVmin}$-SEAP-pA).	Schukur et al.[1] Plasmid was kindly provided by Martin Fussenenger.
pLS15	Mammalian STAT3 expression vector (P_{hCMV}-STAT3-pA).	Schukur et al.[1] Plasmid was kindly provided by Martin Fussenenger.
pHY30	Mammalian reporter plasmid for VEGFR2-induced SEAP expression (O_{NFAT}-$P_{hCMVmin}$-SEAP-pA).	Ye et al.[2]
pLeo619-P_{SV40}	Mammalian pLeo619 expression vector to recombinantly express the EpoR$_m$-IL-6RB$_m$-pA fused to a selected CC module.	Original plasmid was kindly provided by Martin Fussenenger (GenBank accession no. MG437012)[3].

pET28a(+)-P$_{T7}$	Bacterial Pet28a(+) expression vector to recombinantly express different variants of monomeric or ditopic SUMO-CC fusion proteins in *E. Coli*.	
pHR-P$_{CMV}$	Second generation lentiviral transfer pHR-CMV expression vector for generation of lentiviruses.	Gift from A. Radu Arocescu (Addgene plasmid #113891; http://n2t.net/addgene:113891; RRID:Addgene_113891)[4].

Supplementary Table S4: P-values, F-values and degrees of freedom for One-Way ANOVA. Overview of p-values, F-values and degrees of freedom (df) for unpaired, one-way ANOVA with Tukey's, Šidák's or Dunnett's multiple comparison test, indicating the compared groups and corresponding Figure. n.s.: (non-significant), *p ≤0.05, **p ≤0.01, *** p ≤0.001.

Figure	Compared groups (ANOVA)	F-value	p-value summary (ANOVA)	Compared groups (multiple comparisons)	adjusted p-value (multiple comparisons)	df
1d	EGFP vs. A-GEMS$_{JAK/STAT}$:A-GEMS$_{JAK/STAT}$ vs. A'-GEMS$_{JAK/STAT}$:A'-GEMS$_{JAK/STAT}$ vs. A-GEMS$_{JAK/STAT}$:B-GEMS$_{JAK/STAT}$ vs. A-GEMS$_{JAK/STAT}$:A'-GEMS$_{JAK/STAT}$ (0 aa) vs. A-GEMS$_{JAK/STAT}$:A'-GEMS$_{JAK/STAT}$ (4 aa) vs. A-GEMS$_{JAK/STAT}$:A'-GEMS$_{JAK/STAT}$ (8 aa) vs. A-GEMS$_{JAK/STAT}$:A'-GEMS$_{JAK/STAT}$ (27 aa)	91.75	*** (p<0.0001)	EGFP vs. A-GEMS$_{JAK/STAT}$:A-GEMS$_{JAK/STAT}$	ns (p>0.9999) Tukey	23
				EGFP vs. A'-GEMS$_{JAK/STAT}$:A'-GEMS$_{JAK/STAT}$	ns (p>0.9999) Tukey	
				EGFP vs. A-GEMS$_{JAK/STAT}$:B-GEMS$_{JAK/STAT}$	ns (p>0.9999) Tukey	
				EGFP vs. A-GEMS$_{JAK/STAT}$:A'-GEMS$_{JAK/STAT}$ (0 aa)	*** (p<0.0001) Tukey	
				EGFP vs. A-GEMS$_{JAK/STAT}$:A'-GEMS$_{JAK/STAT}$ (4 aa)	*** (p<0.0001) Tukey	
				EGFP vs. A-GEMS$_{JAK/STAT}$:A'-GEMS$_{JAK/STAT}$ (8 aa)	*** (p<0.0001) Tukey	
				EGFP vs. A-GEMS$_{JAK/STAT}$:A'-GEMS$_{JAK/STAT}$ (27 aa)	*** (p<0.0001) Tukey	
				A-GEMS$_{JAK/STAT}$:A-GEMS$_{JAK/STAT}$ vs. A'-GEMS$_{JAK/STAT}$:A'-GEMS$_{JAK/STAT}$	ns (p>0.9999) Tukey	
				A-GEMS$_{JAK/STAT}$:A-GEMS$_{JAK/STAT}$ vs.	ns (p>0.9999) Tukey	

				A-GEMS$_{JAK/STAT}$:B-GEMS$_{JAK/STAT}$		
				A-GEMS$_{JAK/STAT}$:A-GEMS$_{JAK/STAT}$ vs. A-GEMS$_{JAK/STAT}$:A'-GEMS$_{JAK/STAT}$ (0 aa)	*** (p<0.0001) Tukey	
				A-GEMS$_{JAK/STAT}$:A-GEMS$_{JAK/STAT}$ vs. A-GEMS$_{JAK/STAT}$:A'-GEMS$_{JAK/STAT}$ (4 aa)	*** (p<0.0001) Tukey	
				A-GEMS$_{JAK/STAT}$:A-GEMS$_{JAK/STAT}$ vs. A-GEMS$_{JAK/STAT}$:A'-GEMS$_{JAK/STAT}$ (8 aa)	*** (p<0.0001) Tukey	
				A-GEMS$_{JAK/STAT}$:A-GEMS$_{JAK/STAT}$ vs. A-GEMS$_{JAK/STAT}$:A'-GEMS$_{JAK/STAT}$ (27 aa)	*** (p<0.0001) Tukey	
				A'-GEMS$_{JAK/STAT}$:A'-GEMS$_{JAK/STAT}$ vs. A-GEMS$_{JAK/STAT}$:B-GEMS$_{JAK/STAT}$	ns (p>0.9999) Tukey	
				A'-GEMS$_{JAK/STAT}$:A'-GEMS$_{JAK/STAT}$ vs. A-GEMS$_{JAK/STAT}$:A'-GEMS$_{JAK/STAT}$ (0 aa)	*** (p<0.0001) Tukey	
				A'-GEMS$_{JAK/STAT}$:A'-GEMS$_{JAK/STAT}$ vs. A-GEMS$_{JAK/STAT}$:A'-GEMS$_{JAK/STAT}$ (4 aa)	*** (p<0.0001) Tukey	
				A'-GEMS$_{JAK/STAT}$:A'-GEMS$_{JAK/STAT}$ vs. A-GEMS$_{JAK/STAT}$:A'-GEMS$_{JAK/STAT}$ (8 aa)	*** (p<0.0001) Tukey	
				A'-GEMS$_{JAK/STAT}$:A'-GEMS$_{JAK/STAT}$ vs. A-GEMS$_{JAK/STAT}$:A'-GEMS$_{JAK/STAT}$ (27 aa)	*** (p<0.0001) Tukey	
				A'-GEMS$_{JAK/STAT}$:B-GEMS$_{JAK/STAT}$ vs. A-GEMS$_{JAK/STAT}$:A'-GEMS$_{JAK/STAT}$ (0 aa)	*** (p<0.0001) Tukey	
				A'-GEMS$_{JAK/STAT}$:B-GEMS$_{JAK/STAT}$ vs.	*** (p<0.0001) Tukey	

				A-GEMS$_{JAK/STAT}$:A’-GEMS$_{JAK/STAT}$ (4 aa)		
				A’-GEMS$_{JAK/STAT}$:B-GEMS$_{JAK/STAT}$ vs. A-GEMS$_{JAK/STAT}$:A’-GEMS$_{JAK/STAT}$ (8 aa)	*** (p<0.0001) Tukey	
				A’-GEMS$_{JAK/STAT}$:B-GEMS$_{JAK/STAT}$ vs. A-GEMS$_{JAK/STAT}$:A’-GEMS$_{JAK/STAT}$ (27 aa)	*** (p<0.0001) Tukey	
				A-GEMS$_{JAK/STAT}$:A’-GEMS$_{JAK/STAT}$ (0 aa) vs. A-GEMS$_{JAK/STAT}$:A’-GEMS$_{JAK/STAT}$ (4 aa)	ns (p=0.9959) Tukey	
				A-GEMS$_{JAK/STAT}$:A’-GEMS$_{JAK/STAT}$ (0 aa) vs. A-GEMS$_{JAK/STAT}$:A’-GEMS$_{JAK/STAT}$ (8 aa)	ns (p=0.9995) Tukey	
				A-GEMS$_{JAK/STAT}$:A’-GEMS$_{JAK/STAT}$ (0 aa) vs. A-GEMS$_{JAK/STAT}$:A’-GEMS$_{JAK/STAT}$ (27 aa)	ns (p=0.3238) Tukey	
				A-GEMS$_{JAK/STAT}$:A’-GEMS$_{JAK/STAT}$ (4 aa) vs. A-GEMS$_{JAK/STAT}$:A’-GEMS$_{JAK/STAT}$ (8 aa)	ns (p=0.9223) Tukey	
				A-GEMS$_{JAK/STAT}$:A’-GEMS$_{JAK/STAT}$ (4 aa) vs. A-GEMS$_{JAK/STAT}$:A’-GEMS$_{JAK/STAT}$ (27 aa)	ns (p=0.7110) Tukey	
				A-GEMS$_{JAK/STAT}$:A’-GEMS$_{JAK/STAT}$ (8 aa) vs. A-GEMS$_{JAK/STAT}$:A’-GEMS$_{JAK/STAT}$ (27 aa)	ns (p=0.1489) Tukey	
S6f	B-GEMS$_{JAK/STAT}$ with A’-A’ vs. B-GEMS$_{JAK/STAT}$ with A’-A’ (**v**$_2$) vs. B-GEMS$_{JAK/STAT}$ with A’-A’ (**v**$_3$)	2.562	ns (p=0.160)	-	-	8
S6f	A-GEMS$_{JAK/STAT}$ ($\boldsymbol{\alpha}$=0 aa) with A’-A’ vs. A-GEMS$_{JAK/STAT}$ ($\boldsymbol{\alpha}$=0 aa) with A’-A’ (**v**$_2$) vs. A-GEMS$_{JAK/STAT}$ ($\boldsymbol{\alpha}$=0 aa) with A’-A’ (**v**$_3$)	22.95	** (p=0.0015)	A-GEMS$_{JAK/STAT}$ ($\boldsymbol{\alpha}$=0 aa) with A’-A’ vs. A-GEMS$_{JAK/STAT}$ ($\boldsymbol{\alpha}$=0 aa) with A’-A’ (**v**$_2$)	ns (p=0.2534) Tukey	8
				A-GEMS$_{JAK/STAT}$ ($\boldsymbol{\alpha}$=0 aa) with A’-A’ vs.	** (p=0.0015) Tukey	

				A-GEMS$_{JAK/STAT}$ (**α**=0 aa) with A’-A’ (**v_3**)		
				A-GEMS$_{JAK/STAT}$ (**α**=0 aa) with A’-A’ (**v_2**)<br>vs.<br>A-GEMS$_{JAK/STAT}$ (**α**=0 aa) with A’-A’ (**v_3**)	** (p=0.0074)<br>Tukey	
S6f	A-GEMS$_{JAK/STAT}$ (**α_1**=4 aa) with A’-A’<br>vs.<br>A-GEMS$_{JAK/STAT}$ (**α_1**=4 aa) with A’-A’ (**v_2**)<br>vs.<br>A-GEMS$_{JAK/STAT}$ (**α_1**=4 aa) with A’-A’ (**v_3**)	1.739	ns<br>(p=0.2536)	-	-	8
S6f	A-GEMS$_{JAK/STAT}$ (**α_2**=8 aa) with A’-A’<br>vs.<br>A-GEMS$_{JAK/STAT}$ (**α_2**=8 aa) with A’-A’ (**v_2**)<br>vs.<br>A-GEMS$_{JAK/STAT}$ (**α_2**=8 aa) with A’-A’ (**v_3**)	5.317	*<br>(p=0.0469)	A-GEMS$_{JAK/STAT}$ (**α_2**=8 aa) with A’-A’<br>vs.<br>A-GEMS$_{JAK/STAT}$ (**α_2**=8 aa) with A’-A’ (**v_2**)	ns (p=0.0689)<br>Tukey	8
				A-GEMS$_{JAK/STAT}$ (**α_2**=8 aa) with A’-A’<br>vs.<br>A-GEMS$_{JAK/STAT}$ (**α_2**=8 aa) with A’-A’ (**v_3**)	* (p=0.0666)<br>Tukey	
				A-GEMS$_{JAK/STAT}$ (**α_2**=8 aa) with A’-A’ (**v_2**)<br>vs.<br>A-GEMS$_{JAK/STAT}$ (**α_2**=8 aa) with A’-A’ (**v_3**)	ns (p=0.9996)<br>Tukey	
S6f	A-GEMS$_{JAK/STAT}$ (**α_3**=27 aa) with A’-A’<br>vs.<br>A-GEMS$_{JAK/STAT}$ (**α_3**=27 aa) with A’-A’ (**v_2**)<br>vs.<br>A-GEMS$_{JAK/STAT}$ (**α_3**=27 aa) with A’-A’ (**v_3**)	13.50	**<br>(p=0.006)	A-GEMS$_{JAK/STAT}$ (**α_3**=27 aa) with A’-A’<br>vs.<br>A-GEMS$_{JAK/STAT}$ (**α_3**=27 aa) with A’-A’ (**v_2**)	ns (p=0.0519)<br>Tukey	8
				A-GEMS$_{JAK/STAT}$ (**α_3**=27 aa) with A’-A’<br>vs.<br>A-GEMS$_{JAK/STAT}$ (**α_3**=27 aa) with A’-A’ (**v_3**)	** (p=0.005)<br>Tukey	
				A-GEMS$_{JAK/STAT}$ (**α_3**=27 aa) with A’-A’ (**v_2**)<br>vs.<br>A-GEMS$_{JAK/STAT}$ (**α_3**=27 aa) with A’-A’ (**v_3**)	ns (p=0.1632)<br>Tukey	
3c	A-GEMS$_{JAK/STAT}$ (**α_2**=8 aa) with A’-A’<br>vs.<br>A-GEMS$_{JAK/STAT}$ (**α_2**=8 aa) with A’<br>vs.<br>A-GEMS$_{JAK/STAT}$ (**α_2**=8 aa) with SUMO.A’-A’ (**l_1**)<br>vs.	29.33	***<br>(p<0.0001)	A-GEMS$_{JAK/STAT}$ (**α_2**=8 aa) with SUMO.A’-A’ (**l_1**)<br>vs.<br>A-GEMS$_{JAK/STAT}$ (**α_2**=8 aa) with A’-A’ (**l_1**)	ns (p=0.1749)<br>Šidák	23
				A-GEMS$_{JAK/STAT}$ (**α_2**=8 aa) with SUMO.A’-A’ (**l_2**)<br>vs.	ns (p=0.5304)<br>Šidák	

	A-GEMS$_{JAK/STAT}$ (α_2=8 aa) with A′-A′ (l_1)<br>vs.<br>A-GEMS$_{JAK/STAT}$ (α_2=8 aa) with SUMO.A′-A′ (l_2)<br>vs.<br>A-GEMS$_{JAK/STAT}$ (α_2=8 aa) with A′-A′ (l_2)<br>vs.<br>A-GEMS$_{JAK/STAT}$ (α_2=8 aa) with SUMO.A′-A′ (l_3)<br>vs.<br>A-GEMS$_{JAK/STAT}$ (α_2=8 aa) with A′-A′ (l_3)			A-GEMS$_{JAK/STAT}$ (α_2=8 aa) with A′-A′ (l_2)		
				A-GEMS$_{JAK/STAT}$ (α_2=8 aa) with SUMO.A′-A′ (l_3)<br>vs.<br>A-GEMS$_{JAK/STAT}$ (α_2=8 aa) with A′-A′ (l_3)	ns (p=0.9694)<br>Šidák	
5a	A-GEMS$_{JAK/STAT}$ (α_2=8 aa) with A′-A′<br>vs.<br>A-GEMS$_{JAK/STAT}$ (α_2=8 aa) with B′-B′<br>vs.<br>A-GEMS$_{JAK/STAT}$ (α_2=8 aa) with Γ′-Γ′	285.1	***<br>(p<0.0001)	A-GEMS$_{JAK/STAT}$ (α_2=8 aa) with A′-A′<br>vs.<br>A-GEMS$_{JAK/STAT}$ (α_2=8 aa) with B′-B′	*** (p<0.0001)<br>Tukey	8
				A-GEMS$_{JAK/STAT}$ (α_2=8 aa) with A′-A′<br>vs.<br>A-GEMS$_{JAK/STAT}$ (α_2=8 aa) with Γ′-Γ′	*** (p<0.0001)<br>Tukey	
				A-GEMS$_{JAK/STAT}$ (α_2=8 aa) with B′-B′<br>vs.<br>A-GEMS$_{JAK/STAT}$ (α_2=8 aa) with Γ′-Γ′	ns (p=0.9994)<br>Tukey	
5a	B-GEMS$_{JAK/STAT}$ (α_2=8 aa) with A′-A′<br>vs.<br>B-GEMS$_{JAK/STAT}$ (α_2=8 aa) with B′-B′<br>vs.<br>B-GEMS$_{JAK/STAT}$ (α_2=8 aa) with Γ′-Γ′	1155	***<br>(p<0.0001)	B-GEMS$_{JAK/STAT}$ (α_2=8 aa) with A′-A′<br>vs.<br>B-GEMS$_{JAK/STAT}$ (α_2=8 aa) with B′-B′	*** (p<0.0001)<br>Tukey	8
				B-GEMS$_{JAK/STAT}$ (α_2=8 aa) with A′-A′<br>vs.<br>B-GEMS$_{JAK/STAT}$ (α_2=8 aa) with Γ′-Γ′	ns (p=0.7867)<br>Tukey	
				B-GEMS$_{JAK/STAT}$ (α_2=8 aa) with B′-B′<br>vs.<br>B-GEMS$_{JAK/STAT}$ (α_2=8 aa) with Γ′-Γ′	*** (p<0.0001)<br>Tukey	
5a	Γ-GEMS$_{JAK/STAT}$ (α_2=8 aa) with A′-A′<br>vs.<br>Γ-GEMS$_{JAK/STAT}$ (α_2=8 aa) with B′-B′<br>vs.<br>Γ-GEMS$_{JAK/STAT}$ (α_2=8 aa) with Γ′-Γ′	5257	***<br>(p<0.0001)	Γ-GEMS$_{JAK/STAT}$ (α_2=8 aa) with A′-A′<br>vs.<br>Γ-GEMS$_{JAK/STAT}$ (α_2=8 aa) with B′-B′	ns (p=0.8865)<br>Tukey	8
				Γ-GEMS$_{JAK/STAT}$ (α_2=8 aa) with A′-A′<br>vs.	*** (p<0.0001)<br>Tukey	

				Γ-GEMS$_{JAK/STAT}$ (α_2=8 aa) with Γ'-Γ'		
				Γ-GEMS$_{JAK/STAT}$ (α_2=8 aa) with B'-B'<br>vs.<br>Γ-GEMS$_{JAK/STAT}$ (α_2=8 aa) with Γ'-Γ'	*** ($p<0.0001$)<br>Tukey	
S16	A-GEMS$_{JAK/STAT}$ (α_2=8 aa) with A'-A'<br>vs.<br>A-GEMS$_{JAK/STAT}$ (α_2=8 aa) with A'-A', B'-B' and Γ'-Γ'<br>vs.<br>B-GEMS$_{JAK/STAT}$ (α_2=8 aa) A'-A'	696.2	***<br>($p<0.0001$)	A-GEMS$_{JAK/STAT}$ (α_2=8 aa) with A'-A'<br>vs.<br>A-GEMS$_{JAK/STAT}$ (α_2=8 aa) with A'-A', B'-B' and Γ'-Γ'	ns ($p=0.3039$)<br>Šidák	8
S17d	A-Γ-GEMS$_{JAK/STAT}$ (α_2=8 aa) with A'-Γ'<br>vs.<br>A-B-GEMS$_{JAK/STAT}$ (α_2=8 aa) with A'-B'<br>vs.<br>A-B-Γ-GEMS$_{JAK/STAT}$ (α_2=8 aa) with no ligand	745.1	***<br>($p<0.0001$)	A-Γ-GEMS$_{JAK/STAT}$ (α_2=8 aa) with A'-Γ'<br>vs.<br>A-B-Γ-GEMS$_{JAK/STAT}$ (α_2=8 aa) with no ligand	*** ($p<0.0001$)<br>Dunnett	8
				A-B-GEMS$_{JAK/STAT}$ (α_2=8 aa) with A'-B'<br>vs.<br>A-B-Γ-GEMS$_{JAK/STAT}$ (α_2=8 aa) with no ligand	*** ($p<0.0001$)<br>Dunnett	
5c	A-B-Γ-GEMS$_{JAK/STAT}$ (α_2=8 aa) with no ligand<br>vs.<br>A-B-Γ-GEMS$_{JAK/STAT}$ (α_2=8 aa) with A'-B'<br>vs.<br>A-B-Γ-GEMS$_{JAK/STAT}$ (α_2=8 aa) with A'-Γ'<br>vs.<br>A-B-Γ-GEMS$_{JAK/STAT}$ (α_2=8 aa) with A'-B' and A'-Γ'	44.65	***<br>($p<0.0001$)	A-B-Γ-GEMS$_{JAK/STAT}$ (α_2=8 aa) with no ligand<br>vs.<br>A-B-Γ-GEMS$_{JAK/STAT}$ (α_2=8 aa) with A'-B'	*** ($p=0.0005$)<br>Dunnett	11
				A-B-Γ-GEMS$_{JAK/STAT}$ (α_2=8 aa) with no ligand<br>vs.<br>A-B-Γ-GEMS$_{JAK/STAT}$ (α_2=8 aa) with A'-Γ'	** ($p=0.0037$)<br>Dunnett	
				A-B-Γ-GEMS$_{JAK/STAT}$ (α_2=8 aa) with no ligand<br>vs.<br>A-B-Γ-GEMS$_{JAK/STAT}$ (α_2=8 aa) with A'-B' and A'-Γ'	*** ($p<0.0001$)<br>Dunnett	
S18	A-B-Γ-GEMS$_{JAK/STAT}$ (α_2=8 aa) with no ligand<br>vs.<br>A-B-Γ-GEMS$_{JAK/STAT}$ (α_2=8 aa) with A'-B'<br>vs.<br>A-B-Γ-GEMS$_{JAK/STAT}$ (α_2=8 aa) with A'-Γ'<br>vs.	554.7	***<br>($p<0.0001$)	A-B-Γ-GEMS$_{JAK/STAT}$ (α_2=8 aa) with no ligand<br>vs.<br>A-B-Γ-GEMS$_{JAK/STAT}$ (α_2=8 aa) with A'-B'	*** ($p=0.0001$)<br>Dunnett	11
				A-B-Γ-GEMS$_{JAK/STAT}$ (α_2=8 aa) with no ligand<br>vs.	*** ($p=0.0002$)<br>Dunnett	

	A-B-Γ-GEMS$_{JAK/STAT}$ (α_2=8 aa) with A’-B’ and A’-Γ’			A-B-Γ-GEMS$_{JAK/STAT}$ (α_2=8 aa) with A’-Γ’		
				A-B-Γ-GEMS$_{JAK/STAT}$ (α_2=8 aa) with no ligand<br>vs.<br>A-B-Γ-GEMS$_{JAK/STAT}$ (α_2=8 aa) with A’-B’ and A’-Γ’	*** (p<0.0001)<br>Dunnett	
5d	A-GEMS$_{JAK/STAT}$ (α_2=8 aa) with no ligand<br>vs.<br>A-GEMS$_{JAK/STAT}$ (α_2=8 aa) with A’-Γ<br>vs.<br>A-GEMS$_{JAK/STAT}$ (α_2=8 aa) with A’-Γ’<br>vs.<br>A-GEMS$_{JAK/STAT}$ (α_2=8 aa) with A’-Γ and A’-Γ’	2800	***<br>(p<0.0001)	A-GEMS$_{JAK/STAT}$ (α_2=8 aa) with no ligand<br>vs.<br>A-GEMS$_{JAK/STAT}$ (α_2=8 aa) with A’-Γ	ns (p=0.9971)<br>Tukey	11
				A-GEMS$_{JAK/STAT}$ (α_2=8 aa) with no ligand<br>vs.<br>A-GEMS$_{JAK/STAT}$ (α_2=8 aa) with A’-Γ’	ns (p=0.8783)<br>Tukey	
				A-GEMS$_{JAK/STAT}$ (α_2=8 aa) with A’-Γ<br>vs.<br>A-GEMS$_{JAK/STAT}$ (α_2=8 aa) with A’-Γ’	ns (p=0.9454)<br>Tukey	
				A-GEMS$_{JAK/STAT}$ (α_2=8 aa) with A’-Γ and A’-Γ’<br>vs.<br>A-GEMS$_{JAK/STAT}$ (α_2=8 aa) with no ligand	*** (p<0.0001)<br>Tukey	
				A-GEMS$_{JAK/STAT}$ (α_2=8 aa) with A’-Γ and A’-Γ’<br>vs.<br>A-GEMS$_{JAK/STAT}$ (α_2=8 aa) with A’-Γ	*** (p<0.0001)<br>Tukey	
				A-GEMS$_{JAK/STAT}$ (α_2=8 aa) with A’-Γ and A’-Γ’<br>vs.<br>A-GEMS$_{JAK/STAT}$ (α_2=8 aa) with A’-Γ’	*** (p<0.0001)<br>Tukey	
S25b	A-GEMS$_{JAK/STAT}$ (α_2=8 aa) with no ligand<br>vs.<br>A-GEMS$_{JAK/STAT}$ (α_2=8 aa) with A’-Γ<br>vs.<br>A-GEMS$_{JAK/STAT}$ (α_2=8 aa) with A’-Γ’<br>vs.<br>A-GEMS$_{JAK/STAT}$ (α_2=8 aa) with A’-Γ and A’-Γ’	2744	***<br>(p<0.0001)	A-GEMS$_{JAK/STAT}$ (α_2=8 aa) with no ligand<br>vs.<br>A-GEMS$_{JAK/STAT}$ (α_2=8 aa) with A’-Γ	ns (p=0.9787)<br>Tukey	11
				A-GEMS$_{JAK/STAT}$ (α_2=8 aa) with no ligand<br>vs.<br>A-GEMS$_{JAK/STAT}$ (α_2=8 aa) with A’-Γ’	ns (p=0.9997)<br>Tukey	
				A-GEMS$_{JAK/STAT}$ (α_2=8 aa) with A’-Γ<br>vs.	ns (p=0.9905)<br>Tukey	

				A-GEMS$_{JAK/STAT}$ (α_2=8 aa) with A’-Γ’		
				A-GEMS$_{JAK/STAT}$ (α_2=8 aa) with A’-Γ and A’-Γ’<br>vs.<br>A-GEMS$_{JAK/STAT}$ (α_2=8 aa) with no ligand	*** (p<0.0001)<br>Tukey	
				A-GEMS$_{JAK/STAT}$ (α_2=8 aa) with A’-Γ and A’-Γ’<br>vs.<br>A-GEMS$_{JAK/STAT}$ (α_2=8 aa) with A’-Γ	*** (p<0.0001)<br>Tukey	
				A-GEMS$_{JAK/STAT}$ (α_2=8 aa) with A’-Γ and A’-Γ’<br>vs.<br>A-GEMS$_{JAK/STAT}$ (α_2=8 aa) with A’-Γ’	*** (p<0.0001)<br>Tukey	
S27d	A-GEMS$_{JAK/STAT}$ (α_2=8 aa) with A’-A’ (bacterial)<br>vs.<br>A-GEMS$_{JAK/STAT}$ (α_2=8 aa) with A’-A’ (mammalian)<br>vs.<br>A-GEMS$_{JAK/STAT}$ (α_2=8 aa) with A’<br>vs.<br>A-GEMS$_{JAK/STAT}$ (α_2=8 aa) with no ligand	860	***<br>(p<0.0001)	A-GEMS$_{JAK/STAT}$ (α_2=8 aa) with A’-A’ (bacterial)<br>vs.<br>A-GEMS$_{JAK/STAT}$ (α_2=8 aa) with A’-A’ (mammalian)	*** (p<0.0001)<br>Šidák	11
6b	receivers with senders<br>(+ dox)<br>vs.<br>receivers with senders<br>(- dox)<br>vs.<br>receivers with HEK293S GnTi$^-$ TetR<br>(+ dox)	869.2	***<br>(p<0.0001)	receivers with senders<br>(+ dox)<br>vs.<br>receivers with senders<br>(- dox)	*** (p<0.0001)<br>Tukey	8
				receivers with senders<br>(+ dox)<br>vs.<br>receivers with HEK293S GnTi$^-$ TetR<br>(+ dox)	*** (p<0.0001)<br>Tukey	
				receivers with senders<br>(- dox)<br>vs.<br>receivers with HEK293S GnTi$^-$ TetR<br>(+ dox)	ns (p=0.1971)<br>Tukey	
6d	receivers with senders 1 & senders 2<br>vs.	512.2	***<br>(p<0.0001)	receivers with senders 1 & senders 2<br>vs.	*** (p<0.0001)<br>Tukey	11

	receivers with senders 1 & control<br>vs.<br>receivers with senders 2 & control<br>vs.<br>receivers with control			receivers with senders 1 & control		
				receivers with senders 1 & senders 2<br>vs.<br>receivers with senders 2 & control	*** (p<0.0001)<br>Tukey	
				receivers with senders 1 & senders 2<br>vs.<br>receivers with control	*** (p<0.0001)<br>Tukey	
				receivers with senders 1 & control<br>vs.<br>receivers with senders 2 & control	ns (p=0.5490)<br>Tukey	
				receivers with senders 1 & control<br>vs.<br>receivers with control	ns (p=0.3153)<br>Tukey	
				receivers with senders 2 & control<br>vs.<br>receivers with control	* (p=0.0481)<br>Tukey	
7b	IL-10 A-type receivers with ($\boldsymbol{\alpha}_2$=8 aa) with A’-A’<br>vs.<br>IL-10 A-type receivers with ($\boldsymbol{\alpha}_2$=8 aa) with Γ’-Γ’<br>vs.<br>IL-10 A-type receivers with ($\boldsymbol{\alpha}_2$=8 aa) with no ligand	138.3	***<br>(p<0.0001)	IL-10 A-type receivers with ($\boldsymbol{\alpha}_2$=8 aa) with A’-A’<br>vs.<br>IL-10 A-type receivers with ($\boldsymbol{\alpha}_2$=8 aa) with Γ’-Γ’	*** (p<0.0001)<br>Tukey	8
				IL-10 A-type receivers with ($\boldsymbol{\alpha}_2$=8 aa) with A’-A’<br>vs.<br>IL-10 A-type receivers with ($\boldsymbol{\alpha}_2$=8 aa) with no ligand	*** (p<0.0001)<br>Tukey	
				IL-10 A-type receivers with ($\boldsymbol{\alpha}_2$=8 aa) with Γ’-Γ’<br>vs.<br>IL-10 A-type receivers with ($\boldsymbol{\alpha}_2$=8 aa) with no ligand	ns (p=0.9962)<br>Tukey	

Supplementary Table S5: DNA and protein sequences for different modifications on monomeric A’ CC module. Three variants (v) of the linker sequence between the SUMO-tag and monomeric A’ CC module used to engineer ditopic A’-A’ CCs (see Supplementary Figure S6). The differences with respect to the reference sequence (ref.) are indicated in red.

ID	DNA Sequence (5' to 3')	Amino acid sequence (N-term to C-term)
**ref.**	AGTGGTTCATGTAGCGGTAGTGGATCC	SGSCSGSGS
**v_1**	AGTGGTTCAGGTAGCGGTAGTGGATCC	SGSGSGSGS
**v_2**	AGTGGTTCATGTAGCGGTCCTGGATCC	SGSCSGPGS
**v_3**	AGTGGTTCATGTAGCGGTCCTGGATCCGGTAGTGGTCCT<br>GGAAGT	SGSCSGPGSGSGPGS

Supplementary Table S6: P-values, t-values and degrees of freedom for unpaired t-tests. Overview of fold change, p-values, t-values and degrees of freedom (df) for unpaired, two-sided t-tests, indicating the compared groups and corresponding Figure. ns: (non-significant), *p ≤0.05, **p ≤0.01, *** p ≤0.001.

Figure	Compared groups	Fold change	p-value summary	p-value	t-value	df
2d	B-GEMS$_{JAK/STAT}$ with A'-A'<br>vs.<br>B-GEMS$_{JAK/STAT}$ with A'	0.0	ns	0.9467	0.07117	4
2d	A-GEMS$_{JAK/STAT}$ (**α**=0 aa) with A'-A'<br>vs.<br>A-GEMS$_{JAK/STAT}$ (**α**=0 aa) with A'	31.8	***	<0.0001	39.21	4
2d	A-GEMS$_{JAK/STAT}$ (**α_1**=4 aa) with A'-A'<br>vs.<br>A-GEMS$_{JAK/STAT}$ (**α_1**=4 aa) with A'	103.6	ns	0.529	2.721	4
2d	A-GEMS$_{JAK/STAT}$ (**α_2**=8 aa) with A'-A'<br>vs.<br>A-GEMS$_{JAK/STAT}$ (**α_2**=8 aa) with A'	47.0	*	0.0113	4.446	4
2d	A-GEMS$_{JAK/STAT}$ (**α_3**=27 aa) with A'-A'<br>vs.<br>A-GEMS$_{JAK/STAT}$ (**α_3**=27 aa) with A'	15.7	***	<0.0001	29.71	4
S7	A-GEMS$_{JAK/STAT}$ with A'-GEMS$_{JAK/STAT}$ (**α_2**=8 aa)<br>vs.<br>A-GEMS$_{JAK/STAT}$ (**α_2**=8 aa) with A'-A'	-	**	0.0084	4.838	4
4b	A-GEMS$_{PLCG}$ (**α_2**=8 aa) with A'-A'<br>vs.<br>A-GEMS$_{PLCG}$ (**α_2**=8 aa) with no ligand	7.6	***	0.0002	4.838	4
S20c	A-GEMS$_{JAK/STAT}$ (**α_2**=8 aa) with cy3-A'- A'<br>vs.<br>A-, B-, Γ-GEMS$_{JAK/STAT}$ (**α_2**=8 aa) with Cy3-A'- A'	-	***	0.0005	3.640	70
S21c	B-GEMS$_{JAK/STAT}$ (**α_2**=8 aa) with cy5-B'- B'<br>vs.<br>A-, B-, Γ-GEMS$_{JAK/STAT}$ (**α_2**=8 aa) with Cy5-B'- B'	-	***	<0.0001	7.771	70
S22c	Γ-GEMS$_{JAK/STAT}$ (**α_2**=8 aa) with AF 488-B'- B'<br>vs.<br>A-, B-, Γ-GEMS$_{JAK/STAT}$ (**α_2**=8 aa) with AF 488-Γ'- Γ'	-	***	<0.0001	7.414	70
S30	A-GEMS$_{JAK/STAT}$ (**α_2**=8 aa) with A'-A'<br>vs.	14.9	***	<0.0001	23.98	4

	A-GEMS$_{JAK/STAT}$ (**α_2**=8 aa) with Senders					

Supplementary Table S7: DNA and protein sequences for linker l_x spanning two CCs for expression of dipeptide ligand. The four different linkers (l_x) used in this work to span two CC modules (see Supplementary Figure S5b). For linker-length optimization (Figure 3), linkers l_1, l_2, l_3[5,6] were utilized. For ligands A'-Γ and A'-Γ' used to construct AND gate logic, linker l_4 was used to span the two unique CC modules (Figure 4b,d).

ID (l_x)	DNA Sequence (5' to 3')	Amino acid sequence (N-term to C-term)
**l_1**	AGTGGATCCTCGGGATCATCAGGATCCTCTGGGAGCTCAGGATCCTCGGGGAGCTCTGGATCCAGCGGTTCAAGCGGATCCTCAGGGAGCTCTGGATCCAGTGGTAGTTCGGGATCCAGCGGATCTAGTGGATCCTCTGGTAGTTCGGGATCCAGTGGTAGCCCGCGGATGGTGAGCAAGGGC	SGSSGSSGSSGSSGSSGSSGSSGSSGSSGSSGSSGSSGSSGSSGSSGSSGSSGSPRMVSKG
**l_2**	TGGGAATTTCATCACGGGGGTTCGGGTGGTTCAGGCGGCTCAGGAGGCTCCGGGGGTTCCGGAGGGAGCGGTGCTGAAGCCGCAGCCAAGGAAGCAGCAGCTAAAGAGGCCGCTGCGAAGGAAGCTGCCGCAAAGGAGGCGGCGGCGAAAGAGGCGGCAGCAAAAGCCGGATCTGGTGGCAGTGGTGGCTCCGGCGGGTCAGGTGGCAGCGGGGGATCAGGAG	WEFHHGGSGGSGGSGGSGGSGGSGAEAAAKEAAAKEAAAKEAAAKEAAAKEAAAKAGSGGSGGSGGSGGSGGSG
**l_3**	TGGGAATTTCATCACGGGGGTTCGGGTGGTTCAGGCGGCTCAGGAGGCTCCGGGGGTTCCGGAGGGAGCGGTGCTGAAGCCGCAGCCAAGGAAGCAGCAGCTAAAGAGGCCGCTGCGAAGGAAGCTGCCGCAAAGGAGGCGGCGGCGAAAGAGGCGGCAGCAAAAGCCGGATCTGGTGGCAGTGGTGGCTCCGGTGGGTCAGGTGGCAGCGGGGGATCAGGAGCTGAGGCAGCCGCCAAAGAGGCTGCGGCCAAGGAGGCCGCCGCTAAAGAAGCCGCGGCAAAAGAGGCAGCGGCAAAGGAAGCGGCTGCGAAAGCCGGAAGTGGTGGGTCGGGCGGCTCCGGTGGCTCAGGCGGTCTGTGG	WEFHHGGSGGSGGSGGSGGSGGSGAEAAAKEAAAKEAAAKEAAAKEAAAKEAAAKAGSGGSGGSGGSGGSGGSGAEAAAKEAAAKEAAAKEAAAKEAAAKEAAAKAGSGGSGGSGGSGGLW
**l_4**	TGGGAATTTCATCACGGGGGTTCGGGTGGTTCAGGCGGC	WEFHHGGSGGSGG

Supplementary Table S8: Engineered plasmids used in the present study. Overview of engineered plasmids, including plasmid backbone and gene insert.

Plasmid ID	Plasmid Backbone	Gene insert	Figure-reference
pET28a(+)-SUMO-A' (Addgene plasmid #209115)	pET28a(+)-P_{T7}	SUMO-A' CC	Figure 2, Supplementary Figure S4, Supplementary Figure S7, Supplementary Figure S27
pET28a(+)-SUMO-A'(v_1)	pET28a(+)-P_{T7}	SUMO-A'.C4G CC (v_1)	Supplementary Figure S6
pET28a(+)-SUMO-A'(v_2)	pET28a(+)-P_{T7}	SUMO-A'.S7P CC (v_2)	Supplementary Figure S6
pET28a(+)-SUMO-A'(v_3)	pET28a(+)-P_{T7}	SUMO-A'.S7P.Link CC (v_3)	Supplementary Figure S6
pET28a(+)-SUMO-A'-l_1-A' (Addgene plasmid #209116)	pET28a(+)-P_{T7}	SUMO-A' CC-l_1-A' CC	Figure 3, Supplementary Figure S8
pET28a(+)-SUMO-A'-l_2-A' (Addgene plasmid #209117)	pET28a(+)-P_{T7}	SUMO-A' CC-l_2-A' CC	Figure 3, Figure 4, Figure 5, Figure 7, Supplementary Figure S8, Supplementary Figure S9, Supplementary Figure S10, Supplementary Figure S11, Supplementary Figure S12, Supplementary Figure S16, Supplementary Figure S19,

			Supplementary Figure S20, Supplementary Figure S27, Supplementary Figure S29, Supplementary Figure S30, Supplementary Figure S34
pET28a(+)-SUMO-A’-l_3-A’ (Addgene plasmid #209118)	pET28a(+)-P_{T7}	SUMO-A’ CC-l_3-A’ CC	Figure 3, Supplementary Figure S8
pET28a(+)-SUMO-B’-l_2-B’ (Addgene plasmid #209119)	pET28a(+)-P_{T7}	SUMO-B’ CC-l_2-B’ CC	Figure 5, Supplementary Figure S10, Supplementary Figure S13, Supplementary Figure S14, Supplementary Figure S16, Supplementary Figure S19, Supplementary Figure S21
pET28a(+)-SUMO-Γ’-l_2-Γ’ (Addgene plasmid #209120)	pET28a(+)-P_{T7}	SUMO-Γ’ CC-l_2-Γ’ CC	Figure 5, Figure 7, Supplementary Figure S10, Supplementary Figure S13, Supplementary Figure S15, Supplementary Figure S16, Supplementary Figure S19, Supplementary Figure S22
pET28a(+)-SUMO-A’-l_2-B’ (Addgene plasmid #209121)	pET28a(+)-P_{T7}	SUMO-A’ CC-l_2-B’ CC	Figure 5, Supplementary Figure S17, Supplementary S18
pET28a(+)-SUMO-A’-l_2-Γ’ (Addgene plasmid #209122)	pET28a(+)-P_{T7}	SUMO-A’ CC-l_2-Γ’ CC	Figure 4, Supplementary Figure S17, Supplementary S18
pET28a(+)-SUMO-A’-l_4-Γ (Addgene plasmid #209123)	pET28a(+)-P_{T7}	SUMO-A’ CC-l_4-Γ CC	Figure 5, Supplementary Figure S23, Supplementary Figure S24, Supplementary Figure S25
pET28a(+)-SUMO-A’-l_4-Γ’ (Addgene plasmid #209124)	pET28a(+)-P_{T7}	SUMO-A’ CC-l_4-Γ’ CC	Figure 5, Supplementary Figure S23, Supplementary Figure S24, Supplementary Figure S25
pLeo-A-$GEMS_{JAK/STAT}$ (Addgene plasmid #209109)	pLeo619-P_{SV40}	A CC-$GEMS_{JAK/STAT}$	Figure 1, Figure 2, Supplementary Figure S6
pLeo-A-α_1-$GEMS_{JAK/STAT}$	pLeo619-P_{SV40}	A CC-α_1-$GEMS_{JAK/STAT}$	Figure 1, Figure 2, Supplementary Figure S6
pLeo-A-α_2-$GEMS_{JAK/STAT}$ (Addgene plasmid #209110)	pLeo619-P_{SV40}	A CC-α_2-$GEMS_{JAK/STAT}$	Figure 1, Figure 2, Figure 3, Figure 5, Figure 6, Figure 7, Supplementary Figure S6, Supplementary Figure S7, Supplementary Figure S14, Supplementary Figure S15, Supplementary Figure S16, Supplementary Figure S17, Supplementary Figure S18, Supplementary Figure S19, Supplementary Figure S20, Supplementary Figure S21, Supplementary Figure S22, Supplementary Figure S25, Supplementary Figure S27, Supplementary Figure S30

pLeo-A-α_3-GEMS$_{JAK/STAT}$ (Addgene plasmid #209111)	pLeo619-P$_{SV40}$	A CC-α_3-GEMS$_{JAK/STAT}$	Figure 1, Figure 2, Supplementary Figure S6
pLeo-A'-GEMS$_{JAK/STAT}$	pLeo619-P$_{SV40}$	A'-CC-GEMS$_{JAK/STAT}$	Figure 1
pLeo-A'-α_1-GEMS$_{JAK/STAT}$	pLeo619-P$_{SV40}$	A' CC-α_1-GEMS$_{JAK/STAT}$	Figure 1
pLeo-A'-α_2-GEMS$_{JAK/STAT}$	pLeo619-P$_{SV40}$	A' CC-α_2-GEMS$_{JAK/STAT}$	Figure 1, Supplementary Figure S7
pLeo-A'-α_3-GEMS$_{JAK/STAT}$	pLeo619-P$_{SV40}$	A' CC-α_3-GEMS$_{JAK/STAT}$	Figure 1
pLeo-B-α_2-GEMS$_{JAK/STAT}$ (Addgene plasmid #209112)	pLeo619-P$_{SV40}$	B CC-α_2-GEMS$_{JAK/STAT}$	Figure 1, Figure 2, Figure 5, Supplementary Figure S6, Supplementary Figure S11, Supplementary Figure S14, Supplementary S15, Supplementary S16, Supplementary S17, Supplementary Figure S18, Supplementary Figure S19, Supplementary Figure S20, Supplementary Figure S21, Supplementary Figure S22
pLeo-Γ-α_2-GEMS$_{JAK/STAT}$ (Addgene plasmid #209113)	pLeo619-P$_{SV40}$	Γ CC-α_2-GEMS$_{JAK/STAT}$	Figure 5, Supplementary Figure S11, Supplementary Figure S14, Supplementary Figure S15, Supplementary Figure S17, Supplementary Figure S18, Supplementary Figure S19, Supplementary Figure S20, Supplementary Figure S21, Supplementary Figure S22
pLeo-A-α_2-GEMS$_{PLCG}$ (Addgene plasmid #209114)	pLeo619-P$_{SV40}$	A CC-α_2-GEMS$_{PLCG}$	Figure 4, Supplementary Figure S12
pHR-EGFPligand	pHR-P$_{SFFV}$	EGFP-PDGFR	Figure 1, Supplementary Figure S3 (pHR_EGFPligand was a gift from Wendell Lim (Addgene plasmid # 79129 ; http://n2t.net/addgene:79129 ; RRID:Addgene_79129))[7]
pHR-SUMO-A'-l$_2$-A'-IRES-EmGFP (Addgene plasmid #209125)	pHR-P$_{CMV-TetO2}$	SUMO-A' CC-l$_2$-A' CC-IRES-EmGFP	Figure 6, Supplementary Figure S27, Supplementary Figure S28, Supplementary S29, Supplementary S30
pHR-SUMO-A'-l$_4$-Γ-IRES-EmGFP (Addgene plasmid #209126)	pHR-P$_{CMV-TetO2}$	SUMO-A' CC-l$_4$-Γ CC-IRES-EmGFP	Figure 6, Supplementary Figure S32, Supplementary Figure 34
pHR-SUMO-A'-l$_4$-Γ'-IRES-EmGFP (Addgene plasmid #209127)	pHR-P$_{CMV-TetO2}$	SUMO-A' CC-l$_4$-Γ' CC-IRES-EmGFP	Figure 6, Supplementary Figure S33, Supplementary Figure 34
pLS13-IL-10 (Addgene plasmid #209128)	pLS13$_{STAT3-RE}$	IL-10	Figure 7

Supplementary Figures

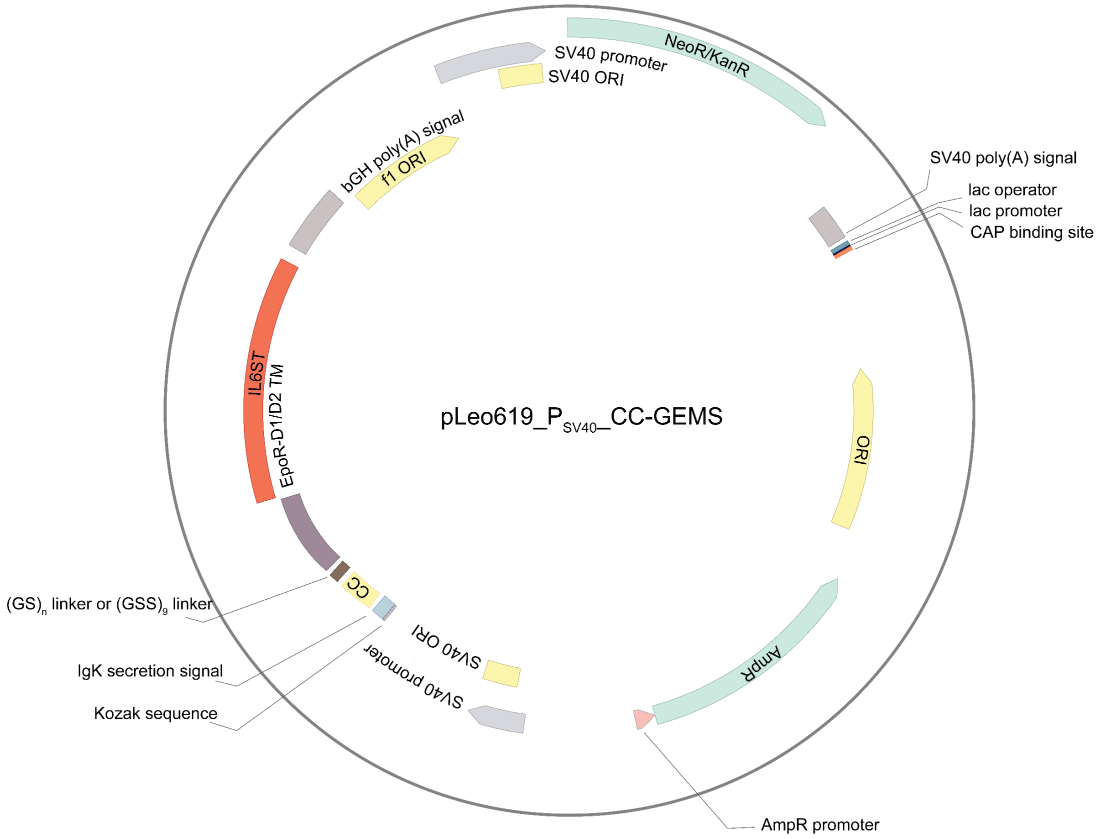

Supplementary Figure S1: Plasmid map of pLeo619 expression vector for transient mammalian expression of the CC functionalized GEMS receptor. The CC-GEMS receptor consists of a selected CC sequence (see Supplementary Table S1), a linker α_x composed of GS or GGS repeats consisting of zero, four, eight or 27 aa length ((GS)$_n$ linker, where n=0, 2, 4 or (GSS)$_9$ linker; Supplementary Table S2) and the EpoR transmembrane domain (EpoR-D1/D2 TM) fused to the intracellular signal transduction domains of IL-6RB (interleukin 6 receptor B; IL6ST), collectively referred to as the GEMS receptor. The construct is under the control of a mammalian SV40 promoter. Ampicillin resistance gene (AmpR) is available for bacterial selection downstream of an AmpR promoter. ORI, origin of replication; CAP, catabolite binding protein; NeoR/KanR; neomycin/kanamycin resistance; bGH, bovine growth hormone.

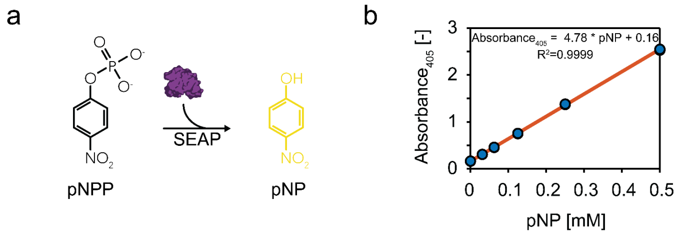

Supplementary Figure S2: Calibration curve to determine pNPP conversion by SEAP. a SEAP catalyses the hydrolysis of the chromogenic substrate p-Nitrophenylphosphate (pNPP) to p-Nitrophenol (pNP), the absorbance of which can be measured at 405 nm. **b** To convert absorption units to amount of pNP, we titrated known concentrations of pNP (0 mM, 0.03125 mM, 0.0625 mM, 0.125 mM, 0.25 mM and 0.5 mM), measured

absorbance units (405 nm at 25ºC) and fitted the data points using a linear regression curve. $Absorbance_{405} = 4.78 * [pNP] + 0.16$, R^2=0.9999. Individual data points represent technical replicates (n=3).

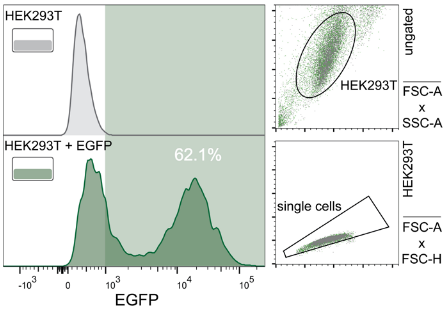

Supplementary Figure S3: Quantification of transfection efficiency. Flow cytometry analysis of EGFP transfected HEK293T cells (green; see Methods) and control (un-transfected HEK293T; grey), showing the gating strategy on the right (dot plots; FSC-A x SSC-A and FSC-A x FSC-H). From the transfected population, 62.1% (green shaded area) of HEK293T cells express EGFP.

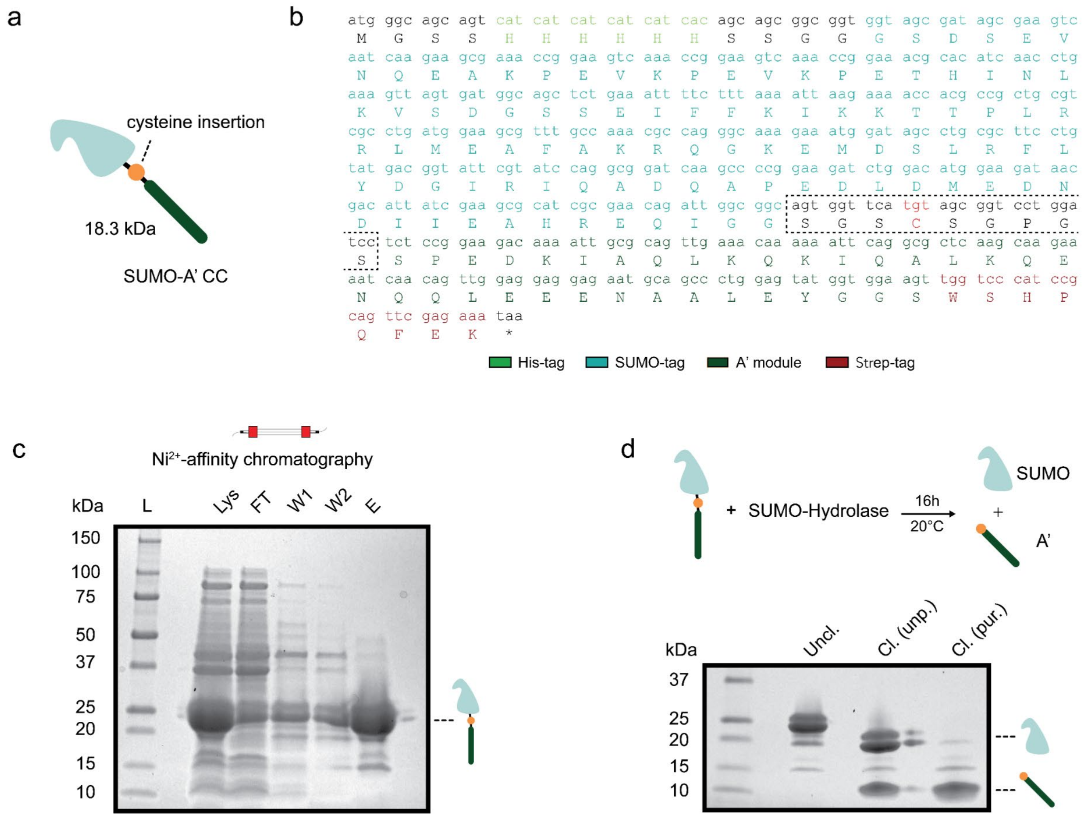

Supplementary Figure S4: Expression of monomeric CC peptide A'. a Schematic representation of SUMO-A' CC fusion protein (expected size: 18.3 kDa). A cleavable SUMO-tag is N-terminally fused to monomeric CC peptide A', bearing a single cysteine. The SUMO tag is fused to a His-tag at the N-terminus and the A' CC is fused to a Strep-tag at the C-terminus (see Methods and Figure 2b). **b** DNA and protein sequence of the SUMO-A' CC fusion protein. The single-letter amino acid code is shown in uppercase below the corresponding DNA sequence (lowercase). The His-tag is shown in light green, the SUMO-tag in light blue, the cysteine in red, the A' CC module in dark green and the Strep-tag in red. **c** SUMO-A' CC fusion protein was expressed in *E. coli* (see Methods, Supplementary Table S1 and supplementary Figure S5a) and purification with Ni^{2+}-affinity chromatography

afforded pure SUMO-A’ CC fusion protein, as shown by SDS-PAGE analysis. L: molecular weight ladder, Lys: cell lysate, FT: flow through, W1: wash fraction 1, W2: wash fraction 2, E: elution fraction. **d** SUMO-A’ CC fusion protein is incubated with SUMO-hydrolase for 16 h, at 20°C (upper panel), resulting in the cleavage of the SUMO tag from the A’ CC (lower panel, Cl. (unp.)). Following purification with Ni^{2+} affinity chromatography, CC peptide A’ was recovered (Cl. (pur.)).

a b

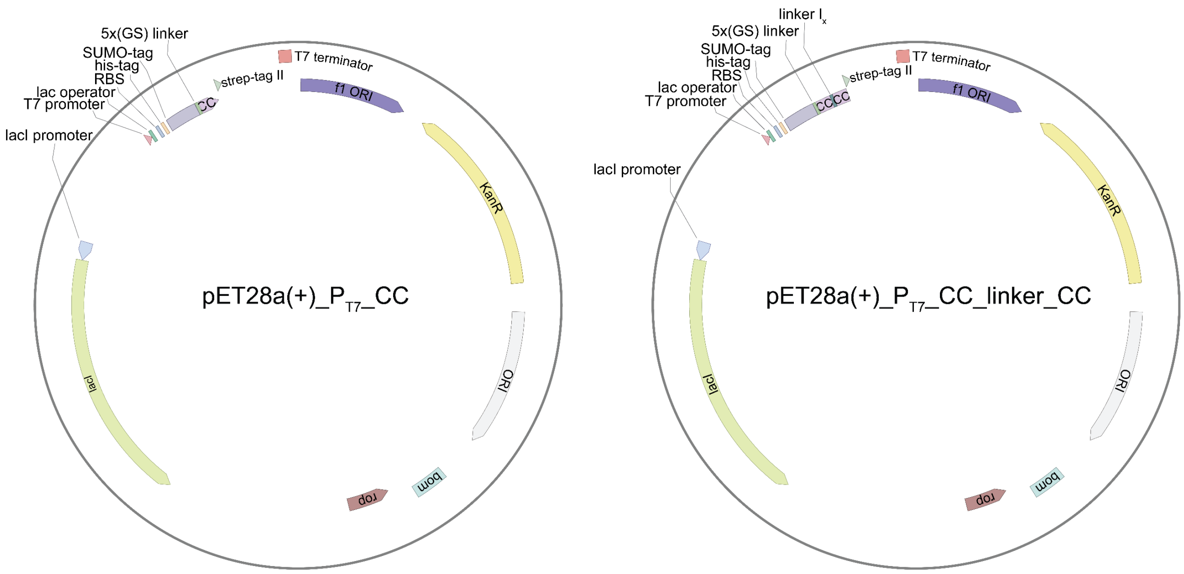

Supplementary Figure S5: Plasmid maps of pET28a(+) expression vector for expression of the monomeric and dimeric CC ligands. a The selected CC sequence is SUMO-tagged and expressed downstream of a T7 promoter. An N-terminus His-tag and a C-terminus strep-tag are incorporated for subsequent purification. **b** Two unique CC modules are spanned by a linker l_x (see Supplementary Table S7). A His-tag is engineered on the N-terminus of the SUMO-tag and a strep tag on the C-terminus of the CC-linker-CC fusing protein. A kanamycin resistance gene (KanR) is available for bacterial selection. ORI, origin of replication; bom; basis of mobility; rop, repressor of primer; RBS, ribosome binding site.

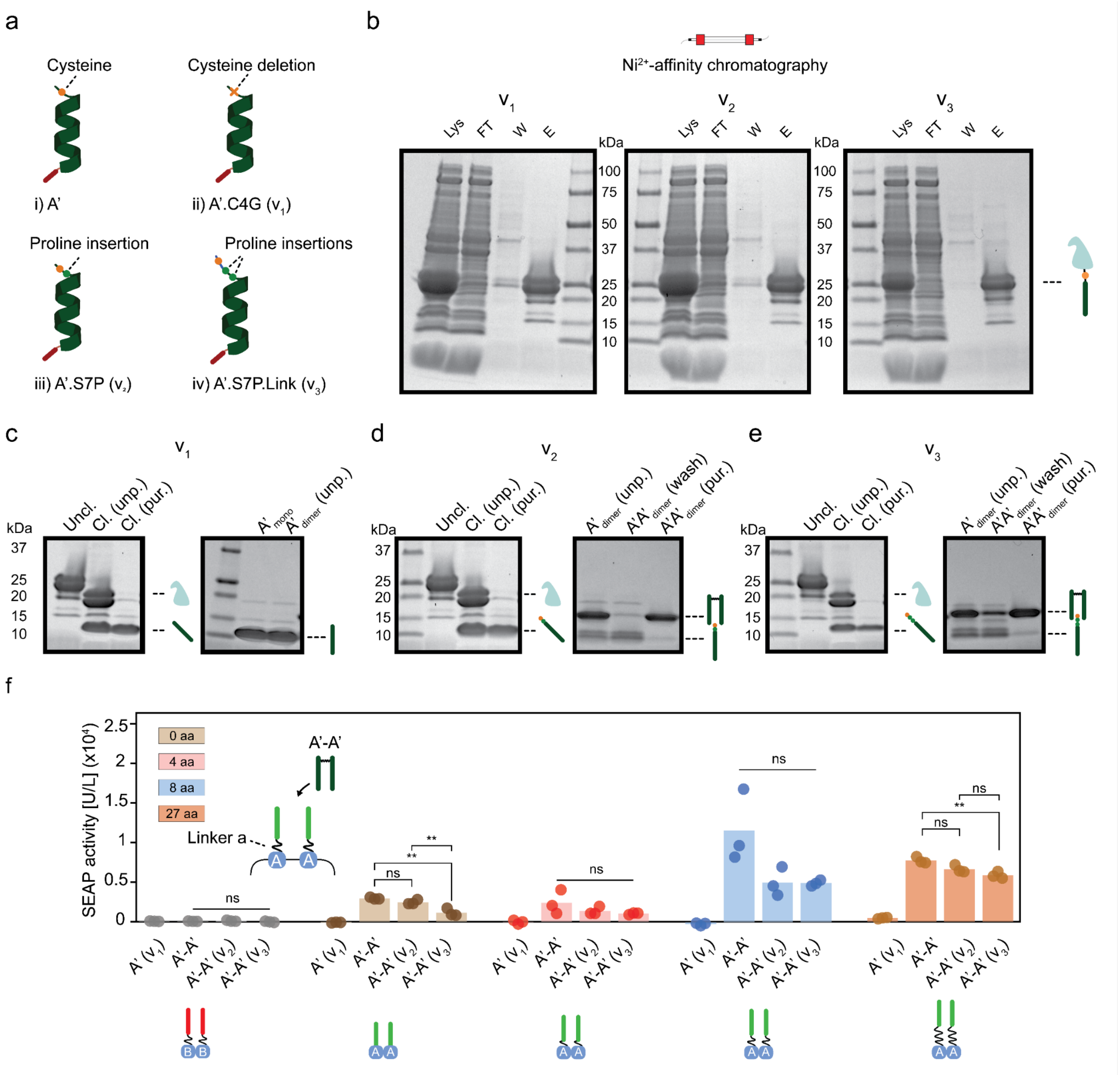

Supplementary Figure S6: Mutant A' CC expression, purification, and subsequent effects on receptor activation. a Schematic representation of strategy to express three variants (v_x) of mutant monomeric A' CCs; i) A': original A' monomer (see Supplementary Figure S4b), ii) A'.C4G (v_1): cysteine deletion, iii) A'.S7P (v_2): mutant with the addition of a proline, iv) A'.S7P.Link (v_3): variant with addition of a small linker (see Supplementary Table S5). **b** SDS-Page analysis of the expression of the variants v_1, v_2 and v_3 following purification with Ni^{2+}-affinity chromatography. Lys: cell lysate, FT: flow through, W: wash fraction, E: elution fraction. **c** SDS-PAGE of v_1 prior to SUMO-cleavage (Uncl.), after (Cl.(Unp.)) and following purification with Ni^{2+}-affinity chromatography (Cl.(pur.)) (left). SDS-PAGE analysis, showing formation of the SUMO-tagged A' peptide for the v_1, v_2 and v_3 variants, following incubation with homobifunctional bismaleimide linker (right; see Methods and Figure 2c). **d** and **e** SDS-PAGE analysis of the engineering of v_2 and v_3 dipeptides as in **c**. Ditopic A'A' ligands were formed only when N-terminal cysteine was present. Anion exchange chromatography is able to remove unreactive A' monomers ($A'A'_{dimer}$(wash)) and recover only ditopic A'A' ligands ($A'A'_{dimer}$(pur.)). **f** SEAP activity [U/L] in HEK293T cells transiently transfected with receptor homodimers (A-type$_{JAK/STAT}$ receptor in light green; or B-type$_{JAK/STAT}$ receptor in red) with varied linkers α_x of zero, four, eight, and 27 aa (GS or GGS repeats). Cells were incubated with either 0.12 µM A'-A' dipeptide mutants (A'-A', A'-A' (v_2) or A'-A' (v_3)) or 0.24 µM of monomeric A'(v1) CC for 48h and SEAP expression was measured to assess receptor activation (see Methods). All mutant ditopic ligands result in reporter expression for cells expressing a receptor bearing a compatible CC (A) for all linkers α_x. Monomeric CCs result in no activation. Additionally, the ditopic ligands fail to activate receptors harbouring a non-cognate CC with an 8 aa linker α_2 (B-type$_{JAK/STAT}$ receptor in red). Bars indicate mean activity; individual data

points represent independent triplicates, performed on the same day. Significance (ANOVA with Tukey multiple comparison correction) is noted above bars (see Supplementary Table S4). ns: p>0.05, *p ≤0.05, **p ≤0.01, ***p ≤0.001.

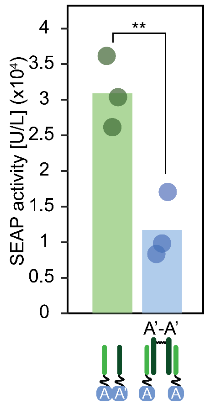

Supplementary Figure S7: Comparison between heterodimeric receptor activation and ligand-induced receptor activation. SEAP activity [U/L] in HEK293T cells transiently transfected with receptor heterodimers (A-type$_{JAK/STAT}$ and A'-type$_{JAK/STAT}$ receptors with α2 in green;) or A-type$_{JAK/STAT}$ receptor with α2, subsequently incubated with 0.12 µM ditopic A'-A' ligand for 48h (in blue) (see Methods). Bars indicate mean activity; individual data points represent independent triplicates, performed on the same day. Significance (unpaired t-test) is noted above bars (see Supplementary Table S6). ns: p>0.05, *p ≤0.05, **p ≤0.01, ***p ≤0.001.

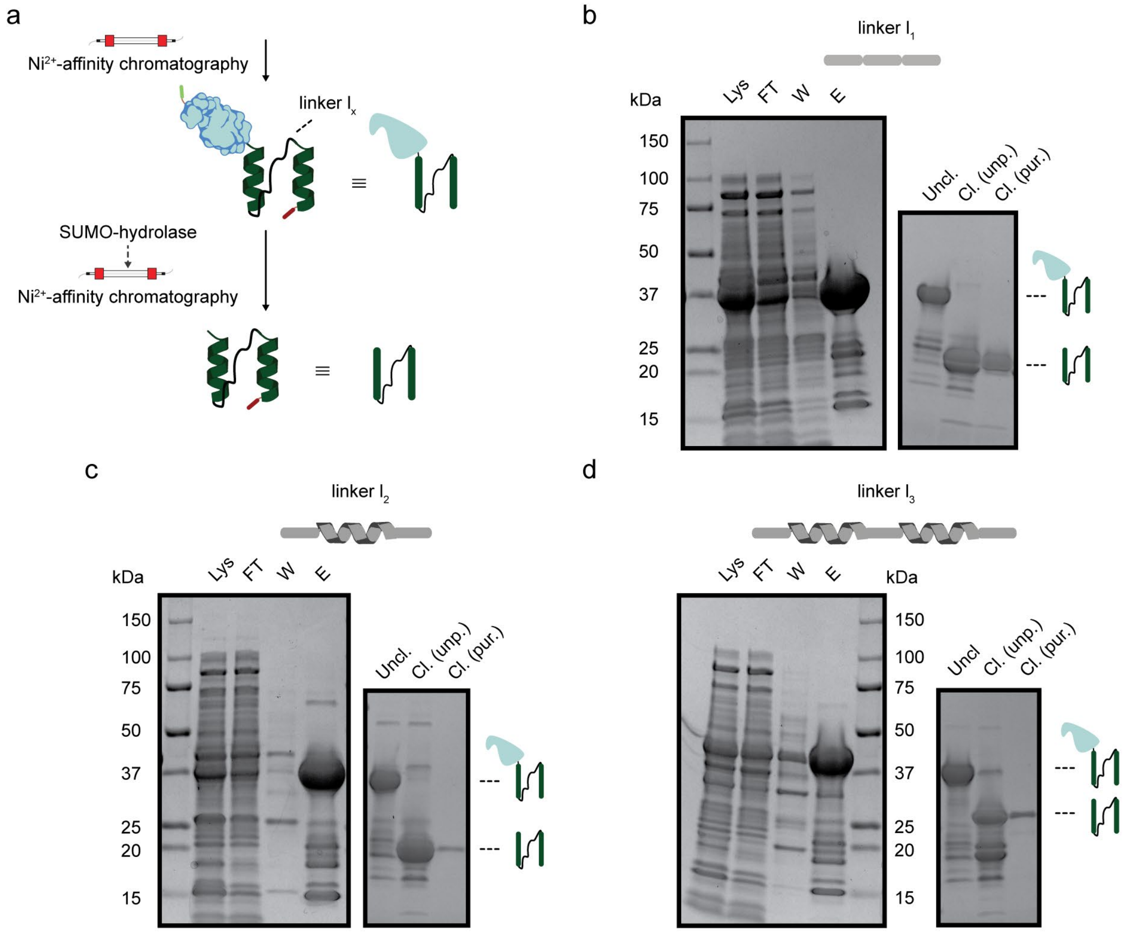

Supplementary Figure S8: Protein linker-based dipeptide A'-A' ligand expression and purification. a SUMO-tagged A'-A' dipeptide carrying protein linker l_x is purified using Ni^{2+}-affinity chromatography. The SUMO tag is

removed and A’-A’ dipeptide is purified and recovered using an additional Ni^{2+}-affinity chromatography step (see Methods). **b, c** and **d** SDS-PAGE analysis, showing the expression of the engineered A’-A’ dipeptides carrying linker l_1 (**b**), l_2 (**c**) and l_3 (**d**), following purification with Ni^{2+}-affinity chromatography (left panels). Lys: cell lysate, FT: flow through, W: wash fraction, E: elution fraction. Right: Un-cleaved A’-A’ CC dipeptide (Uncl.) was incubated with SUMO-hydrolase (Cl. (unp.)) to cleave of the SUMO-tag (see Methods). An additional step of Ni^{2+}-affinity chromatography afforded pure A’-A’ CC dipeptide (Cl. (pur.)).

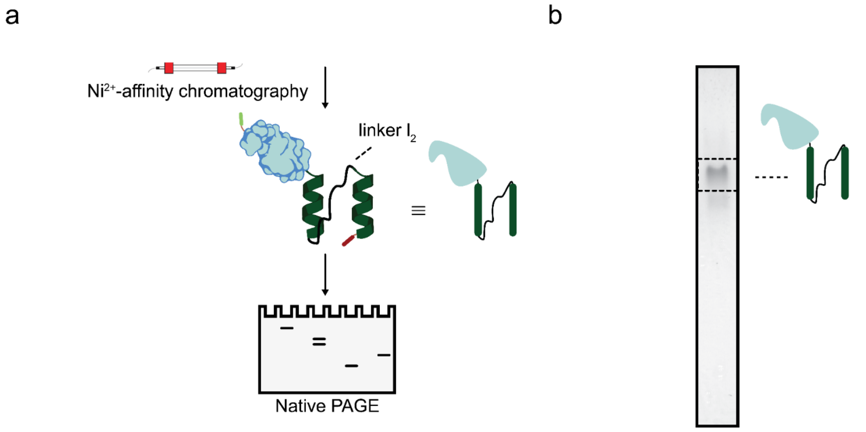

Supplementary Figure S9: Native PAGE of A’-A’ dipeptide. a SUMO-tagged A’-A’ dipeptide with protein linker $\mathbf{l_2}$ is purified using Ni^{2+}-affinity chromatography and subsequently run on a Native-PAGE gel to assess ligand aggregation and oligomer formation (see Methods). **b** Native-PAGE analysis of purified A’-A’ ligand showing one distinct band.

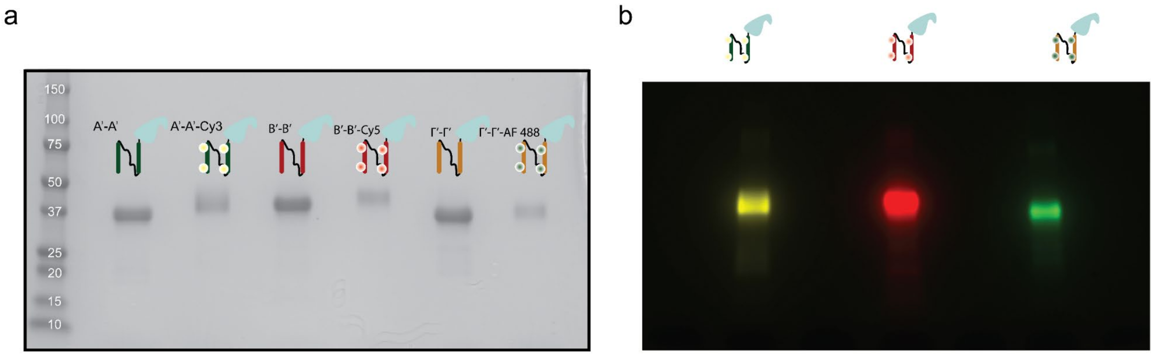

Supplementary Figure S10: CC dipeptide labelling. a SDS-PAGE gel showing labelled and unlabelled, SUMO-tagged A’-A’, B’-B’, and Γ’-Γ’ dipeptide ligands (see Methods). **b** Fluorescence image of SDS-PAGE gel, showing Cy3-labelled A’-A’ in yellow, Cy5-labelled B’-B’ ligand in red, and Alexa 488-labelled Γ’-Γ’ ligand in green. Cy3; excitation: 535 nm, emission: 570 nm. Cy5; excitation: 635 nm, emission: 662 nm. AF 488; excitation: 460 nm, emission: 510 nm.

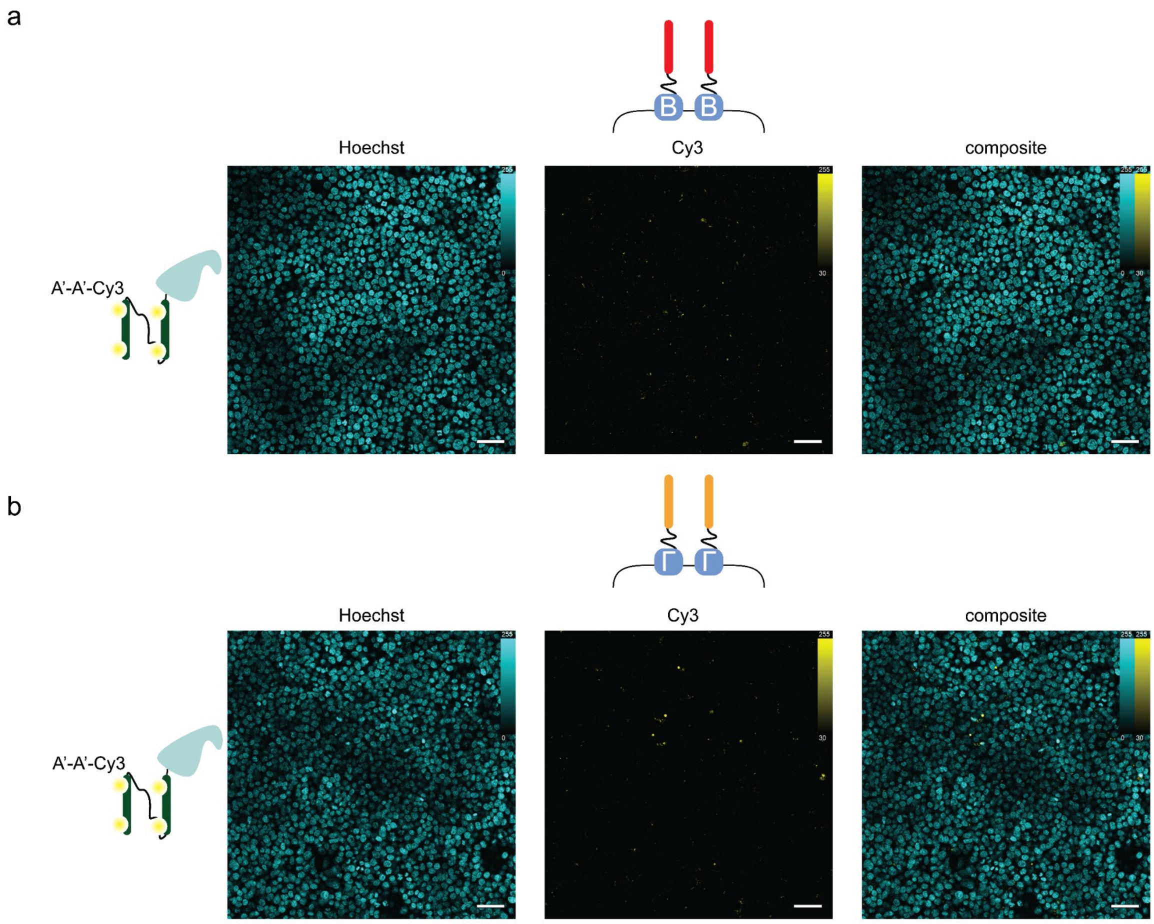

Supplementary Figure S11: Confocal microscopy of B-type and Γ-type cells incubated with Cy3-labelled A’-A’ ligand. Confocal micrograph images of HEK293T cells expressing **a** B-type or **b** Γ-type receptor following incubation with Cy3-labelled SUMO-tagged A’-A’ dipeptide. Cells were stained with Hoechst stain (blue). Cy3 is depicted in yellow. Scale bar (50 µm) is shown on the bottom-right of the image and intensity bar on top-right. Cy3 excitation: 553 nm, emission: 570-620 nm. Hoechst excitation: 405 nm, emission: 410-450 nm.

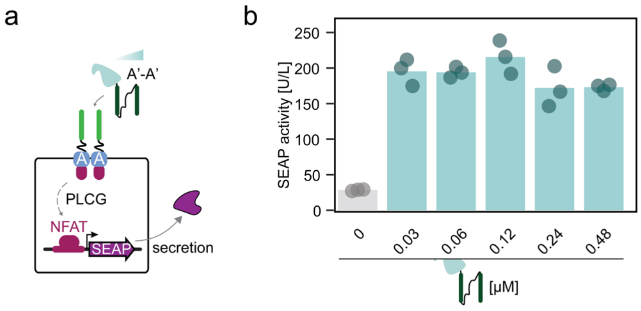

Supplementary Figure S12: A’-A’ ligand titration on cells expressing A-type$_{PLCG}$ receptor. a Schematic overview of the reporter system to monitor CC-GEMS receptor activation using phospholipase C gamma (PLCG) intracellular signalling. HEK293T cells were transfected with A-type$_{PLCG}$ receptors with linker α_2 and a SEAP reporter gene, engineered to be responsive to PLCG activation, through an NFAT (nuclear factor of activated T cells)-responsive minimal promoter (Methods). Cells were subsequently treated with a range of concentrations of SUMO-tagged A’-A’ with I_2. **b** SEAP activity [U/L], measured 48 hours following transfection for cells incubated with 0, 0.03, 0.06, 0.12, 0.24, and 0.48 µM SUMO-tagged A’-A’ with I_2 (see Methods). Bars indicate mean activity; individual data points represent independent triplicates, performed on the same day.

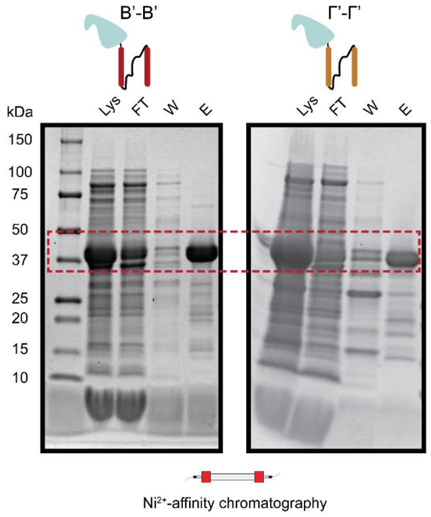

Supplementary Figure S13: B'-B' and Γ'-Γ' CC ligand expression and purification. SDS-PAGE analysis, showing the expression of the engineered B'-B' (left panel) and Γ'-Γ' (right panel) CC dipeptides carrying linker l_2, following purification with Ni^{2+}-affinity chromatography. Lys: cell lysate, FT: flow through, W: wash fraction, E: elution fraction. Red inset highlights the position of the ligand on gel.

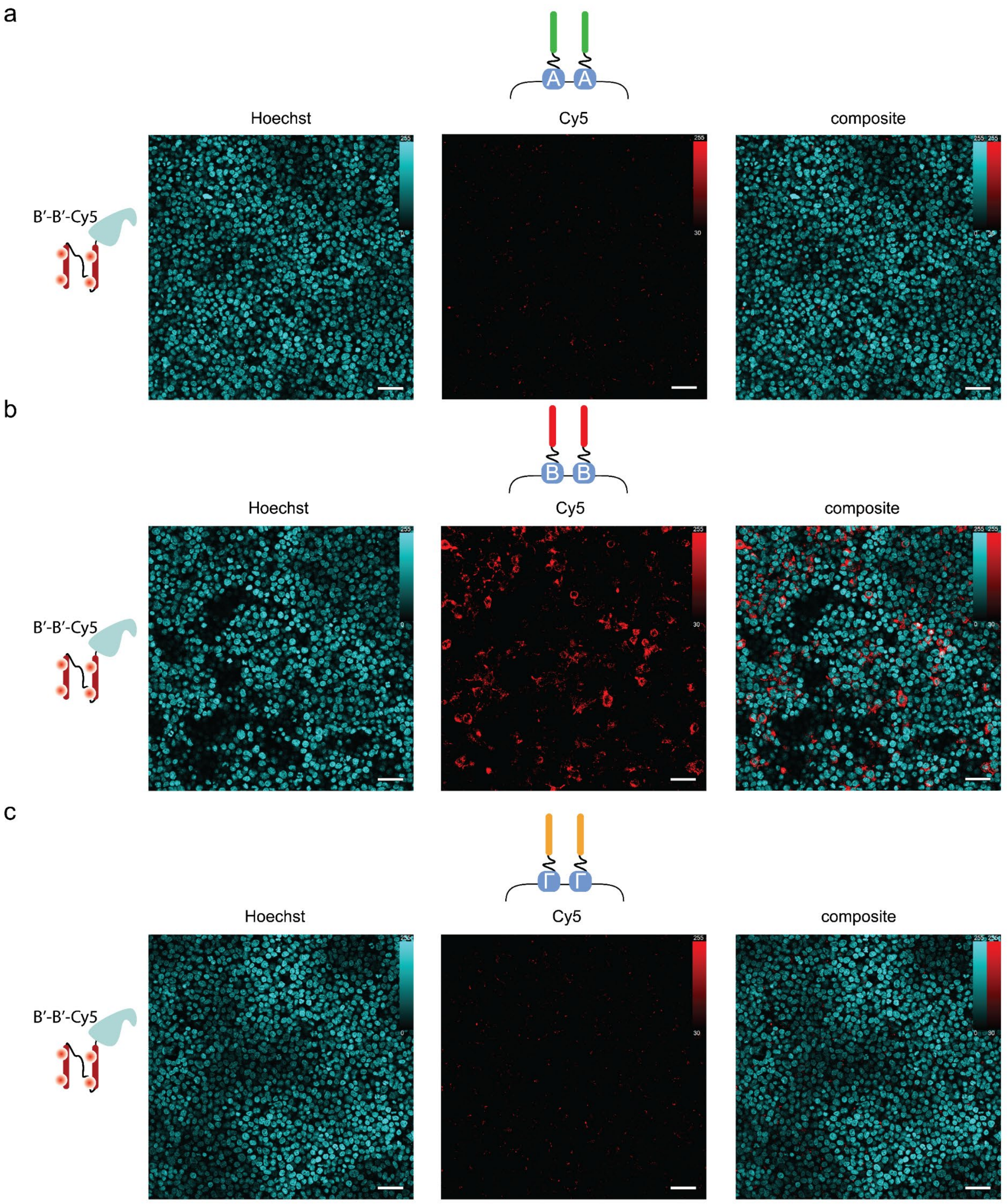

Supplementary Figure S14: Confocal microscopy of A-type$_{\text{JAK/STAT}}$, B-type$_{\text{JAK/STAT}}$ and Γ-type$_{\text{JAK/STAT}}$ cells incubated with Cy5-labelled B'-B' ligand. Confocal micrograph images of HEK293T cells expressing either **a** A-type$_{\text{JAK/STAT}}$, **b** B-type$_{\text{JAK/STAT}}$ or **c** Γ-type$_{\text{JAK/STAT}}$ receptor following incubation with Cy5-labelled SUMO-tagged B'-B' dipeptide. Cells were stained with Hoechst stain (blue). Cy5 is depicted in red. Scale bar (50 µm) is shown on the bottom-right of the image and intensity bar on top-right. Cy5 excitation: 647 nm, emission: 655-705 nm. Hoechst excitation: 405 nm, emission: 410-450 nm.

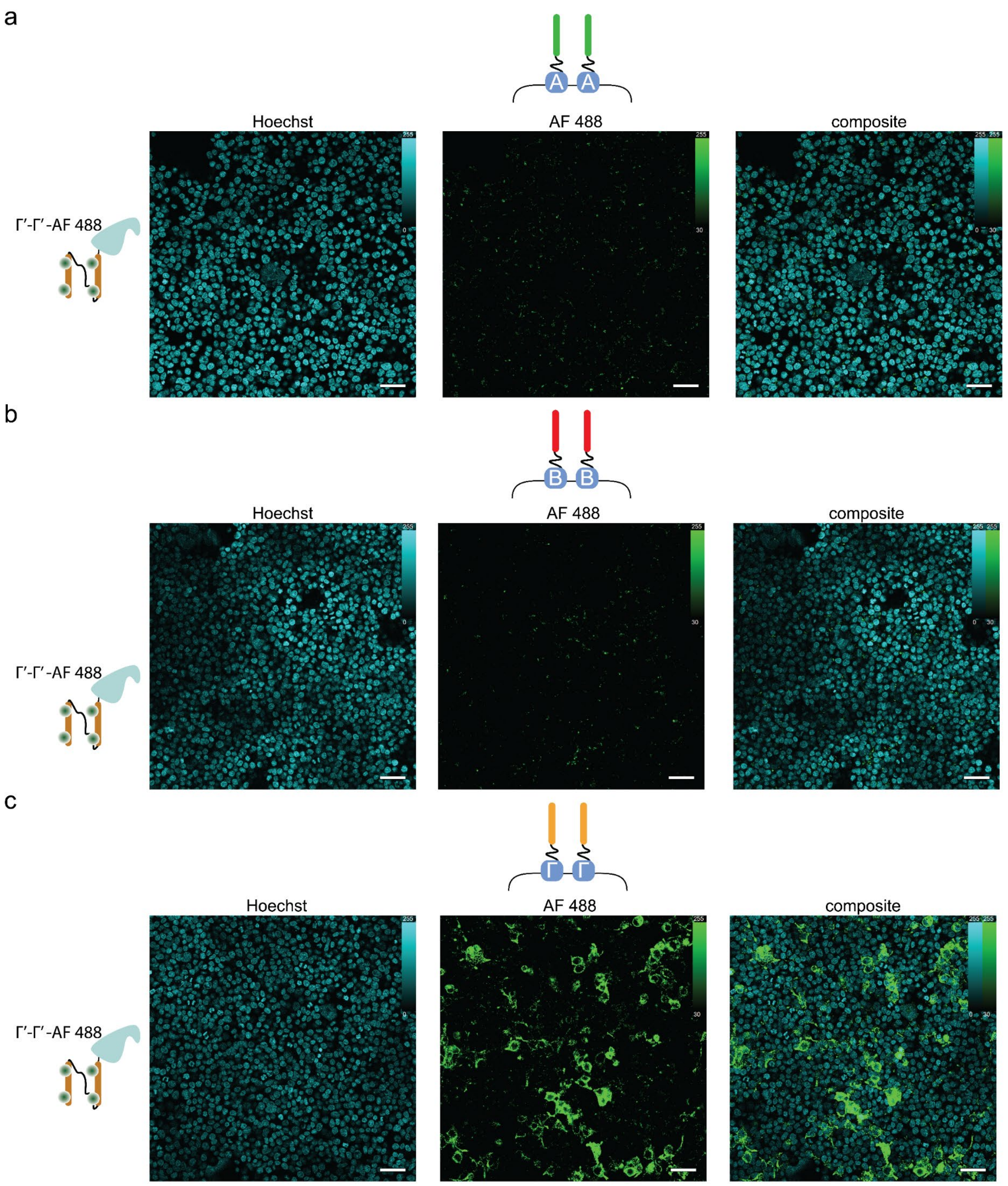

Supplementary Figure S15: Confocal microscopy of A-type$_{JAK/STAT}$, B-type$_{JAK/STAT}$ and Γ-type$_{JAK/STAT}$ cells incubated with AF 488-labelled Γ'-Γ' ligand. Confocal micrograph images of HEK293T cells expressing either **a** A-type$_{JAK/STAT}$, **b** B-type$_{JAK/STAT}$ or **c** Γ-type$_{JAK/STAT}$ receptor following incubation with AF 488-labelled SUMO-tagged Γ'-Γ' dipeptide. Cells were stained with Hoechst stain (blue). AF 488 is depicted in green. Scale bar (50 µm) is shown on the bottom-right of the image and intensity bar on top-right. AF 488 excitation: 488 nm, emission: 500-550 nm. Hoechst excitation: 405 nm, emission: 410-450 nm.

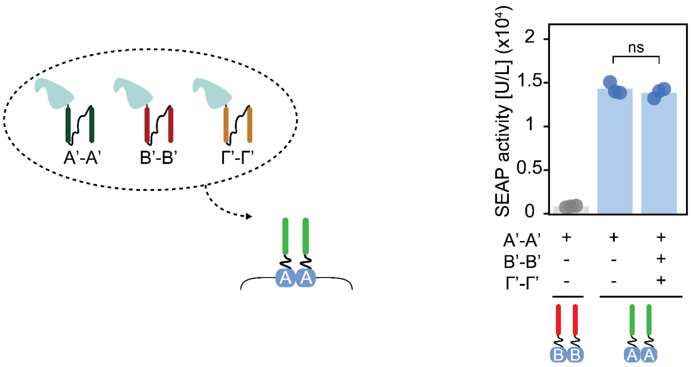

Supplementary Figure S16: Alternative non-cognate ligands do not inhibit activation in cells caused by cognate receptor-ligand pairing. 0.12 µM SUMO-tagged A'-A', B'-B' and Γ'-Γ' dipeptides with protein linker l_2 were incubated with HEK293T cells transiently transfected to express A-type$_{JAK/STAT}$ (green) or B-type$_{JAK/STAT}$ (red) receptor with linker α_2 (8 aa, GS repeats). Following transfection, cells were incubated with ligand for 48 hours and SEAP activity was measured (see Methods). Right panel shows the SEAP activity [U/L] for different receptor-ligand combination pairs. Cells expressing the A-type$_{JAK/STAT}$ receptor are activated by the presence of A'-A' ligand. SEAP activity is not inhibited by the presence of B'-B' and Γ'-Γ' dipeptide ligands. Bars indicate mean activity; individual data points represent independent triplicates, performed on the same day. Significance (ANOVA with Šidák test) is noted above bars (Supplementary Table S4). ns: p>0.05, *p ≤0.05, **p ≤0.01, ***p ≤0.001.

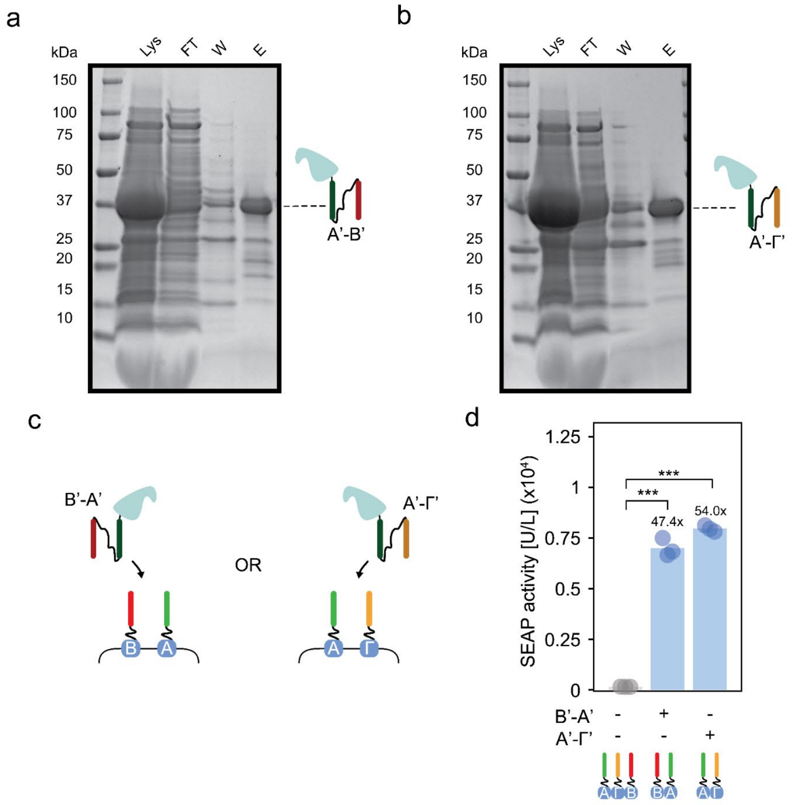

Supplementary Figure S17: B'-A' and A'-Γ' dipeptide ligands activate cells transfected with B-A- and A-Γ-type$_{JAK/STAT}$ receptors. SDS-PAGE analysis, showing the expression of the engineered B'-A' (**a**) and A'-Γ' (**b**) CC dipeptides carrying linker l_2, following purification with Ni^{2+}-affinity chromatography (Methods). Lys: cell lysate, FT: flow through, W: wash fraction, E: elution fraction. **c** HEK293T cells transiently transfected with either B- and A- type$_{JAK/STAT}$ or A- and Γ-type$_{JAK/STAT}$ receptors, with 8 aa linker α_2 (GS repeats) were incubated with 0.5 µM cognate SUMO-tagged dipeptide ligand B'-A' and A'-Γ' with linker l_2 respectively for 48 hours and SEAP activity was measured (see Methods). **d** Following incubation with ligand, normalized SEAP activity was determined;

which shows receptor activation for cognate ligand-receptor pairs (B'-A' ligand and B- and A-type$_{JAK/STAT}$ receptor; and A'-Γ' ligand and A- and Γ-type$_{JAK/STAT}$ receptors), at similar levels. The absence of ligand results in negligible activation. Bars indicate mean activity; individual data points represent independent triplicates, performed on the same day. Fold change compared to cells expressing A- B- and Γ- type$_{JAK/STAT}$ receptors incubated with no ligand in noted above the bars. Significance (ANOVA with Dunnett's multiple comparison test) is noted above bars (Supplementary Table S4). ns: p>0.05, *p ≤0.05, **p ≤0.01, ***p ≤0.001.

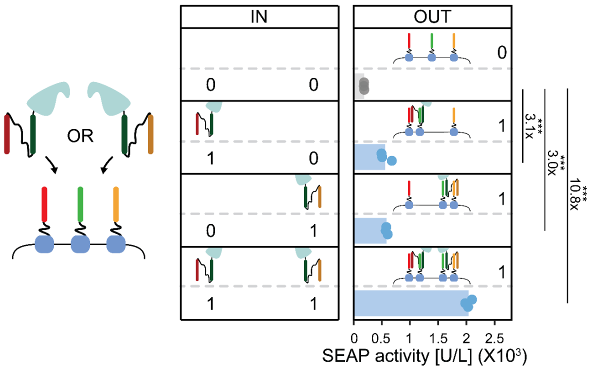

Supplementary Figure S18: OR gate operation using CC-GEMS. SEAP activity [U/L] in HEK293T cells transiently transfected with A-, B- and Γ-type$_{JAK/STAT}$ receptors with linker α_2 (8 aa, GS repeats), incubated with 0.5 µM SUMO-tagged purified ligand B'-A' and/or A'-Γ' with linker l_2 (see Methods). Left panel (IN; input) denotes the presence (1) or absence (0) of ditopic ligand. Right panel (OUT; output) shows SEAP activity [U/L]. Bars indicate mean activity; individual data points represent independent triplicates, performed on the same day. Fold change and significance (one way ANOVA with Dunnett's multiple comparison test) is noted besides the bars. ns: p>0.05, *p ≤0.05, **p ≤0.01, ***p ≤0.001. (see Supplementary Table S4).

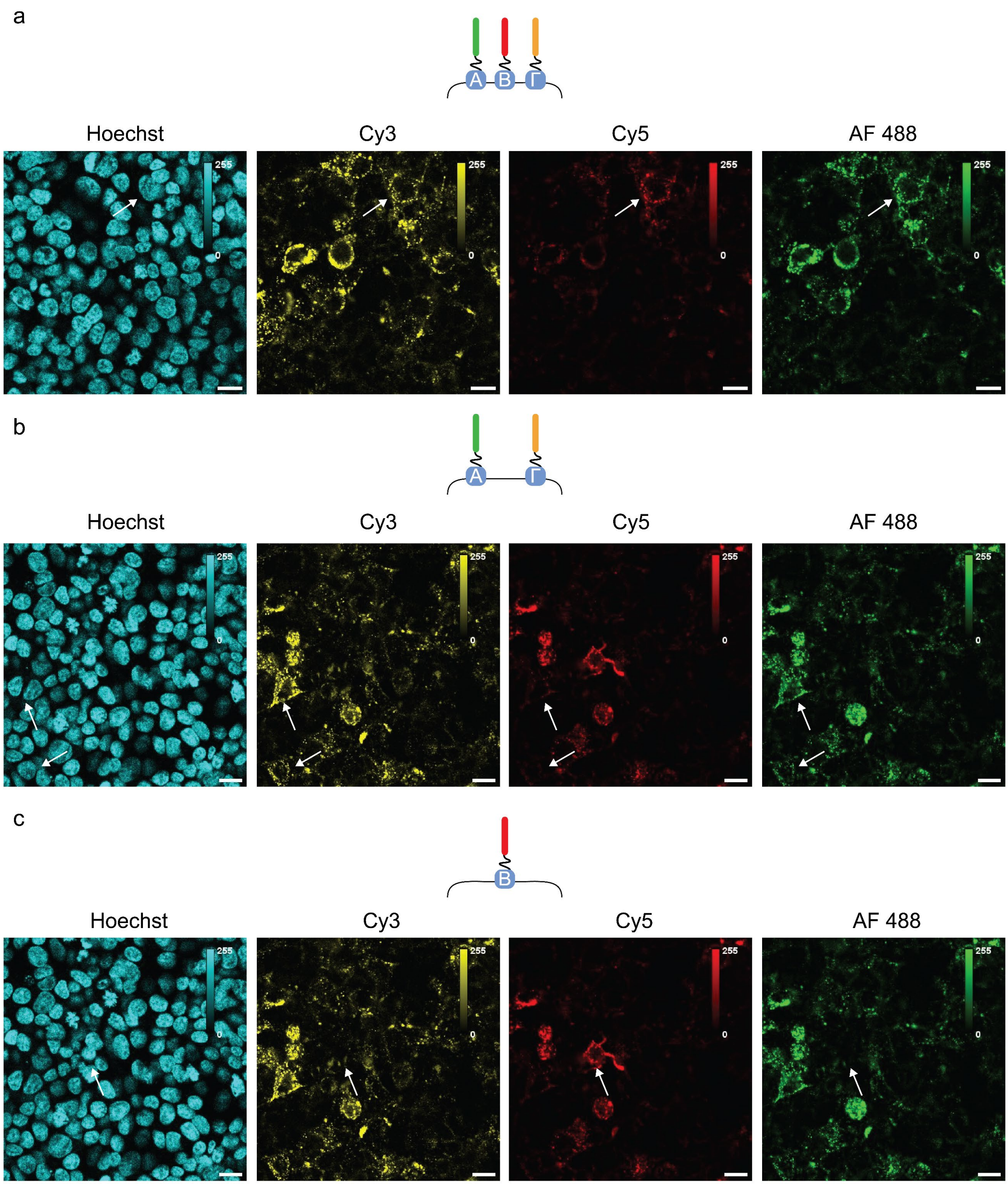

Supplementary Figure S19: Fluorescence confocal microscopy of cells transfected with A-type$_{JAK/STAT}$, B-type$_{JAK/STAT}$ and Γ-type$_{JAK/STAT}$ receptors incubated with Cy3-labelled A′-A′, Cy5-labelled B′-B′, and AF 488-labelled Γ′-Γ′ ligands. a Confocal micrograph images of HEK293T cells (indicated with arrowhead) expressing A-type$_{JAK/STAT}$, B-type$_{JAK/STAT}$, and Γ-type$_{JAK/STAT}$ receptor following incubation with 0.12 µM SUMO-tagged Cy3-labelled A′-A′, Cy5-labelled B′-B′, and AF 488-labelled Γ′-Γ′ dipeptides. **b** Confocal micrograph images of HEK293T cells (indicated with arrowhead) expressing A-type$_{JAK/STAT}$ and Γ-type$_{JAK/STAT}$ receptors following incubation with 0.12 µM SUMO-tagged Cy3-labelled A′-A′, Cy5-labelled B′-B′, and AF 488-labelled Γ′-Γ′ dipeptides. **c** Confocal micrograph images of HEK293T cell (indicated with arrowhead) expressing B-type$_{JAK/STAT}$ receptor following incubation with 0.12 µM SUMO-tagged Cy3-labelled A′-A′, Cy5-labelled B′-B′, and AF 488-labelled Γ′-Γ′ dipeptides. Cells were stained with Hoechst stain (blue). Cy3 in yellow, Cy5 in red, and AF 488 in green. Scale bar

(20 µm) is shown on the bottom-right of the image and intensity bar on top-right. Cy3 excitation: 553 nm, emission: 570-620 nm. Cy5 excitation: 647 nm, emission: 655-705 nm. AF 488 excitation: 488 nm, emission: 500-550 nm. Hoechst excitation: 405 nm, emission: 410-450 nm.

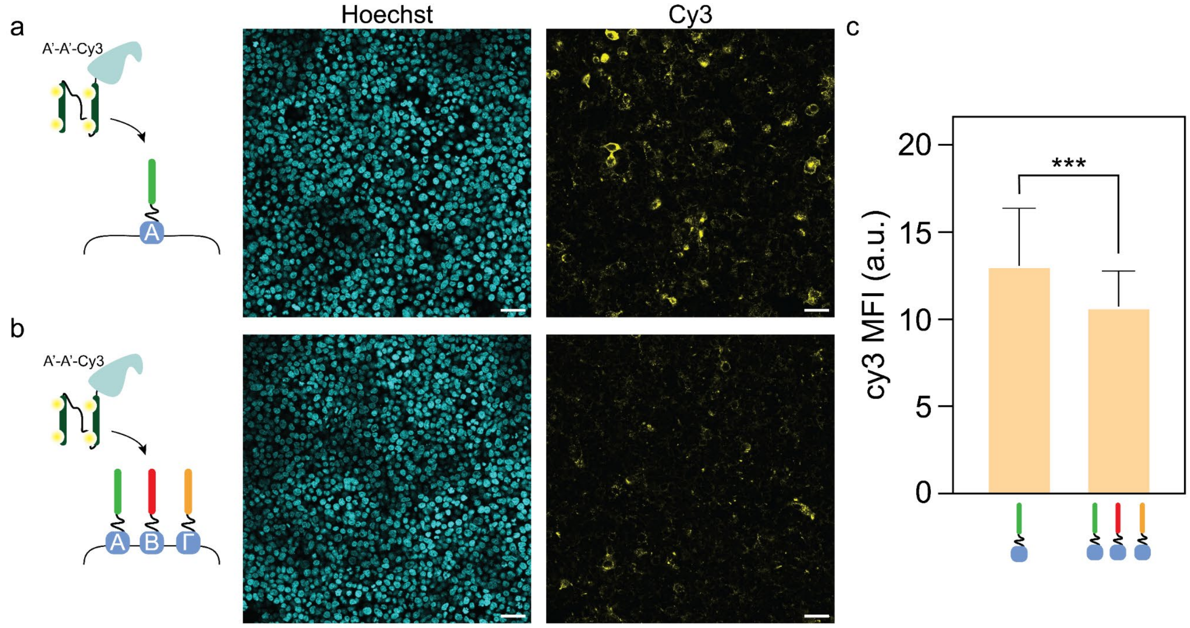

Supplementary Figure S20: Fluorescence confocal microscopy of cells transfected with one or three receptor types incubated with Cy3-labelled A'-A'. a Representative confocal micrograph images of HEK293T cells expressing A-type$_{JAK/STAT}$ receptor (Methods) following incubation with 0.12 µM Cy3-labelled, SUMO-tagged A'-A' dipeptide. **b** Representative confocal micrograph images of HEK293T cells expressing A-, B, and Γ-type$_{JAK/STAT}$ receptors (Methods) following incubation with Cy3-labelled, SUMO-tagged A'-A' dipeptide. **c** Cy3 Mean fluorescence intensity (MFI) in arbitrary units (a.u.) with standard deviation of n=12 regions of n=3 independent images (n_{total}=36) in cells expressing one (left bar) or three (right bar) receptors. Cells were stained with Hoechst stain (blue). Cy3 in yellow. Scale bar (50 µm) is shown on the bottom-right of the image. Cy3 excitation: 553 nm, emission: 570-620 nm. Hoechst excitation: 405 nm, emission: 410-450 nm. Significance (unpaired t-test) is noted above bars (see Supplementary Table S6). ns: p>0.05, *p ≤0.05, **p ≤0.01, ***p ≤0.001.

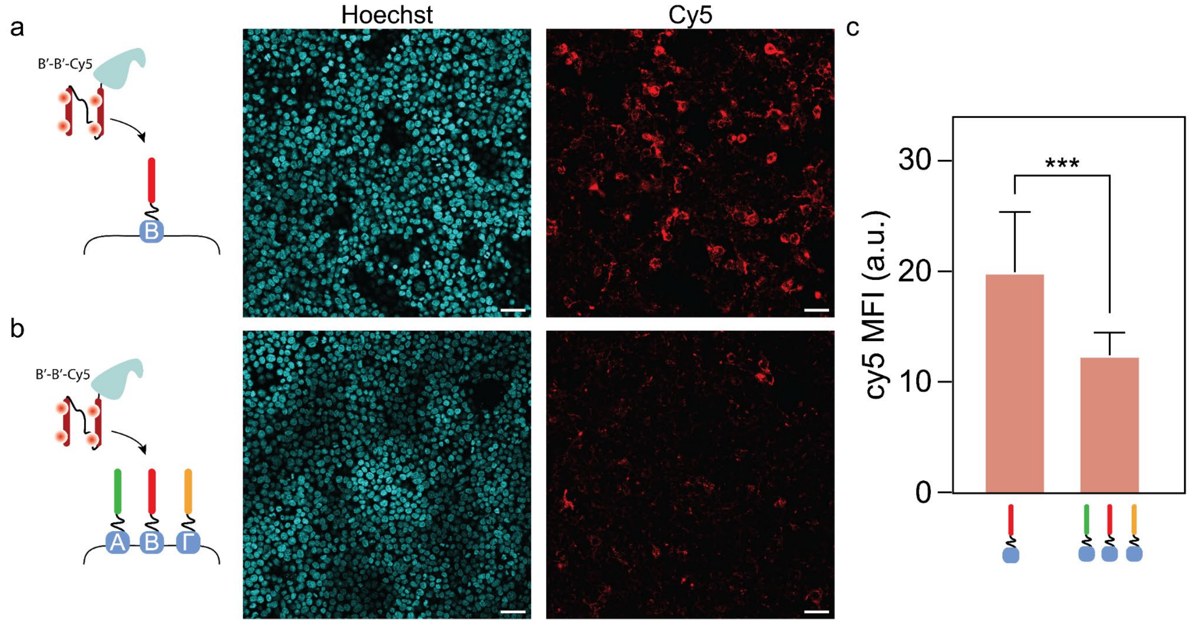

Supplementary Figure S21: Fluorescence confocal microscopy of cells transfected with one or three receptor types incubated with Cy5-labelled B′-B′. a Representative confocal micrograph images of HEK293T cells expressing B-type$_{JAK/STAT}$ receptor (Methods) following incubation with 0.12 µM Cy5-labelled, SUMO-tagged B′-B′ dipeptide. **b** Representative confocal micrograph images of HEK293T cells expressing A-, B, and Γ-type$_{JAK/STAT}$ receptors (Methods) following incubation with Cy5-labelled, SUMO-tagged B′-B′ dipeptide. **c** Cy5 Mean fluorescence intensity (MFI) in arbitrary units (a.u.) with standard deviation of n=12 regions of n=3 independent images (n_{total}=36) in cells expressing one (left bar) or three (right bar) receptors. Cells were stained with Hoechst stain (blue). Cy5 in red. Scale bar (50 µm) is shown on the bottom-right of the image. Cy5 excitation: 647 nm, emission: 655-705 nm. Hoechst excitation: 405 nm, emission: 410-450 nm. Significance (unpaired t-test) is noted above bars (see Supplementary Table S6). ns: p>0.05, *p ≤0.05, **p ≤0.01, ***p ≤0.001.

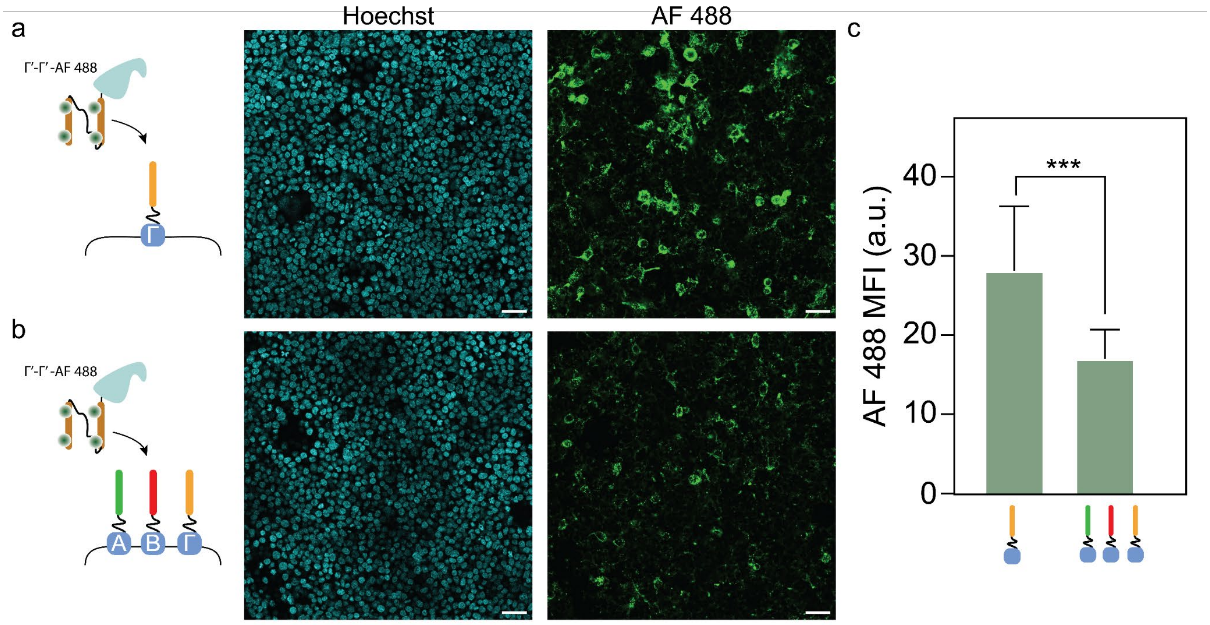

Supplementary Figure S22: Fluorescence confocal microscopy of cells transfected with one or three receptor types incubated with AF 488-labelled Γ′-Γ′. a Representative confocal micrograph images of HEK293T cells expressing Γ-type$_{JAK/STAT}$ receptor (Methods) following incubation with0.12 µM AF 488-labelled, SUMO-tagged Γ′-Γ′ dipeptide. **b** Representative confocal micrograph images of HEK293T cells expressing A-, B, and Γ-type$_{JAK/STAT}$ receptor (Methods) following incubation with AF 488-labelled, SUMO-tagged Γ′-Γ′ dipeptide. **c** AF 488 Mean fluorescence intensity (MFI) in arbitrary units (a.u.) with standard deviation of n=12 regions of n=3 independent images (n_{total}=36) in cells expressing one (left bar) or three (right bar) receptors. Cells were stained with Hoechst stain (blue). AF 488 in green. Scale bar (50 µm) is shown on the bottom-right of the image. AF 488 excitation: 488 nm, emission: 500-550 nm. Hoechst excitation: 405 nm, emission: 410-450 nm. Significance (unpaired t-test) is noted above bars (see Supplementary Table S6). ns: p>0.05, *p ≤0.05, **p ≤0.01, ***p ≤0.001.

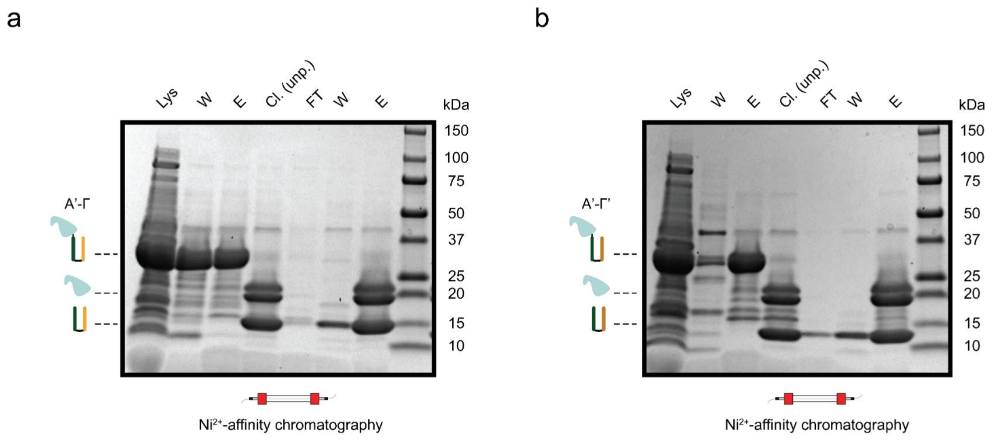

Supplementary Figure S23: A’-Γ and A’-Γ’ dipeptide ligand expression and purification. SDS-PAGE analysis, showing the expression of the engineered SUMO-tagged A’-Γ (**a**) and A’-Γ’ (**b**) CC dipeptides based on linker l_4, following purification with Ni^{2+}-affinity chromatography (Methods). Lys: cell lysate, FT: flow through, W: wash fraction, E: elution fraction. SUMO-tagged CC dipeptides were incubated with SUMO-hydrolase (Cl. (unp.)) to cleave of the SUMO-tag (see Methods).

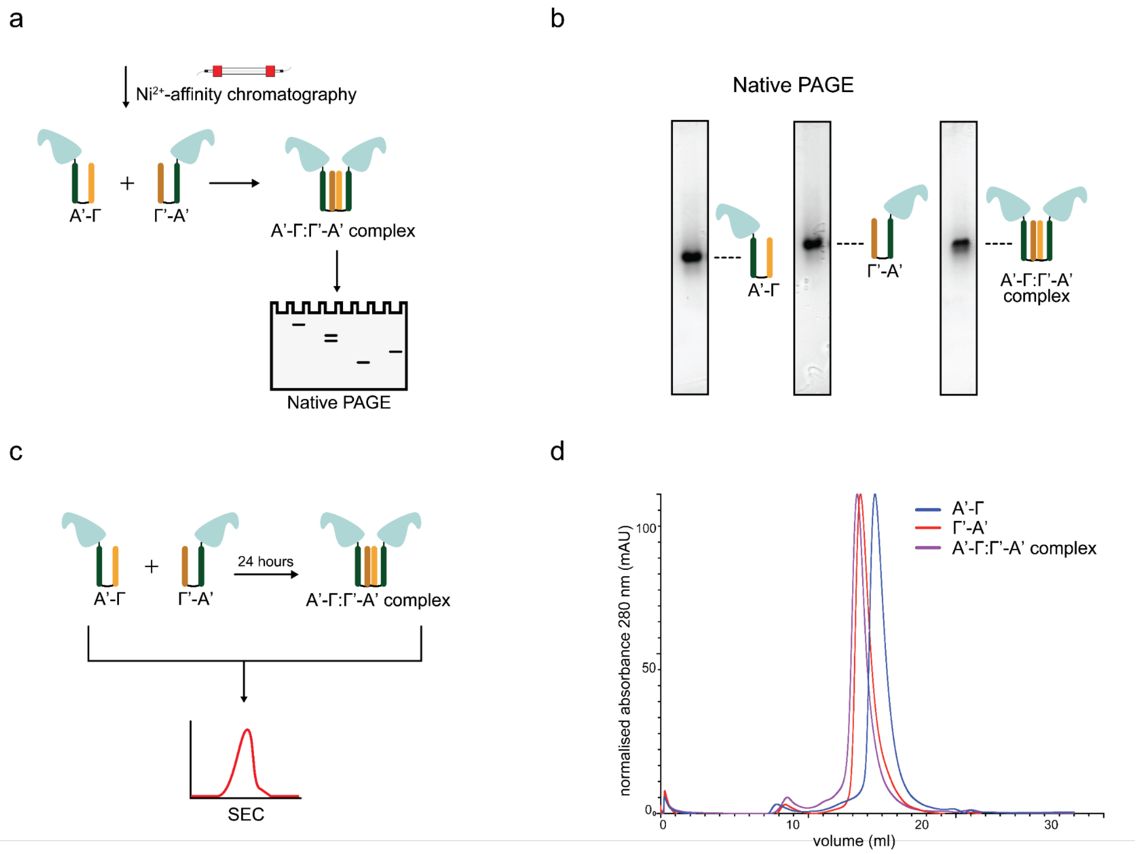

Supplementary Figure S24: Native PAGE of A’-Γ:Γ’-A’ complex. a SUMO-tagged A’-Γ and A’-Γ’ dipeptides with protein linker l_4 (see Supplementary Table S7) are purified using Ni^{2+}-affinity chromatography and subsequently incubated for 1 hour at 30°C (see Methods). The product is run on a Native-PAGE gel to assess A’-Γ:Γ’-A’ complex formation (see Methods). **b** Native-PAGE analysis of purified A’-Γ, A’-Γ’ and A’-Γ:Γ’-A’ complex, showing a distinct band for all three products. Proteins were run in the same gel. **c** Equimolar concentrations of SUMO-tagged A’-Γ and A’-Γ’ dipeptides with protein linker l_4 were incubated for 24 hours at 30°C and were subsequently characterised using Size Exclusion Chromatography (SEC; see Methods). **d** SEC analysis for SUMO-tagged A’-Γ

(blue line) and A’-Γ’ (red line) dipeptides as well as A’-Γ:Γ’-A’ complex after incubation of 24 hours (purple line). Incubation of dipeptides for 24 hours resulted in a distinct chromatogram peak indicating complex formation.

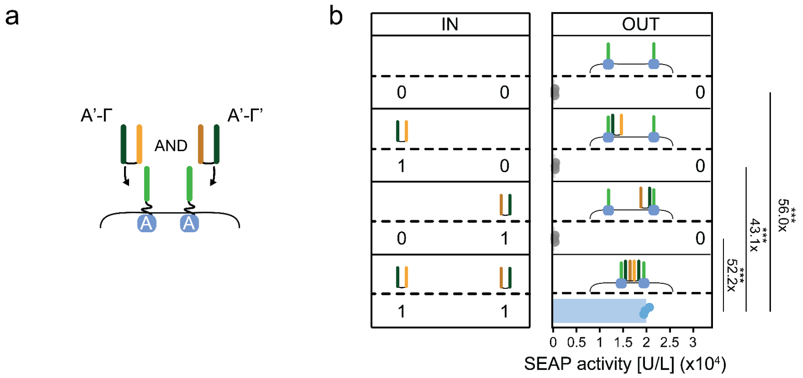

Supplementary Figure S25: A’-Γ and A’-Γ’ based AND gate logic in cells expressing A-type$_{JAK/STAT}$ receptor. a HEK293T cells expressing A-type$_{JAK/STAT}$ receptor and reporter were incubated with either both A’-Γ and A’-Γ’ ligands lacking the SUMO tag or each individual A’-Γ or A’-Γ’ ligands. **b** SEAP activity [U/L] in HEK293T cells transiently transfected with A-type$_{JAK/STAT}$ receptor with linker α_2 (8 aa, GS repeats), STAT3, and SEAP reporter and incubated with 0.12 µM purified ligand A’- Γ and/or Γ’-A (without the SUMO-tag). SEAP activity was measured after 48 hours incubation with ligand (see Methods). Left panel (IN; input) denotes the presence (1) or absence (0) of ditopic ligand. Right panel (OUT; output) shows SEAP activity [U/L]. Bars indicate mean activity; individual data points represent independent triplicates, performed on the same day. Fold change and significance (ANOVA with Tukey’s multiple comparison test; Supplementary Table S4) is noted above bars. ns: p>0.05, *p ≤0.05, **p ≤0.01, ***p ≤0.001.

a

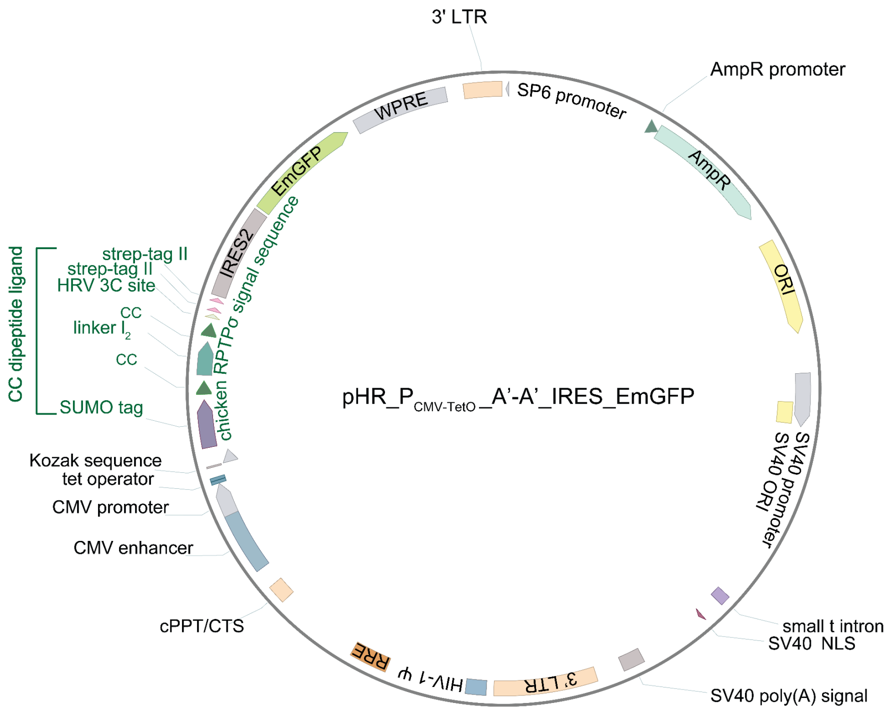

b

```
atg ggt atc ctt ccc agc cct ggg atg cct gcg ctg ctc tcc ctc gtg agc ctt ctc tcc gtg ctg ctg atg ggt tgc gta gct gaa acc ggt
 M   G   I   L   P   S   P   G   M   P   A   L   L   S   L   V   S   L   L   S   V   L   L   M   G   C   V   A   E   T   G

agc agc ggc act agt agc ggc ggt ggt agc gat agc gaa gtc aat caa gaa gcg aaa ccg gaa gtc gaa ccg gaa gtc aaa ccg gaa acg cac
 S   S   G   T   S   S   G   G   G   S   D   S   E   V   N   Q   E   A   K   P   E   V   E   P   E   V   K   P   E   T   H

atc aac ctg aaa gtt agt gat ggc agc tct gaa att ttc ttt aaa att aag aaa acc acg ccg ctg cgt cgc ctg atg gaa gcg ttt gcc aaa
 I   N   L   K   V   S   D   G   S   S   E   I   F   F   K   I   K   K   T   T   P   L   R   R   L   M   E   A   F   A   K

cgc cag ggc aaa gaa atg gat agc ctg cgc ttc ctg tat gac ggt att cgt atc cag gcg gat caa gcc ccg gaa gat ctg gac atg gaa gat
 R   Q   G   K   E   M   D   S   L   R   F   L   Y   D   G   I   R   I   Q   A   D   Q   A   P   E   D   L   D   M   E   D

aac gac att atc gaa gcg cat cgc gaa cag att ggc ggc cat atg agc ggt cct gga tcc tct ccg gaa gac aaa att gcg cag ttg aaa caa
 N   D   I   I   E   A   H   R   E   Q   I   G   G   H   M   S   G   P   G   S   S   P   E   D   K   I   A   Q   L   K   Q

aaa att cag gcg ctc aag caa gaa aat caa cag ttg gag gag gag aat gca gcc ctg gag tat ggt gga agt cga att cgg tgg gaa ttt cat
 K   I   Q   A   L   K   Q   E   N   Q   Q   L   E   E   E   N   A   A   L   E   Y   G   G   S   R   I   R   W   E   F   H

cac ggg ggt tcg ggt ggt tca ggc ggc tca gga ggc tcc ggg ggt tcc gga ggg agc ggt gct gaa gcc gca gcc aag gaa gca gca gct aaa
 H   G   G   S   G   G   S   G   G   S   G   G   S   G   G   S   G   G   S   G   A   E   A   A   A   K   E   A   A   A   K

gag gcc gct gcg aag gaa gct gcc gca aag gag gcg gcg gcg aaa gag gcg gca gca aaa gcc gga tct ggt ggc agt ggt ggc tcc ggc ggg
 E   A   A   A   K   E   A   A   A   K   E   A   A   A   K   E   A   A   A   K   A   G   S   G   G   S   G   G   S   G   G

tca ggt ggc agc ggg gga tca gga ggt acc agc ggt cct gga tcc tct ccg gaa gac aaa att gcg cag ttg aaa caa aaa att cag gcg ctc
 S   G   G   S   G   G   S   G   G   T   S   G   P   G   S   S   P   E   D   K   I   A   Q   L   K   Q   K   I   Q   A   L

aag caa gaa aat caa cag ttg gag gag gag aat gca gcc ctg gag tat ggt gga agt aag ctt tgg agc cac acg cgt ctt gag gtg ctg ttt
 K   Q   E   N   Q   Q   L   E   E   E   N   A   A   L   E   Y   G   G   S   K   L   W   S   H   T   R   L   E   V   L   F

cag gga cca gga ggt agt gga tct gct tgg agc cat cca cag ttc gaa aaa ggt gga ggt tct ggc ggt gga tca ggt gga agt gca tgg tct
 Q   G   P   G   G   S   G   S   A   W   S   H   P   Q   F   E   K   G   G   G   S   G   G   G   S   G   G   S   A   W   S

cac cct cag ttt gag aaa taa
 H   P   Q   F   E   K   *
```

chicken RPTPσ signal sequence | SUMO-tag | A' CC | linker l2 | strep-tag II

Supplementary Figure S26: Plasmid map and sequence of pHR expression vector for the engineering of sender cells expressing A'-A' bifunctional ligand. a Plasmid map of lentiviral vector containing the A'-A' bifunctional ligand (denoted here in green, see Supplementary Table S3). The ligand consists of a chicken RPTPσ signal sequence for secretion, a SUMO-tag, two CCs spanned by a linker l_2 (see Supplementary Table S7) and a Strep-tag II for subsequent purification (see Methods). The construct is under the control of a mammalian human cytomegalovirus (CMV) enhancer and promoter and two *TetO* operator sequences[4]. The construct is followed by an internal ribosome entry site (IRES) from encephalomyocarditis virus (EMCV) followed by an emerald green fluorescent protein (EmGFP); allowing bicistronic expression of the A'-A' dipeptide and EmGFP. WPRE: Woodchuck Hepatitis Virus posttranscriptional regulatory element, LTR: long terminal repeat, AmpR: ampicillin

resistance, ORI: origin of replication, SV40: Simian vacuolating virus 40, HIV-1 ψ: retroviral psi packaging element, RRE: Rev response element, cPPT/CTS: central polypurine tract/central termination sequence. **b** DNA and protein sequence of the A'-A' bifunctional ligand. The single-letter amino acid code is shown in uppercase below the corresponding DNA sequence. Chicken RPTPσ signal sequence is shown in red; SUMO-tag in purple; A' CC in dark green; linker l_2 in light green; Strep-tag II in pink.

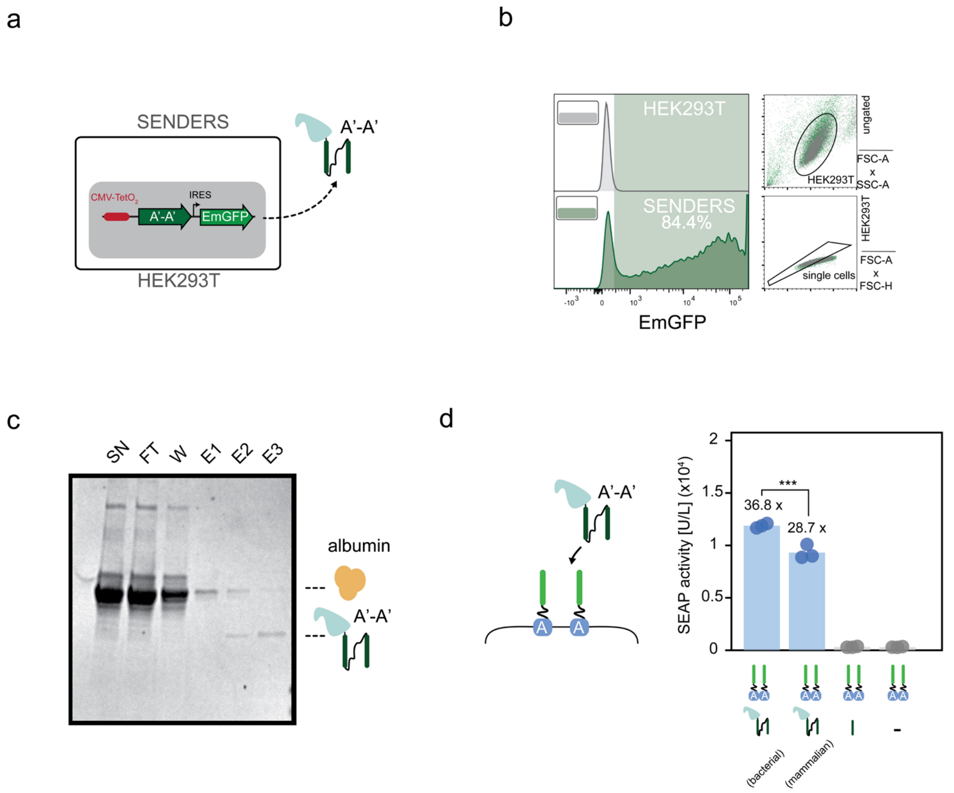

Supplementary Figure S27: Engineering of sender HEK293T cells expressing a A'-A' dipeptide. a HEK293T cells were stably transduced to express and secrete a SUMO-tagged A'-A' bifunctional ligand in the medium (with linker l_2; fused to chicken RPTPσ secretion signal sequence) under the control of CMV-TetO$_2$ promoter. An EmGFP fluorescent protein is also expressed as a bicistron (see Methods and Supplementary Figure S26). **b** Flow cytometry analysis of sender HEK293T cells (green) and control (untransduced HEK293T; grey), showing the gating strategy (dot plots; FSC-A x SSC-A and FSC-H x FSC-H). In the sender population, 84.4% of the cells were EmGFP positive. Backgating is shown on the right. **c** Detection of a A'-A' ligand expressed in mammalian cells, using SDS-PAGE analysis. The presence of albumin is also denoted. **d** SEAP activity [U/L] in HEK293T cells transiently transfected with A-type$_{JAK/STAT}$ receptor with linker α_2 (8 aa, GS repeats), and incubated with 0.12 µM SUMO-tagged purified A'-A' ligand expressed in bacterial or mammalian cells or a monomeric A' CC for 48 hours (see Methods). Bars indicate mean activity; individual data points represent independent triplicates. IRES: internal ribosome entry site, SN: supernatant, FT: flow through, W: wash fraction, E1: elution fraction 1, E2: elution fraction 2, E3: elution fraction 3. Bars indicate mean activity; individual data points represent independent triplicates, performed on the same day. Significance (ANOVA with Šidák's multiple comparison test, see Supplementary Table S4) is noted above bars. ns: $p>0.05$, *$p \leq 0.05$, **$p \leq 0.01$, ***$p \leq 0.001$. Fold change compared to cells incubated with A' monomer is noted above the bars.

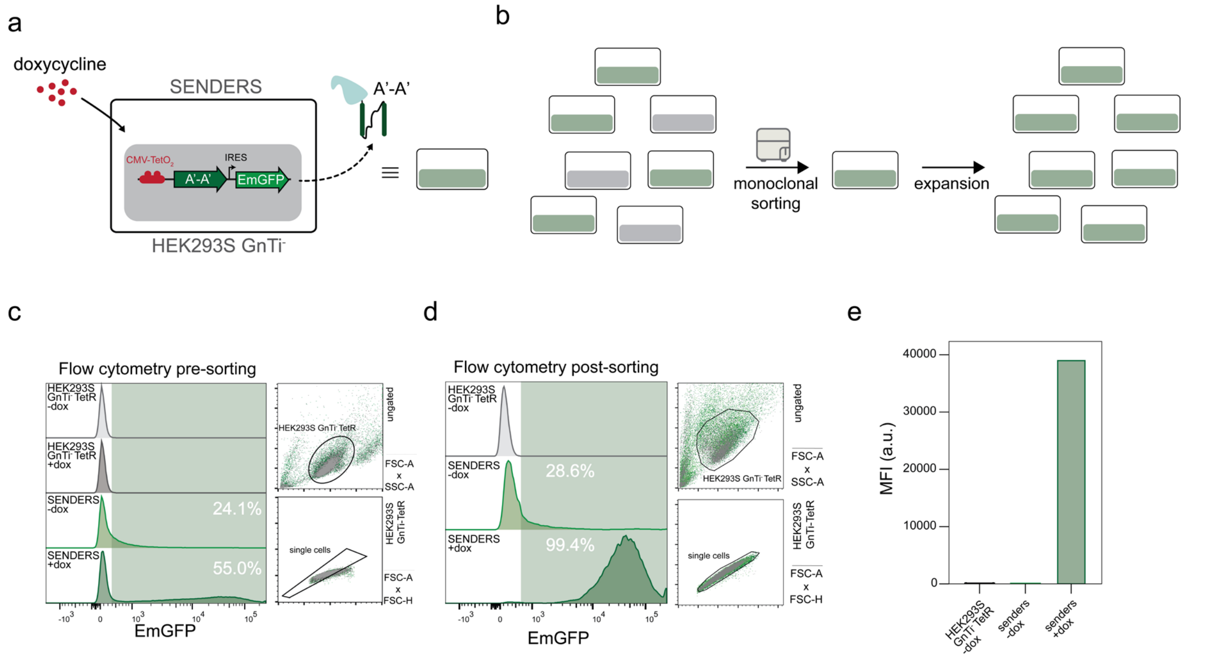

Supplementary Figure S28: Engineering of sender HEK293S GnTi$^-$ TetR cells expressing a A'-A' dipeptide. a HEK293S Gnti$^-$ TetR cells were stably transduced to, upon induction with doxycycline (dox), secrete a SUMO-tagged A'-A' bifunctional ligand (with linker l_2) fused to chicken RPTPσ secretion signal sequence, under the control of CMV-TetO$_2$ promoter. An EmGFP fluorescent protein is also expressed as a bicistron (see Methods and Supplementary Figure S26). **b** To enrich the population of cells expressing the gene, a monoclonal population of cells were sorted based on EmGFP intensity (see Methods). **c** Flow cytometry analysis of sender HEK293S Gnti$^-$ TetR cells (green) and control (untransduced HEK293S GnTi$^-$ TetR cells; grey) prior to sorting, showing the gating strategy on the right (dot plots; FSC-A x SSC-A and FSC-A x FSC-H). In the sender population and without the addition of dox 24.1% of the cells were EmGFP positive. The addition of dox results in 55.0% EmGFP positive cells. **d** Flow cytometry analysis of sender HEK293S Gnti$^-$ TetR cells (green) and control (untransduced HEK293S GnTi$^-$ TetR cells; grey) following sorting (see Methods). In the sender population and without the addition of dox 28.6% of the cells were EmGFP positive. The addition of dox results in 99.4% EmGFP positive cells. Gating (dot plots; FSC-A x SSC-A and FSC-A x FSC-H) is shown on the right. **e** Median fluorescent intensity (MFI; a.u.) of EmGFP of control and sender cells with and without the addition of dox. IRES: internal ribosome entry site.

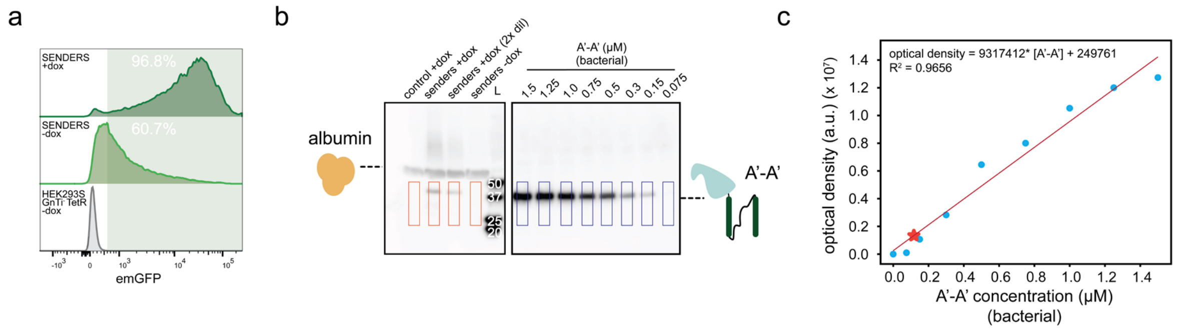

Supplementary Figure S29: A'-A' dipeptide quantification secreted by sender cells. a Flow cytometry analysis of sender HEK293S Gnti$^-$ TetR cells (green) and control (untransduced HEK293S GnTi$^-$ TetR cells; grey) with and without the addition of dox, used to quantify secreted SUMO-tagged A'-A' dipeptide ligand with l_2 (see Methods). **b** SUMO-tagged A'-A' dipeptide secreted by 4.5×10^5 plated sender cells was detected in the medium, following incubation with doxycycline for 48 hours, by means of western blotting (using an anti-Smt3 antibody; Methods). **c** Quantification of a A'-A' ligand expressed in mammalian cells, using gel densitometry analysis

(Methods). Analysis showing the expressed A’-A’ dipeptide (orange rectangles) as well as a titration of 8 known concentrations of SUMO-tag A’-A’ dipeptide expressed in bacteria (blue rectangles). A calibration curve was obtained by estimating the optical density (a.u.) of the known concentrations of the SUMO-tagged A’-A’ dipeptide ligand expressed in bacteria and fitting the data using linear regression (see Methods). optical density = 9317412 * [A’-A’] + 249761, R^2=0.9656. Calculated concentration of SUMO-tagged A’-A’ dipeptide secreted from the senders is denoted on the graph as a red star (0.14 µM).

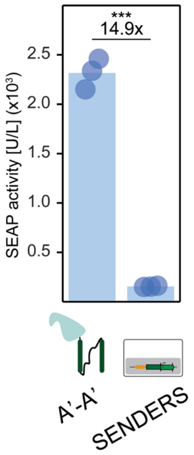

Supplementary Figure S30: Comparison between ligand-induced receptor activation and receptor activation via sender cells. SEAP activity [U/L] in HEK293T cells transiently transfected with A-type$_{JAK/STAT}$ receptor (α_2=8 aa) subsequently incubated with 0.12 µM ditopic SUMO-tagged A’-A’ ligand or 4.5 x 10^5 sender cells for 48h, in the presence of doxycycline (see Methods). Bars indicate mean activity; individual data points represent independent triplicates, performed on the same day. Fold change and significance (unpaired t-test) is noted above bars (see Supplementary Table S6). ns: p>0.05, *p ≤0.05, **p ≤0.01, ***p ≤0.001.

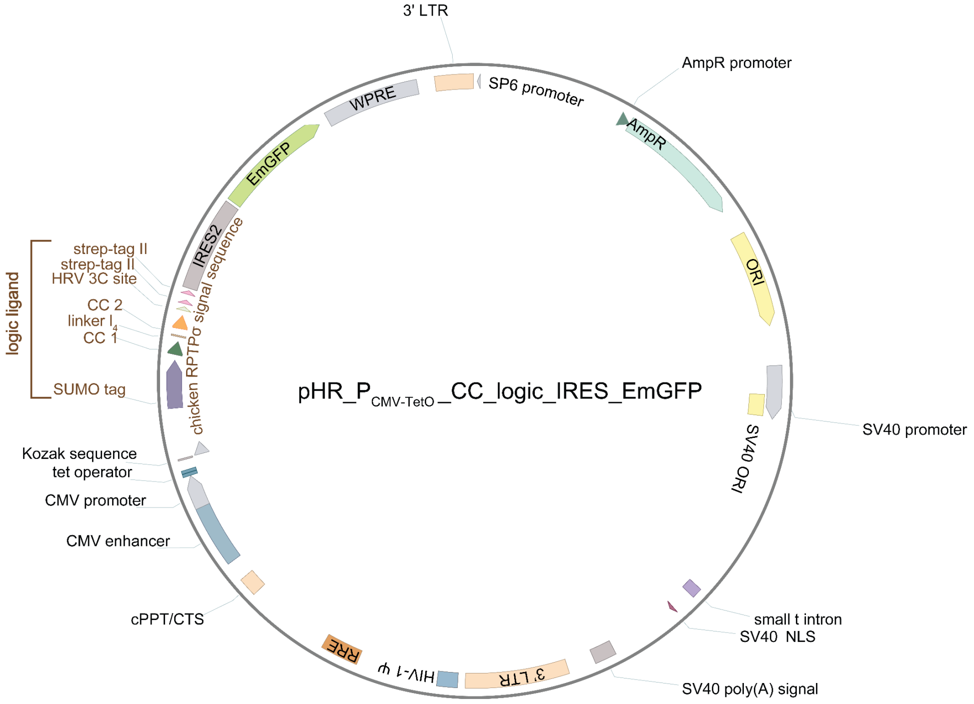

Supplementary Figure S31: Plasmid map and sequence of pHR expression vector expressing A'-Γ and Γ'-A' ditopic ligands for AND gate logic. a Plasmid map of lentiviral vector containing the A'-Γ or Γ'-A' bifunctional ligand (denoted here as logic ligand; in brown). The ligand consists of a chicken RPTPσ signal sequence for secretion, a SUMO-tag, CC 1 (A' CC, see Supplementary Table S1), a linker l_4 (Supplementary Table S7), CC 2 (Γ or Γ' CC Supplementary Table S1) and a twin strep-tag for subsequent purification (see Methods). The construct is under the control of a mammalian human cytomegalovirus (CMV) enhancer and promoter and two *TetO* operator sequences[4]. The construct is followed by an internal ribosome entry site (IRES) from encephalomyocarditis virus (EMCV) followed by an emerald green fluorescent protein (EmGFP); allowing bicistronic expression of the dipeptide and EmGFP. WPRE: Woodchuck Hepatitis Virus posttranscriptional regulatory element, LTR: long terminal repeat, AmpR: ampicillin resistance, ORI: origin of replication, SV40: Simian vacuolating virus 40, HIV-1 ψ: retroviral psi packaging element, RRE: Rev response element, cPPT/CTS: central polypurine tract/central termination sequence.

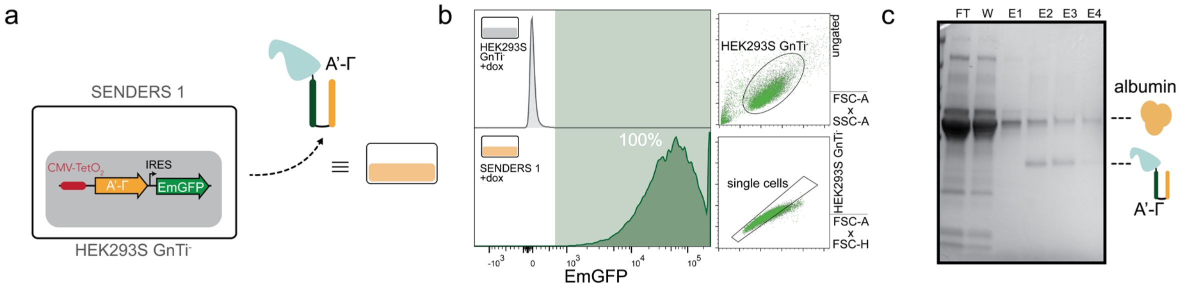

Supplementary Figure S32: Engineering of sender HEK293S GnTi⁻ cells expressing A'-Γ dipeptide. a HEK293S Gnti⁻ cells were stably transduced to excrete a SUMO-tagged A'-Γ bifunctional ligand (with linker l_4; fused to chicken RPTPσ secretion signal sequence) under the control of CMV-TetO_2 promoter (senders 1). An EmGFP fluorescent protein is also expressed as a bicistron (see Methods and Supplementary Figure S31). **b** Flow cytometry analysis of sender 1 cells (green) and control (untransduced HEK293S GnTi⁻ cells; grey) showing the gating strategy on the right (dot plots; FSC-A x SSC-A and FSC-A x FSC-H). **c** SDS-PAGE analysis, showing the expression of the engineered A'-Γ dipeptide expressed in sender 1 cells. The dipeptide was Strep-tag purified

from culturing medium, containing dox (see Methods). The presence of albumin is also denoted. IRES: internal ribosome entry site. FT: flow through, W: wash fraction, E1: elution fraction 1, E2: elution fraction 2, E3: elution fraction 3, E4: elution fraction 4.

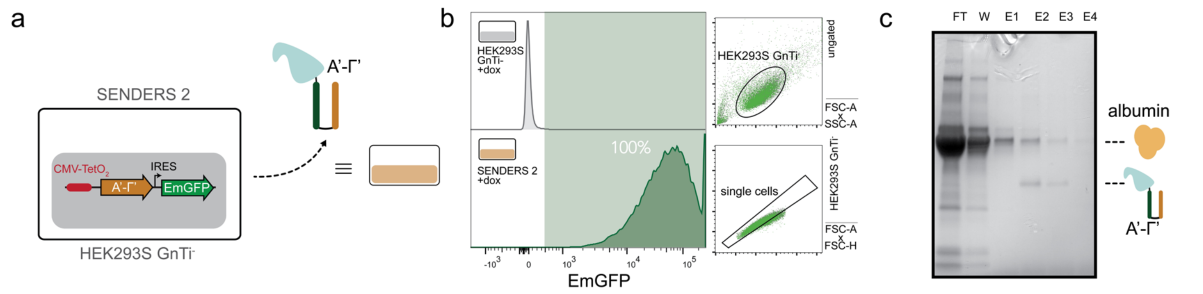

Supplementary Figure S33: Engineering of sender HEK293S GnTi⁻ cells expressing a Γ'-A' dipeptide. a HEK293S Gnti⁻ cells were stably transduced to excrete a SUMO-tagged A'-Γ' bifunctional ligand (with linker l_4; fused to chicken RPTPσ secretion signal sequence) under the control of CMV-TetO$_2$ promoter (senders 2). An EmGFP fluorescent protein is also expressed as a bicistron (see Methods and Supplementary Figure S31). **b** Flow cytometry analysis of sender 2 cells (green) and control (untransduced HEK293S GnTi⁻ cells; grey) showing the gating strategy on the right (dot plots; FSC-A x SSC-A and FSC-A x FSC-H). **c** SDS-PAGE analysis, showing the expression of the engineered A'-Γ' dipeptide expressed in sender 2 cells. The dipeptide was Strep-tag purified from culturing medium, containing dox (see Methods). The presence of albumin is also denoted. **d** Native-PAGE analysis of purified A'-Γ' ligand. IRES: internal ribosome entry site. FT: flow through, W: wash fraction, E1: elution fraction 1, E2: elution fraction 2, E3: elution fraction 3, E4: elution fraction 4.

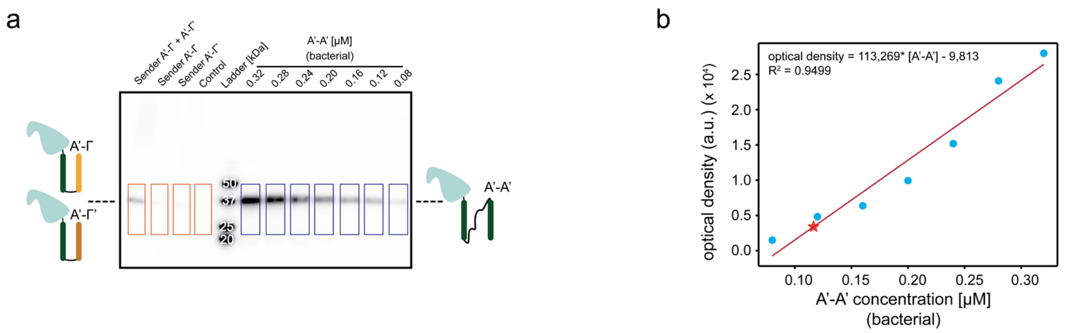

Supplementary Figure S34: A'-Γ-A'-Γ' complex dipeptide quantification secreted by sender 1 and 2 cells. a SUMO-tagged A'-Γ and SUMO-tagged A'-Γ' dipeptides secreted by 5 x 10^5 plated sender 1 and 5 x 10^5 Sender 2 cells (see Supplementary Figures S32 and S34) were detected in the medium, following incubation of cells for 48 hours, by means of western blotting (using an anti-Smt3 antibody; Methods). **b** Quantification of ligands expressed in mammalian cells, using gel densitometry analysis (Methods). Analysis showing the expressed A'-Γ-A'-Γ' complex dipeptide bearing two SUMO-tags (orange rectangles) as well as a titration of seven known concentrations of SUMO-tagged A'-A' dipeptide expressed in bacteria (blue rectangles; 0.08, 0.12, 0.16, 0.20, 0.24, 0.28, and 0.32 μM). A calibration curve was obtained by estimating the optical density (a.u.) of the known concentrations of the SUMO-tagged A'-A' dipeptide ligand expressed in bacteria and fitting the data using linear regression (see Methods). optical density = 113,269 * [A'-A'] + 9,813, R^2=0.9499. Calculated concentration of the SUMO-tag for the A'-Γ-A'-Γ' complex dipeptide secreted from the sender 1 and 2 cell populations is denoted on the graph as a red star (0.12 μM).

a

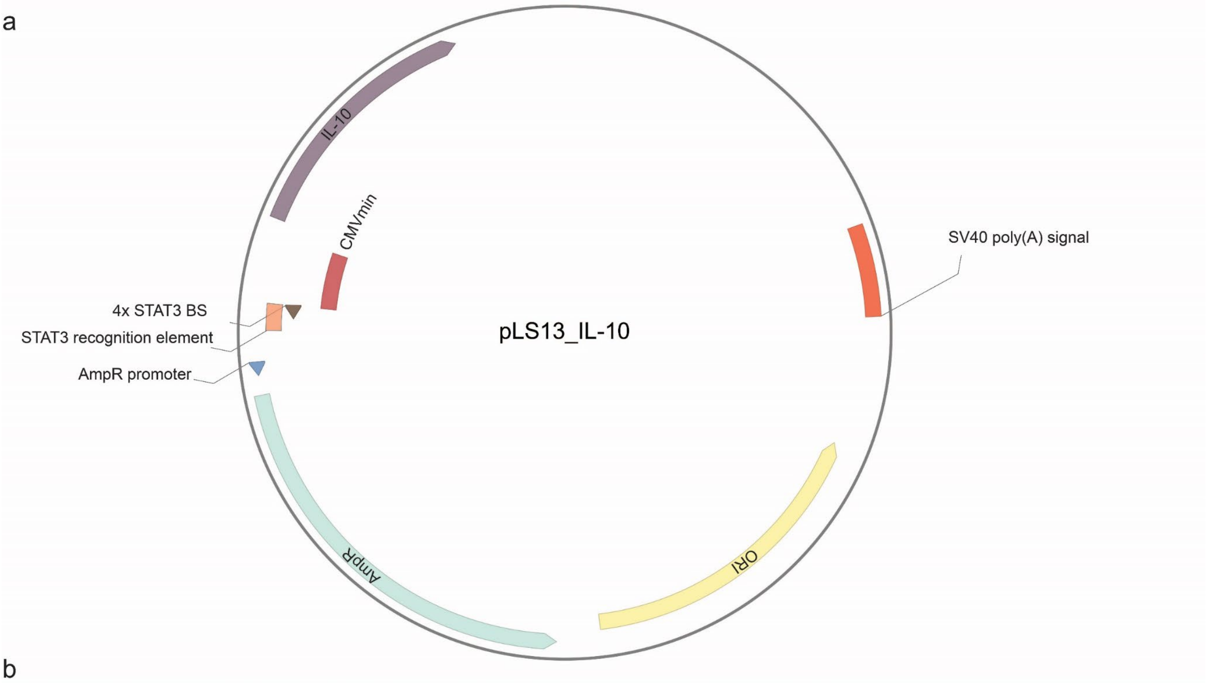

b

```
atg cac tca agc gct ctc ctt tgc tgt ctt gtt ctc ctc aca gga gta cgc gct tca ccc gga caa ggc aca cag
 M   H   S   S   A   L   L   C   C   L   V   L   L   T   G   V   R   A   S   P   G   Q   G   T   Q
tct gaa aat tca tgc aca cat ttt ccc ggc aac ctc cca aat atg ctt agg gat ctc cgc gac gcg ttt agt aga
 S   E   N   S   C   T   H   F   P   G   N   L   P   N   M   L   R   D   L   R   D   A   F   S   R
gta aag acg ttc ttt caa atg aag gac caa ttg gac aat ttg ctc ttg aag gag agc ctt ctc gaa gat ttc aag
 V   K   T   F   F   Q   M   K   D   Q   L   D   N   L   L   L   K   E   S   L   L   E   D   F   K
ggt tac ctt ggt tgt caa gcc ctt tct gag atg atc caa ttc tac ttg gag gaa gta atg cca caa gcg gaa aac
 G   Y   L   G   C   Q   A   L   S   E   M   I   Q   F   Y   L   E   E   V   M   P   Q   A   E   N
caa gat cct gat att aaa gct cat gta aat tct ctg gga gag aac ctc aag acc ttg agg ctc cgc ttg cga cgg
 Q   D   P   D   I   K   A   H   V   N   S   L   G   E   N   L   K   T   L   R   L   R   L   R   R
tgc cac aga ttc ttg cct tgc gaa aat aag agt aaa gcc gta gag caa gtc aaa aat gcg ttt aat aag ttg caa
 C   H   R   F   L   P   C   E   N   K   S   K   A   V   E   Q   V   K   N   A   F   N   K   L   Q
gag aag gga ata tat aag gcg atg tcc gag ttt gac ata ttc att aac tac att gag gct tat atg aca atg aag
 E   K   G   I   Y   K   A   M   S   E   F   D   I   F   I   N   Y   I   E   A   Y   M   T   M   K
atc cgc aac taa
 I   R   N   *
```

Supplementary Figure S35: Plasmid map and sequence for IL-10. a Plasmid map of mammalian expression vector for the expression of IL-10 under the control of a STAT3 recognition element. AmpR: ampicillin resistance, ORI: origin of replication, SV40: Simian vacuolating virus 40, CMVmin: minimal cytomegalovirus promoter. **b** DNA and protein sequence of the IL-10 ligand. The single-letter amino acid code is shown in uppercase below the corresponding DNA sequence.

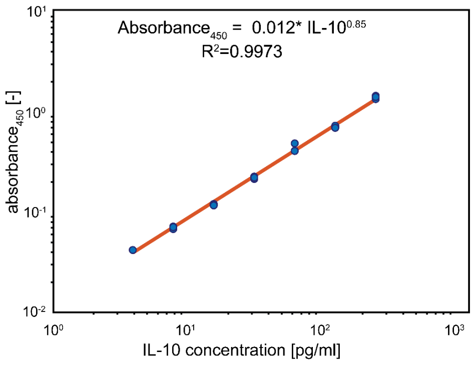

Supplementary Figure S36: IL-10 calibration curve. Calibration curve of known concentrations of IL-10. Absorbance units were measured at 450 nm (25°C). Data points were fitted using a linear regression curve.

Absorbance$_{450}$ = 0.012 * IL-10$^{0.85}$, R^2=0.9973. Individual data points represent technical replicates (n=3) (see Methods).

Supplementary Raw Data

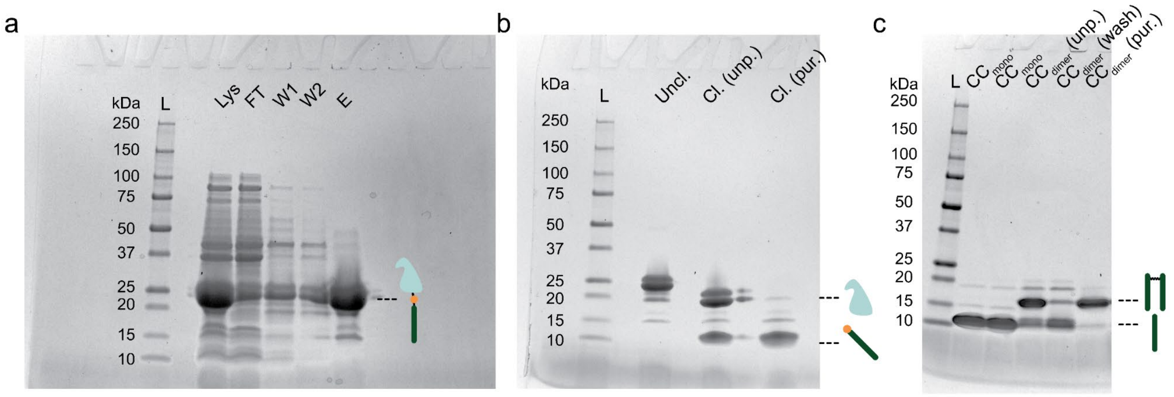

Supplementary Figure SR1: a Raw SDS-PAGE gel image of Supplementary Figure S4c. **b** Raw SDS-PAGE gel image of Supplementary Figure S4d. **c** Raw SDS-PAGE gel image of Figure 2c. L: molecular weight ladder, Lys: cell lysate, FT: flow through, W1: wash fraction 1, W2: wash fraction 2, E: elution fraction, Uncl.: uncleaved peptide, Cl. (unp.): cleaved peptide (unpurified), Cl. (pur.): cleaved peptide (purified), CC_{mono}: A' CC, CC_{dimer} (unp.): A'-A' dimer (unpurified), CC_{dimer} (wash): A'-A' dimer (wash fraction), CC_{dimer} (pur.): A'-A' dimer (purified).

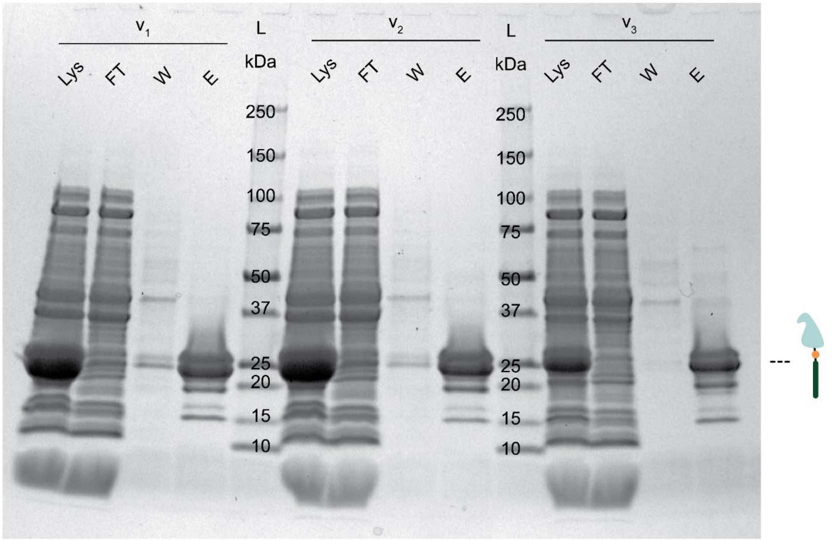

Supplementary Figure SR2: Raw SDS-PAGE gel image of Supplementary Figure S6b. L: molecular weight ladder, Lys: cell lysate, FT: flow through, W: wash fraction, E: elution fraction.

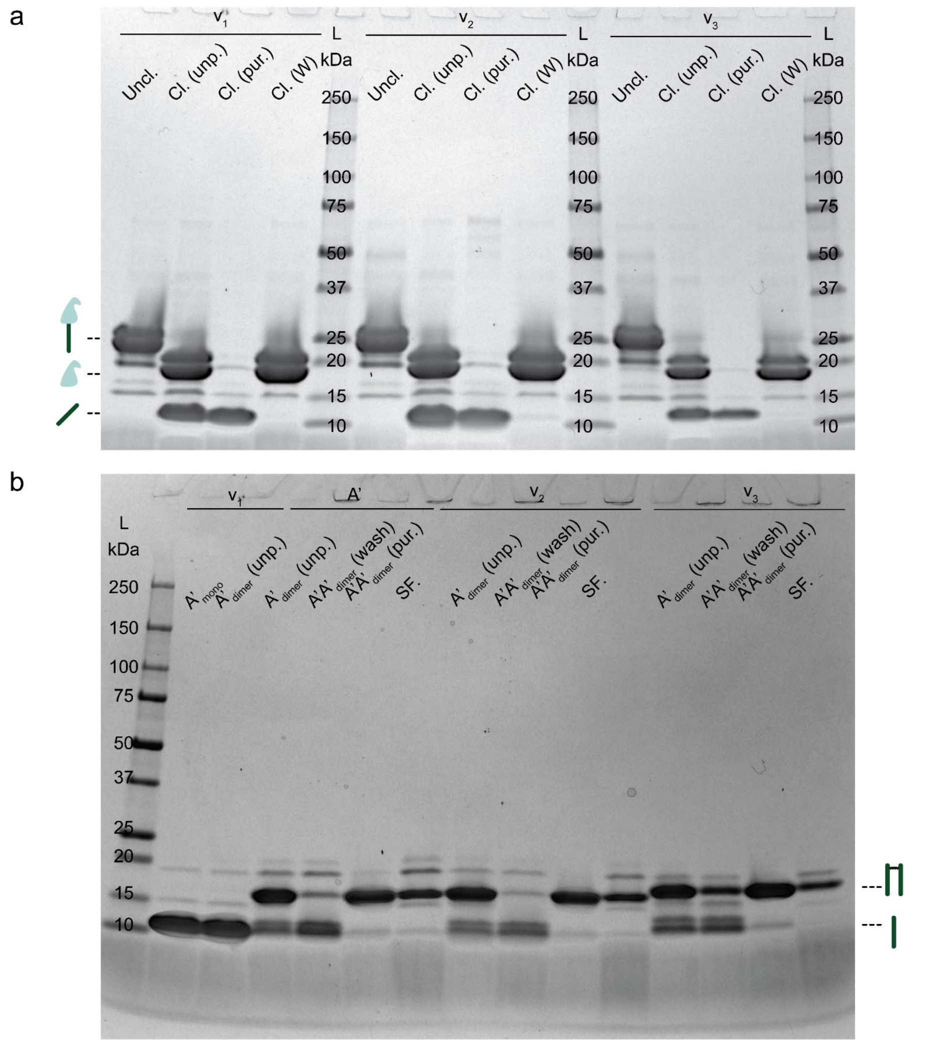

Supplementary Figure SR3: a and **b** Raw SDS-PAGE gel images of Supplementary Figure S6c, S6d, and S6e. Uncl.: uncleaved peptide, Cl. (unp.): cleaved peptide (unpurified), Cl. (pur.): cleaved peptide (purified), Cl. (W): cleaved peptide (wash fraction), A'_{mono}: A' CC monomer, $A'A'_{dimer}$(unp.): A'-A' CC (unpurified), $A'A'_{dimer}$(wash): A'-A' CC (wash fraction), $A'A'_{dimer}$(pur.): A'-A' CC (purified), SF: strip fraction.

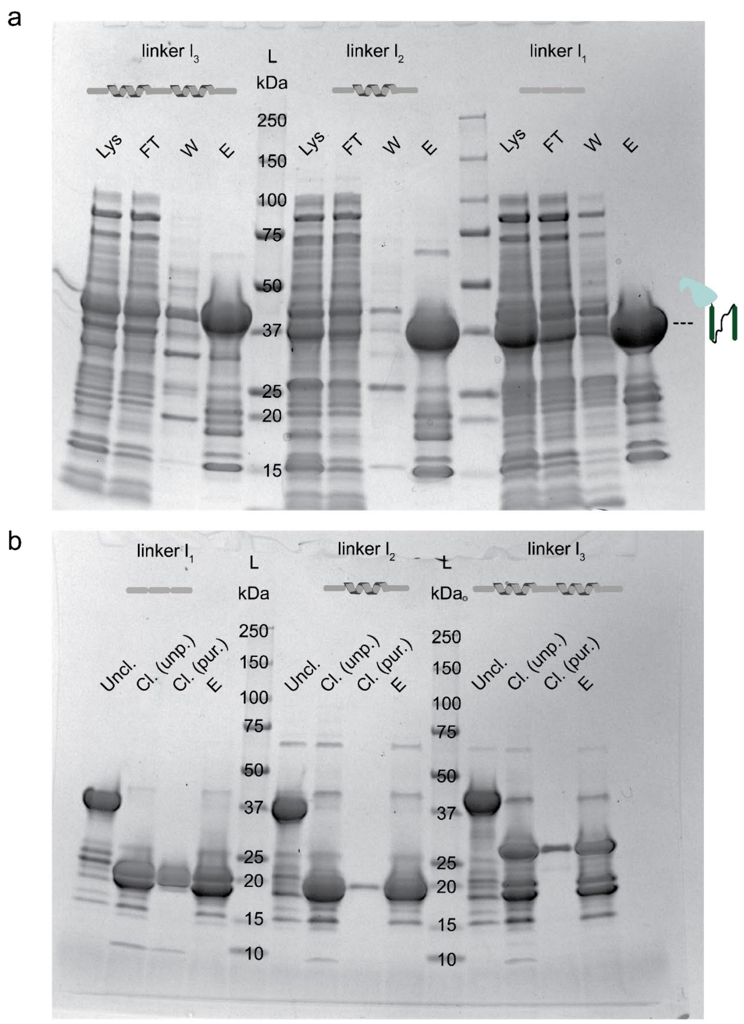

Supplementary Figure SR4: a and **b** Raw SDS-PAGE gel images of Supplementary Figure S8. L: molecular weight ladder, Lys: cell lysate, FT: flow through, W: wash fraction, E: elution fraction, Uncl.: uncleaved peptide, Cl. (unp.): cleaved peptide (unpurified), Cl. (pur.): cleaved peptide (purified).

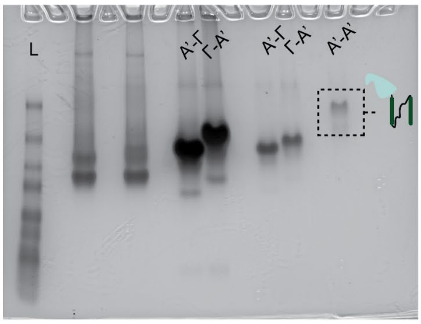

Supplementary Figure SR5: Raw Native-PAGE gel image of Supplementary Figure S9.

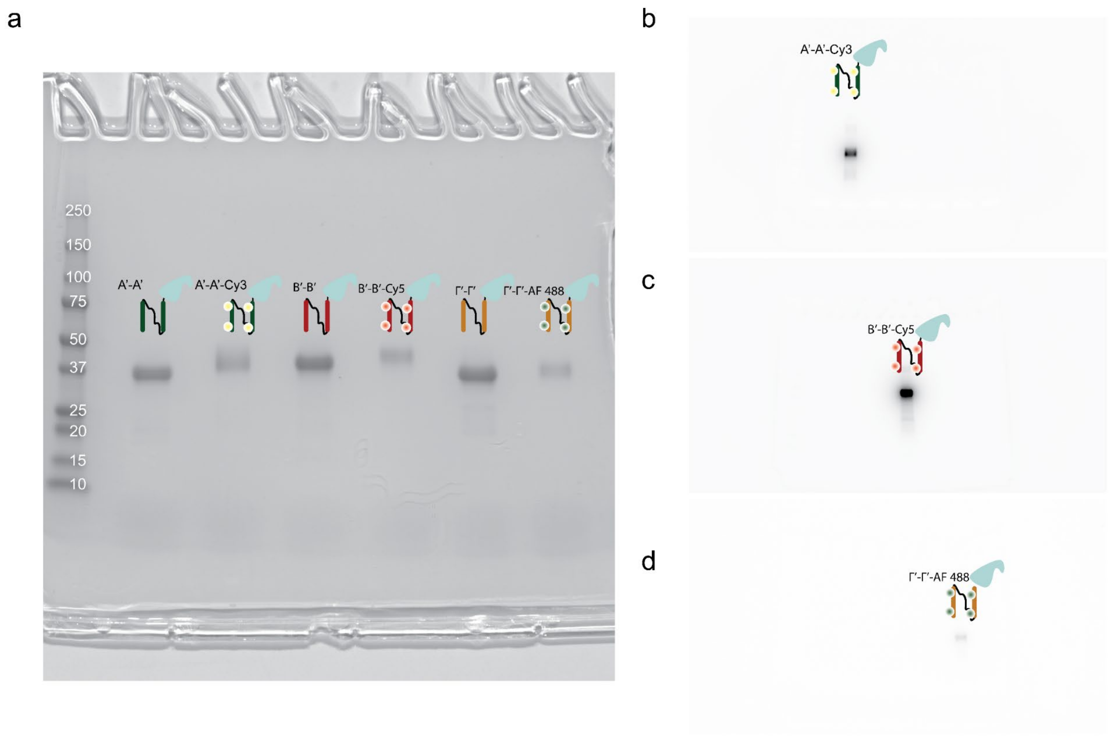

Supplementary Figure SR6: a Raw SDS-PAGE gel image of Supplementary Figure S10a. **b-d** Raw Fluorescence SDS-PAGE gel images of Supplementary Figure S10b. **b** corresponds to Cy3, **c** Cy5, and **d** AF-488.

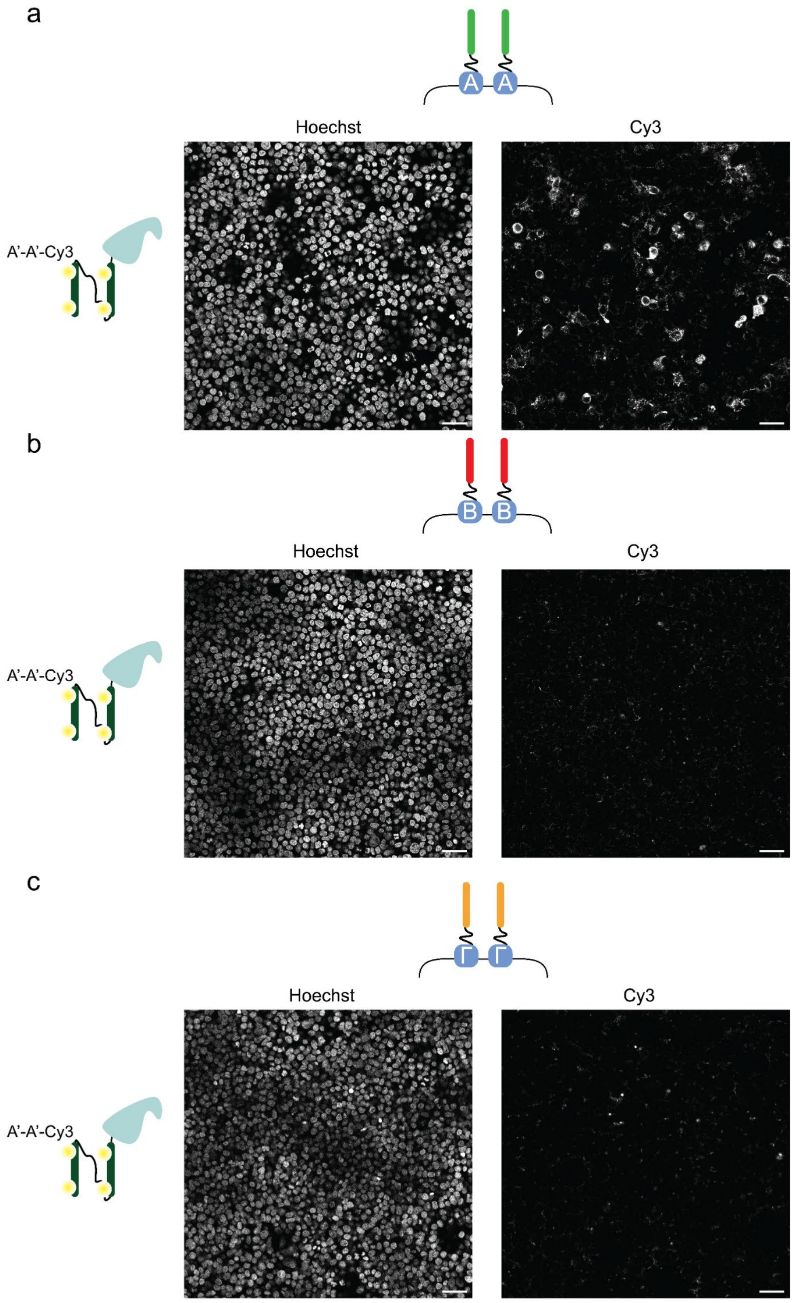

Supplementary Figure SR7: Grayscale, raw confocal micrograph images of **a** A-type$_{JAK/STAT}$ (corresponding to images Figure 3e), **b** B-type$_{JAK/STAT}$, and **c** Γ-type$_{JAK/STAT}$ cells (corresponding to Supplementary Figure S11) incubated with Cy3-labelled SUMO-tagged A'-A' dipeptide ligand. Scale bar (50 µm) is shown on the bottom-right of the image. Cy3 excitation: 553 nm, emission: 570-620 nm. Hoechst excitation: 405 nm, emission: 410-450 nm.

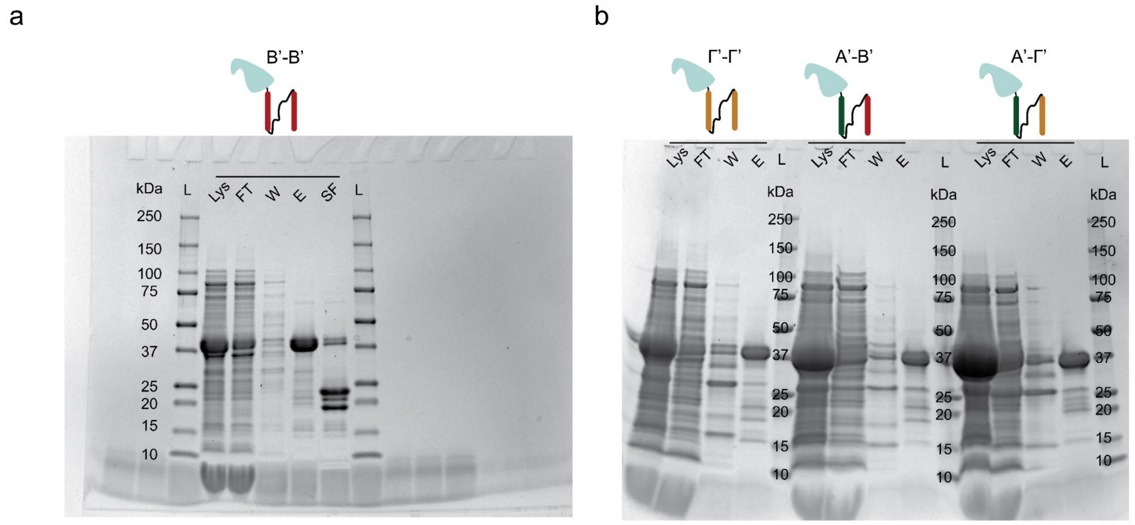

Supplementary Figure SR8: a and **b** Raw SDS-PAGE gel images of Supplementary Figure S13, S17a and S17b. L: molecular weight ladder, Lys: cell lysate, FT: flow through, W: wash fraction, E: elution fraction, SF: strip fraction.

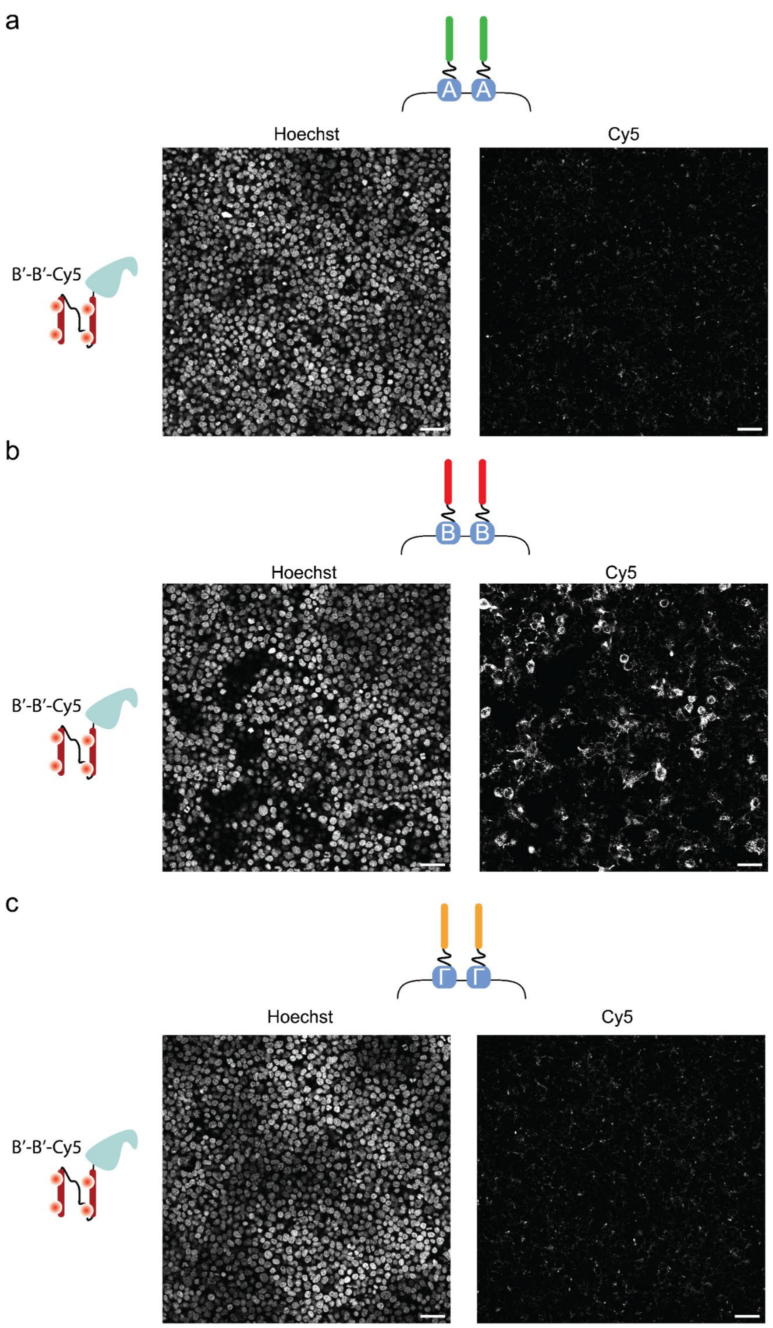

Supplementary Figure SR9: Grayscale, raw confocal micrograph images of **a** A-type$_{JAK/STAT}$, **b** B-type$_{JAK/STAT}$, and **c** Γ-type$_{JAK/STAT}$ cells (corresponding to Supplementary Figure S14) incubated with Cy5-labelled SUMO-tagged B′-B′ dipeptide ligand. Scale bar (50 µm) is shown on the bottom-right of the image. Cy5 excitation: 647 nm, emission: 655-705 nm. Hoechst excitation: 405 nm, emission: 410-450 nm.

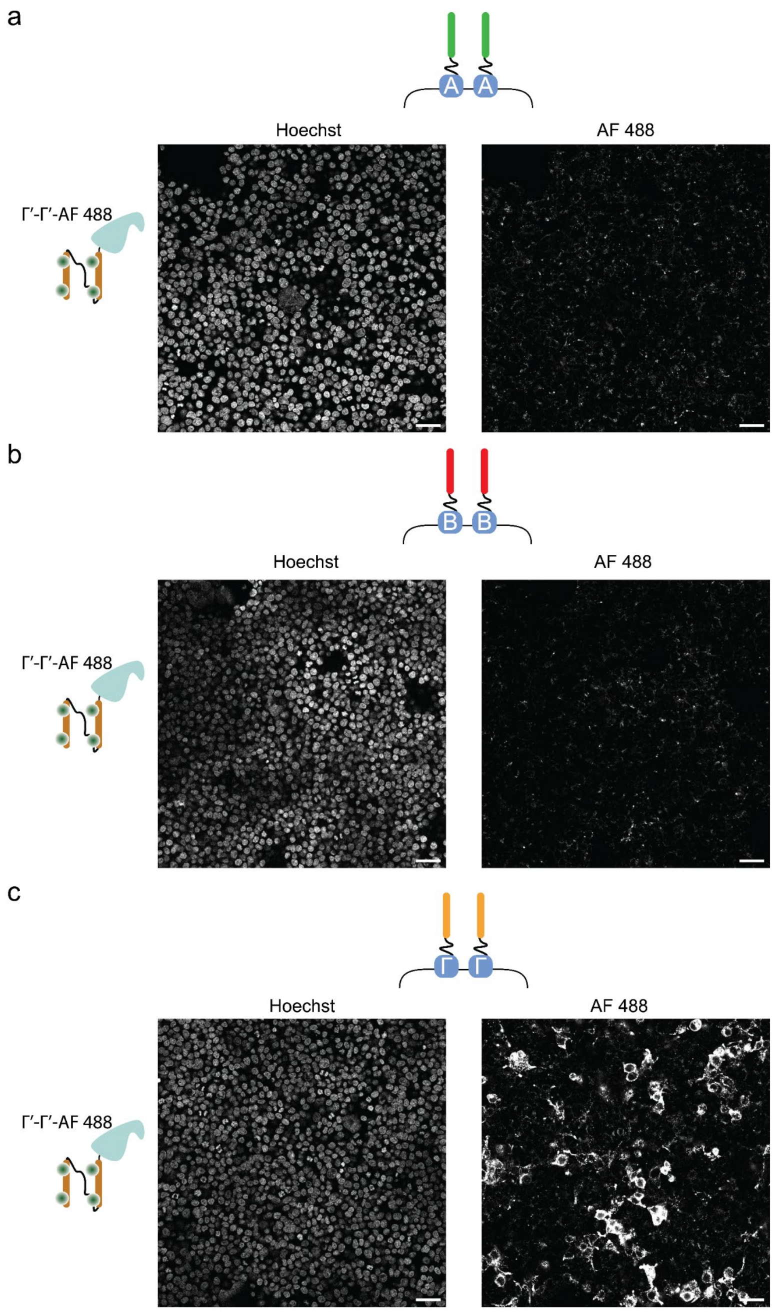

Supplementary Figure SR10: Grayscale, raw confocal micrograph images of **a** A-type$_{JAK/STAT}$, **b** B-type$_{JAK/STAT}$, and **c** Γ-type$_{JAK/STAT}$ cells (corresponding to Supplementary Figure S15) incubated with AF 488-labelled SUMO-tagged Γ′-Γ′ dipeptide ligand. Scale bar (50 µm) is shown on the bottom-right of the image. AF 488 excitation: 488 nm, emission: 500-550 nm. Hoechst excitation: 405 nm, emission: 410-450 nm.

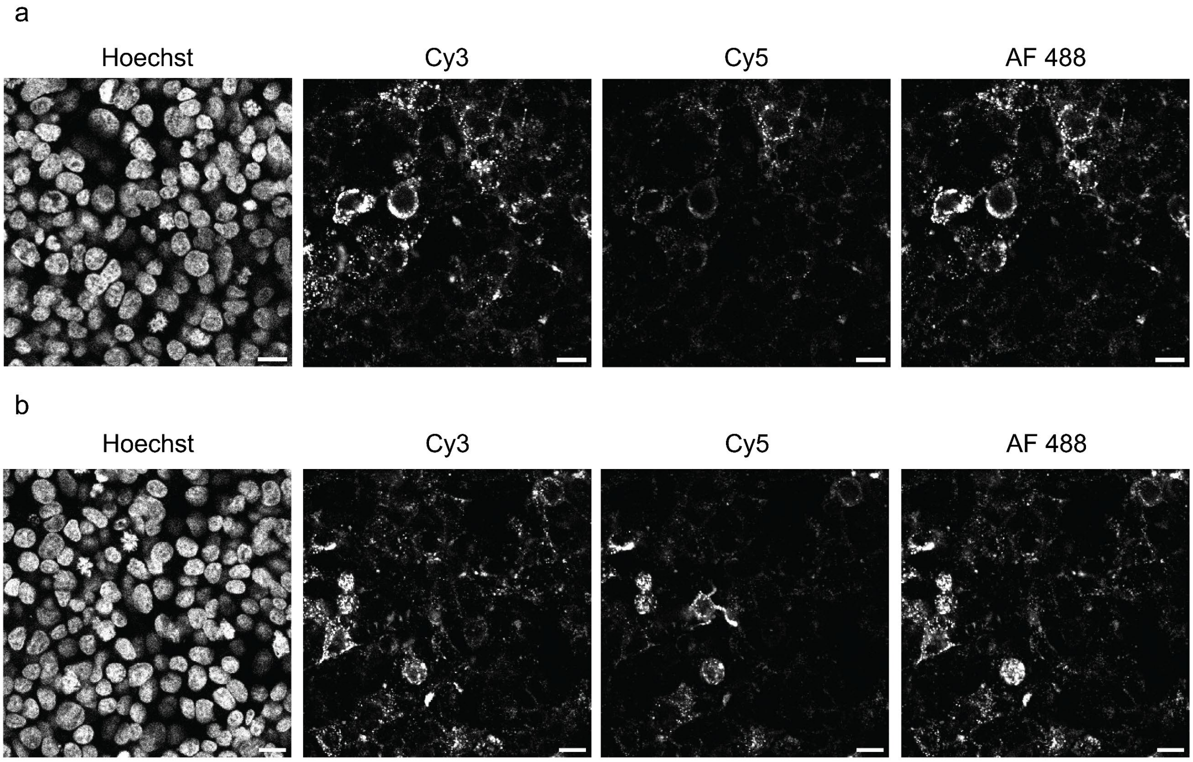

Supplementary Figure SR11: Raw, grayscale confocal micrograph images of A-, B-, and Γ-type$_{JAK/STAT}$ cells incubated with Cy3-labelled SUMO-tagged A’-A’, Cy5-labelled SUMO-tagged B’-B’, and AF 488-labelled SUMO-tagged Γ’-Γ’ dipeptide ligands. **a** corresponds to Supplementary Figure S19a and **b** corresponds to Supplementary Figure S19b and S19c. Scale bar (50 µm) is shown on the bottom-right of the image. Cy3 excitation: 553 nm, emission: 570-620 nm. Cy5 excitation: 647 nm, emission: 655-705 nm. AF 488 excitation: 488 nm, emission: 500-550 nm. Hoechst excitation: 405 nm, emission: 410-450 nm.

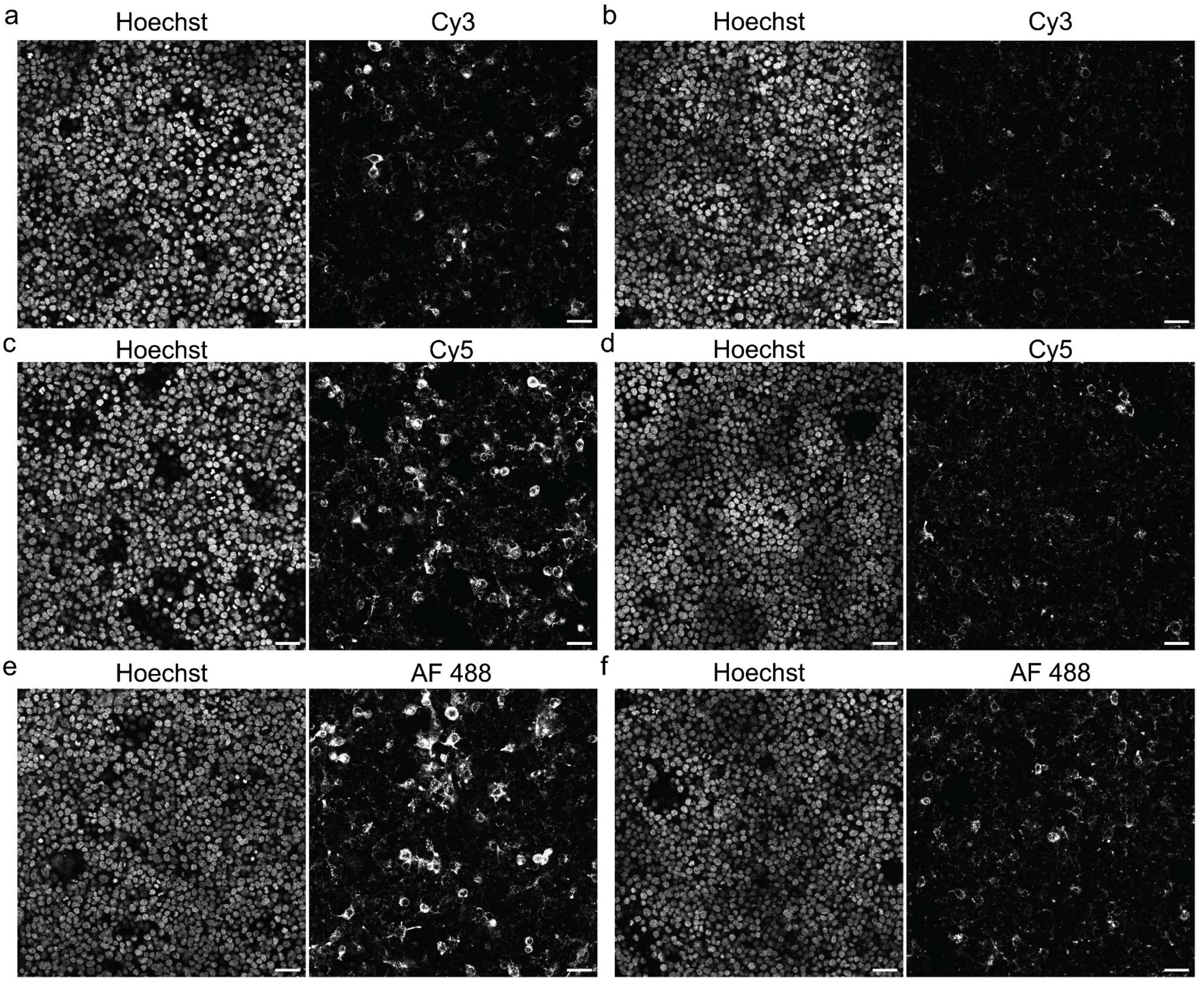

Supplementary Figure SR12: Raw, grayscale confocal micrograph images of **a** A-type$_{JAK/STAT}$ or **b** A-, B, Γ-type$_{JAK/STAT}$ cells incubated with Cy3-labelled SUMO-tagged A'-A' (corresponding to Supplementary Figure S21); **c** B-type$_{JAK/STAT}$ or **d** A-, B, Γ-type$_{JAK/STAT}$ cells incubated with Cy5-labelled SUMO-tagged B'-B' (corresponding to Supplementary Figure S22); **e** Γ-type$_{JAK/STAT}$ or **f** A-, B, Γ-type$_{JAK/STAT}$ cells incubated with AF 488-labelled SUMO-tagged Γ'-Γ' (corresponding to Supplementary Figure S23). Scale bar (50 µm) is shown on the bottom-right of the image. Cy3 excitation: 553 nm, emission: 570-620 nm. Cy5 excitation: 647 nm, emission: 655-705 nm. AF 488 excitation: 488 nm, emission: 500-550 nm. Hoechst excitation: 405 nm, emission: 410-450 nm.

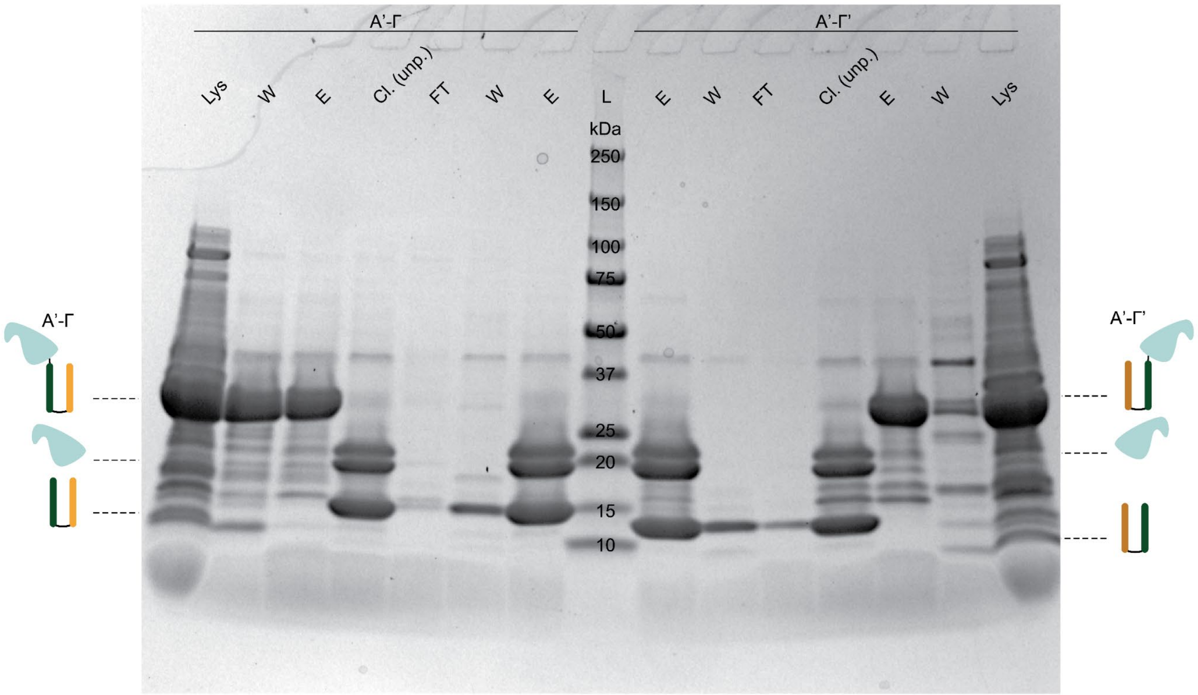

Supplementary Figure SR13: Raw SDS-PAGE gel image of Supplementary Figure S23. L: molecular weight ladder, Lys: cell lysate, W: wash fraction, FT: flow through, , E: elution fraction, Cl. (unp.): cleaved peptide (unpurified).

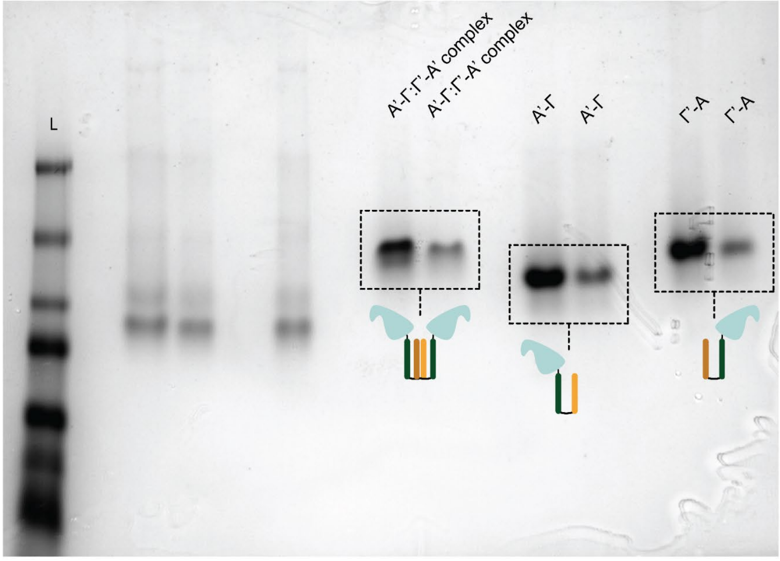

Supplementary Figure SR14: Raw SDS-PAGE gel image of Supplementary Figure S24b.

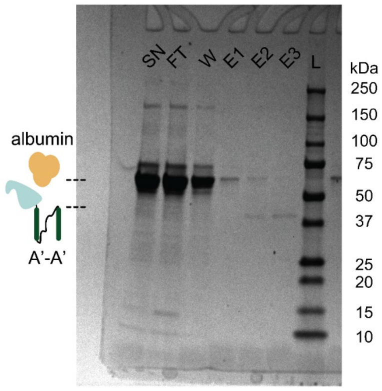

Supplementary Figure SR15: Raw SDS-PAGE gel image of Supplementary Figure S27. L: molecular weight ladder, SN: supernatant, FT: flow through, W: wash fraction, E1: elution fraction 1, E2: elution fraction 2, E3: elution fraction 3.

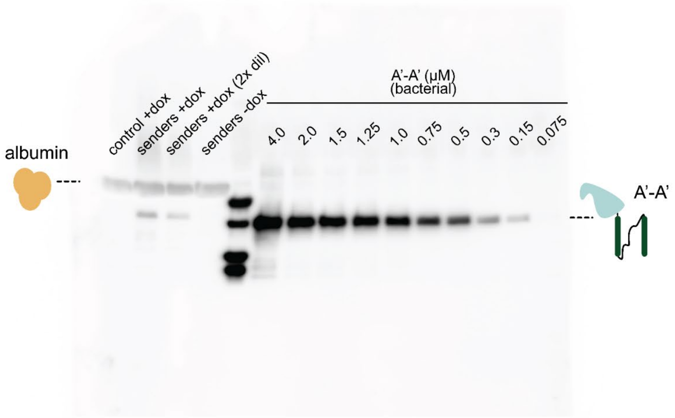

Supplementary Figure SR16: Raw image corresponding to Supplementary Figure S29.

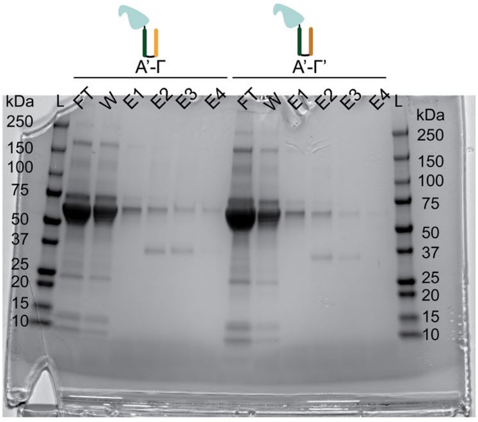

Supplementary Figure SR17: Raw SDS-PAGE gel image of Supplementary Figures S32 and S33. L: molecular weight ladder, FT: flow through, W: wash fraction, E1: elution fraction 1, E2: elution fraction 2, E3: elution fraction 3, , E4: elution fraction 4.

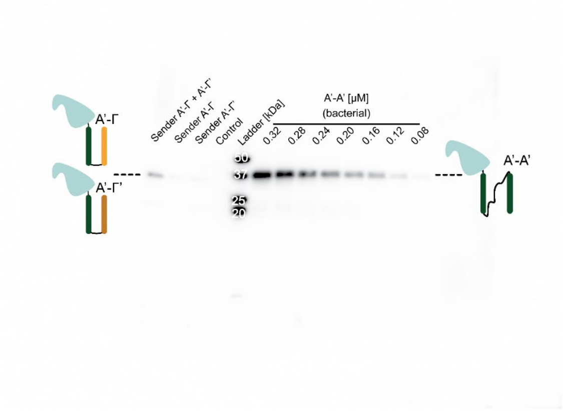

Supplementary Figure SR18: Raw image corresponding to Supplementary Figure S34.

References

1. Schukur, L., Geering, B., Charpin-El Hamri, G. & Fussenegger, M. Implantable synthetic cytokine converter cells with AND-gate logic treat experimental psoriasis. *Sci Transl Med* **7**, 318ra201-318ra201 (2015).

2. Ye, H., Baba, M. D.-E., Peng, R.-W. & Fussenegger, M. A Synthetic Optogenetic Transcription Device Enhances Blood-Glucose Homeostasis in Mice. *Science (1979)* **332**, 1565–1568 (2011).

3. Scheller, L., Strittmatter, T., Fuchs, D., Bojar, D. & Fussenegger, M. Generalized extracellular molecule sensor platform for programming cellular behavior. *Nat Chem Biol* **14**, 723–729 (2018).

4. Elegheert, J. *et al.* Lentiviral transduction of mammalian cells for fast, scalable and high-level production of soluble and membrane proteins. *Nat Protoc* **13**, 2991–3017 (2018).

5. van Rosmalen, M., Krom, M. & Merkx, M. Tuning the Flexibility of Glycine-Serine Linkers To Allow Rational Design of Multidomain Proteins. *Biochemistry* **56**, 6565–6574 (2017).

6. Golynskiy, M. V, Rurup, W. F. & Merkx, M. Antibody Detection by Using a FRET-Based Protein Conformational Switch. *ChemBioChem* **11**, 2264–2267 (2010).

7. Morsut, L. *et al.* Engineering Customized Cell Sensing and Response Behaviors Using Synthetic Notch Receptors. *Cell* **164**, 780–791 (2016).
