# Supplementary material for: Engineering a scalable and orthogonal platform for synthetic communication in mammalian cells
Source: Nat Commun. 2023 Nov 2;14:7001. doi: 10.1038/s41467-023-42810-5 (PMC10622552; doi:10.1038/s41467-023-42810-5)
Supplement: Supplementary file 2 — Reporting Summary [file 41467_2023_42810_MOESM2_ESM.pdf]

nature portfolio

Corresponding author(s): Tom F. A. de Greef

Last updated by author(s): Oct 16, 2023

Reporting Summary

Nature Portfolio wishes to improve the reproducibility of the work that we publish. This form provides structure for consistency and transparency in reporting. For further information on Nature Portfolio policies, see our Editorial Policies and the Editorial Policy Checklist.

Statistics

For all statistical analyses, confirm that the following items are present in the figure legend, table legend, main text, or Methods section.

n/a	Confirmed	
☐	☒	The exact sample size (*n*) for each experimental group/condition, given as a discrete number and unit of measurement
☐	☒	A statement on whether measurements were taken from distinct samples or whether the same sample was measured repeatedly
☐	☒	The statistical test(s) used AND whether they are one- or two-sided<br>*Only common tests should be described solely by name; describe more complex techniques in the Methods section.*
☒	☐	A description of all covariates tested
☐	☒	A description of any assumptions or corrections, such as tests of normality and adjustment for multiple comparisons
☐	☒	A full description of the statistical parameters including central tendency (e.g. means) or other basic estimates (e.g. regression coefficient) AND variation (e.g. standard deviation) or associated estimates of uncertainty (e.g. confidence intervals)
☐	☒	For null hypothesis testing, the test statistic (e.g. *F*, *t*, *r*) with confidence intervals, effect sizes, degrees of freedom and *P* value noted<br>*Give P values as exact values whenever suitable.*
☒	☐	For Bayesian analysis, information on the choice of priors and Markov chain Monte Carlo settings
☒	☐	For hierarchical and complex designs, identification of the appropriate level for tests and full reporting of outcomes
☒	☐	Estimates of effect sizes (e.g. Cohen's *d*, Pearson's *r*), indicating how they were calculated

Our web collection on statistics for biologists contains articles on many of the points above.

Software and code

Policy information about availability of computer code

Data collection	No software was used
Data analysis	Data were plotted with the use of MATLAB Toolbox Release R2019a for Windows (The MathWorks, Inc., Natick, Massachusetts, United States).<br>Statistical analysis was done with GraphPad Prism 9.4.1 for Windows (GraphPad Software, San Diego, California USA, www.graphpad.com).<br>Flow cytometry data were analysed with the use of FlowJoTM v10.7.1 Software (BD Life Sciences) for Windows.<br>For image analysis, the Leica LAS X software (version 5.1.0) and ImageJ (1.53t; Java 1.8.0_322 (64-bit)) were used.

For manuscripts utilizing custom algorithms or software that are central to the research but not yet described in published literature, software must be made available to editors and reviewers. We strongly encourage code deposition in a community repository (e.g. GitHub). See the Nature Portfolio guidelines for submitting code & software for further information.

Data

Policy information about availability of data

All manuscripts must include a data availability statement. This statement should provide the following information, where applicable:

- Accession codes, unique identifiers, or web links for publicly available datasets
- A description of any restrictions on data availability
- For clinical datasets or third party data, please ensure that the statement adheres to our policy

Datasets generated and/or analysed during the current study are available via Zenodo at https://zenodo.org/record/8055628 (10.5281/zenodo.10006328).

Research involving human participants, their data, or biological material

Policy information about studies with human participants or human data. See also policy information about sex, gender (identity/presentation), and sexual orientation and race, ethnicity and racism.

Reporting on sex and gender	n/a
Reporting on race, ethnicity, or other socially relevant groupings	n/a
Population characteristics	n/a
Recruitment	n/a
Ethics oversight	n/a

Note that full information on the approval of the study protocol must also be provided in the manuscript.

Field-specific reporting

Please select the one below that is the best fit for your research. If you are not sure, read the appropriate sections before making your selection.

- [x] Life sciences
- [ ] Behavioural & social sciences
- [ ] Ecological, evolutionary & environmental sciences

For a reference copy of the document with all sections, see nature.com/documents/nr-reporting-summary-flat.pdf

Life sciences study design

All studies must disclose on these points even when the disclosure is negative.

Sample size	For all experimental set-ups using cell lines, independent triplicates (n=3) were used; as it is conventionally used in experiments involving cell lines. Therefore, no sample size calculation was performed.
Data exclusions	No data were excluded
Replication	Reproducibility was verified by including independent triplicates (n=3) in experimental set-ups, using cell lines.
Randomization	n/a, as the experiments involved transected cell lines
Blinding	Blinding is not relevant during data collection and analysis, as experimenter bias cannot affect the study results.

Reporting for specific materials, systems and methods

We require information from authors about some types of materials, experimental systems and methods used in many studies. Here, indicate whether each material, system or method listed is relevant to your study. If you are not sure if a list item applies to your research, read the appropriate section before selecting a response.

Materials & experimental systems

n/a	Involved in the study	
[ ]	[x]	Antibodies
[ ]	[x]	Eukaryotic cell lines
[x]	[ ]	Palaeontology and archaeology
[x]	[ ]	Animals and other organisms
[x]	[ ]	Clinical data
[x]	[ ]	Dual use research of concern
[x]	[ ]	Plants

Methods

n/a	Involved in the study	
[x]	[ ]	ChIP-seq
[ ]	[x]	Flow cytometry
[x]	[ ]	MRI-based neuroimaging

Antibodies

Antibodies used

antibody against the SUMO-tag (Anti-Smt3 rabbit antibody; ab14405, abcam)
anti-rabbit IgG secondary antibody (HRP conjugate; #7074, Cell Signaling Technology)

Validation

Rabbit Polyclonal SMT3 antibody. Validated in ELISA, WB and tested in Saccharomyces cerevisiae samples. Cited in 13 publications, as stated in the manufacturer's website.
Anti-rabbit IgG, HRP-linked Antibody #7074; Designed for use with rabbit polyclonal and monoclonal antibodies, this affinity purified goat anti-rabbit IgG (heavy and light chain) antibody is conjugated to horseradish peroxidase(HRP) for chemiluminescent detection. This product is thoroughly validated with CST primary antibodies and will work optimally with the CST western immunoblotting protocol, ensuring accurate and reproducible results; as stated in the manufacturer's website.

Eukaryotic cell lines

Policy information about cell lines and Sex and Gender in Research

Cell line source(s)

HEK293T cells (CRL3216™) were purchased from ATCC®
HEK293S GnTI- TetR; obtained from the group of N. Callewaert at the VIB-UGent Center for Medical Biotechnology (nico.callewaert@ugent.vib.be)

Authentication

HEK293T cells (CRL3216™) were authenticated from ATCC®, by STR profiling

Mycoplasma contamination

HEK293T cells (CRL3216™) were tested for mycoplasma contamination by ATCC®. Additionally, cell lines used were later tested for mycoplasma contamination by a commercial provider and were found mycoplasma-free.

Commonly misidentified lines
(See ICLAC register)

No commonly misidentified cell lines were used in this study.

Plants

Seed stocks

n/a

Novel plant genotypes

n/a

Authentication

n/a

Flow Cytometry

Plots

Confirm that:

- [x] The axis labels state the marker and fluorochrome used (e.g. CD4-FITC).
- [x] The axis scales are clearly visible. Include numbers along axes only for bottom left plot of group (a 'group' is an analysis of identical markers).
- [x] All plots are contour plots with outliers or pseudocolor plots.
- [x] A numerical value for number of cells or percentage (with statistics) is provided.

Methodology

Sample preparation	HEK293T cells (CRL3216™) and HEK293S GnTI- TetR were used. Cell lines were either transiently transfected using a commercial transfection reagent or stably transfected with custom-made lentivirus (described in manuscript).
Instrument	Cell were interrogated and sorted on a FacsAria III (BD Biosciences)
Software	. Flow cytometry data were analysed with the use of FlowJoTM v10.7.1 Software (BD Life Sciences) for Windows.
Cell population abundance	Transfected cell population abundance is reported in the manuscript as a percentage of fluorescence-positive cells for each experiment.
Gating strategy	A total of 10,000-100,000 events were recorded, from which 2D plots of the side-scattered light area (SSC-A) versus forward-scattered light area (FSC-A), as well as FSC-A versus forward-scattered light height (FSC-H) were obtained. As denoted in the manuscipt captions, a first gate on FSC-A x SSC-A was constructed to identify the clonal population and a second gate on FSC-A x FSC-H was constructed to discriminate single cells from duplets.<br>Cells were sorted either as a positive (based on fluorescence), enriched polyclonal population or as single cells (to obtain monoclonal populations).

☒ Tick this box to confirm that a figure exemplifying the gating strategy is provided in the Supplementary Information.
