# Supplementary figures and images for: Engineering a scalable and orthogonal platform for synthetic communication in mammalian cells
Source: Nat Commun. 2023 Nov 2;14:7001. doi: 10.1038/s41467-023-42810-5 (PMC10622552; doi:10.1038/s41467-023-42810-5)

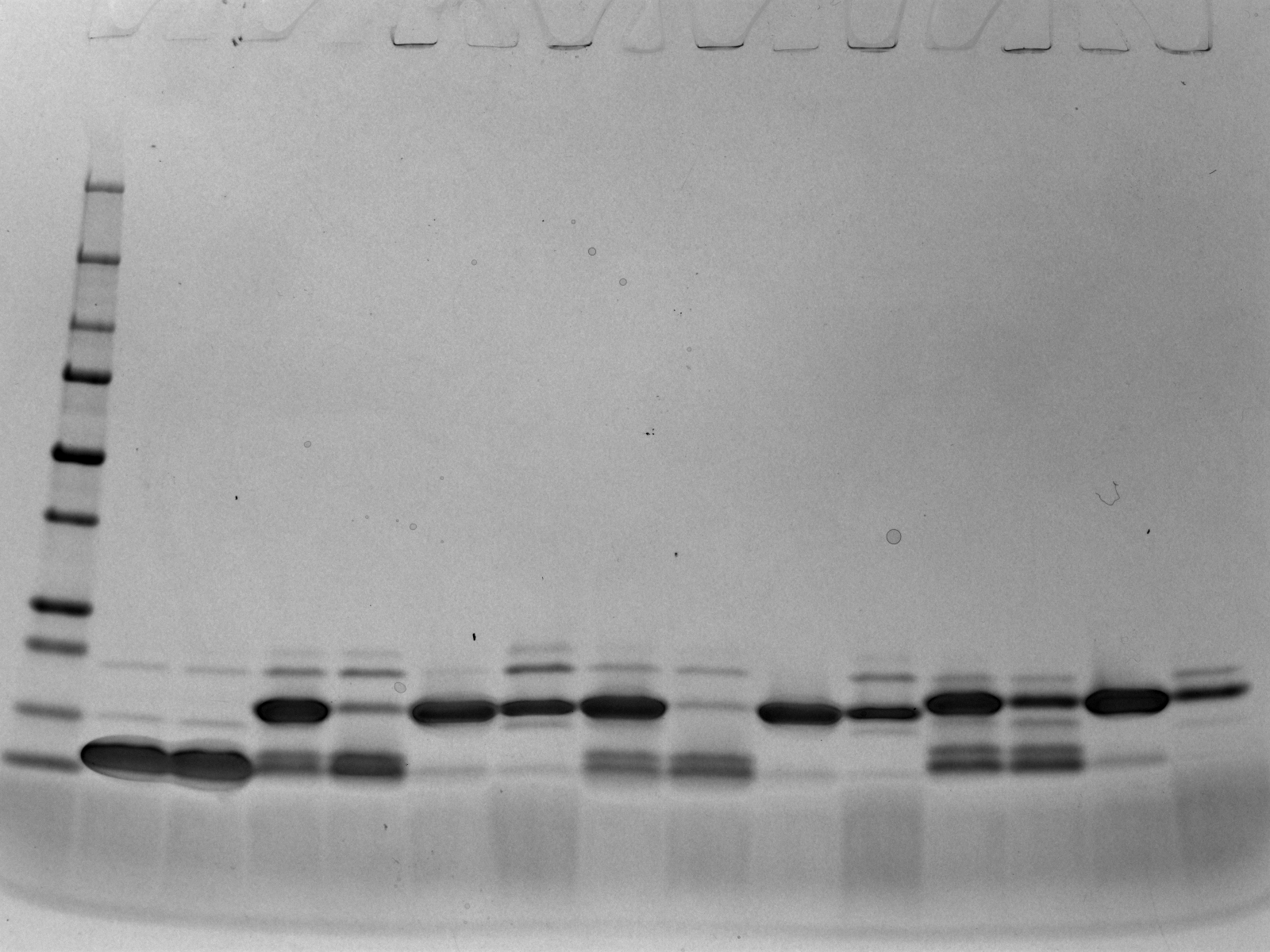

Supplement: Supplementary file 4 — Source Data [file 41467_2023_42810_MOESM4_ESM.zip › source data/Source data file_gel_Figure 2c.png]
